# Promising Directions: A Systematic Review of Psychosocial and Behavioral Interventions with Cultural Incorporation for Advanced and Metastatic Cancer

**DOI:** 10.1007/s12529-024-10264-8

**Published:** 2024-03-12

**Authors:** Karen Llave, Karli K. Cheng, Amy Ko, Annie Pham, Marissa Ericson, Belinda Campos, Hector R. Perez-Gilbe, Jacqueline H. J. Kim

**Affiliations:** 1 Department of Population Health & Preventive Disease, University of California, Irvine, USA; 2 Department of Medicine, University of California, 100 Theory, Suite 100, Irvine, CA 92697 USA; 3 Institute for Clinical and Translational Science, University of Southern California, Los Angeles, USA; 4 Department of Chicano/Latino Studies, University of California, Irvine, USA; 5 UCI Libraries, University of California, Irvine, USA; 6 Chao Family Comprehensive Cancer Center, University of California, Irvine, USA

**Keywords:** Advanced cancer, Metastatic cancer, Culture, Psychosocial intervention, Underserved Populations, Survivorship

## Abstract

**Background:**

Improving quality of life (QOL) in advanced and metastatic cancer is a priority with increasing survivorship. This systematic review synthesizes psychosocial and behavioral interventions incorporating culture with the goal of examining their benefit for understudied and medically underserved populations with advanced and metastatic cancer.

**Method:**

Reports were systematically screened for (1) a focus on advanced and metastatic cancer survivors, (2) psychosocial or behavioral intervention intended to improve QOL, (3) evidence of incorporating the culture(s) of understudied/underserved populations, and (4) availability in English. Bias was evaluated using the JBI Critical Appraisal Checklist and the Methodological index for non‐randomized studies. Qualitative synthesis and quantitative meta-analyses were completed.

**Results:**

Eighty-six reports containing 5981 participants’ data were examined. Qualitative synthesis of 23 studies identified four overarching themes relevant for incorporating culture in interventions. Meta-analysis of 19 RCTs and 4 quasi-experimental studies containing considerable heterogeneity indicated greater improvements in QOL (*g* = 0.84), eudaimonic well-being (*g* = 0.53), distress (*g* = −0.49), and anxiety (*g* = −0.37) for main intervention conditions compared to controls. Meta-analysis of 10 single-arm trials containing minimal to moderate heterogeneity found benefit for anxiety (*g* = −0.54), physical symptoms (*g* = −0.39), and depression (*g* = −0.38).

**Conclusion:**

Psychosocial and behavioral interventions with cultural incorporation appear beneficial for improving QOL-related outcomes in advanced and metastatic cancer. Studies incorporating culture in psychosocial or behavioral interventions offer noteworthy insight and suggestions for future efforts such as attending to deep cultural structure.

**Supplementary Information:**

The online version contains supplementary material available at 10.1007/s12529-024-10264-8.

## Introduction

People living with advanced and metastatic cancer have unique psychosocial needs and, with longer survival times due to treatment advancements, a greater focus on survivorship care is needed. Having advanced and metastatic cancer involves facing the possibility of incurable disease, with continued treatment and monitoring until the end of life [1]. The number of individuals living with metastatic cancer will increase to nearly 700,000 by 2025 in the USA alone [2]. Medically underserved or understudied populations which include racial and ethnic minority groups, people with lower socioeconomic status, underserved rural communities, sexual and gender minority groups, and people with disabilities are at higher risk for diagnosis at advanced and metastatic stages [3, 4]. Advanced and metastatic cancer survivors experience physical and psychosocial challenges resulting from long-term symptoms related to prolonged treatment [5]. The psychological impact of later-stage disease is different from earlier-stage cancer patients, including greater risks for adverse outcomes such as suicidality [6, 7]. Adverse outcomes may be prevented with psychosocial or behavioral interventions defined as “interpersonal or informational activities, techniques, or strategies that target biological, behavioral, cognitive, emotional, interpersonal, social, or environmental factors” to improve “health functioning and well-being” [8]. Effective psychosocial and behavioral interventions are needed to improve patient-reported outcomes, including quality of life (QOL), which can predict progression-free survival above and beyond tumor markers in advanced cancer [9].

Culture is vital in designing and implementing effective health-related interventions [10]. Culture is the dynamic framework underlying all human endeavors that shapes how members of a group collectively see and experience the world around them [11]. For all people, culture is a lens that shapes one’s priorities and goals as well as one’s experience and evaluation of others’ actions and social events [12]. Culture helps individuals navigate relationships with others, organizations, and the environments around them [13, 14]. Therefore, culture can significantly influence how individuals experience and live with cancer. For example, studies have shown the heightened importance of maintaining hope and including family in care discussions among Latino cancer patients compared to US-born White non-Latinos. However, healthcare providers face challenges initiating discussions about prognosis and palliative care with Latino patients and caregivers due to cultural preferences to focus on hope and optimism for a cure rather than palliative and comfort care discussions [15, 16]. Such challenges can be ameliorated by culturally aligned psychosocial interventions, leading to positive participant experience, and improved outcomes [17]. Further, previous meta-analyses have broadly demonstrated the benefit of cultural adaptations in psychological treatments and interventions such as psychotherapy [18–20]. Considering the growing number of advanced and metastatic cancer survivors, effective and culturally appropriate psychosocial or behavioral interventions are critical [9].

### Cultural Incorporation in Psychosocial or Behavioral Intervention Research

Approaches to cultural incorporation in psychosocial or behavioral intervention research involve diverse methods. The most well-known methods use theoretically based adaptation models/frameworks including Bernal’s ecological validity model [21], and, generally, there are recommendations for the “what” and “how” of culturally adapting an existing intervention. Some examples for “what” to adapt in interventions include language, persons, metaphors, content, concepts, goals, methods, context [22], and concepts of distress, treatment components, and delivery [21]. Other frameworks describe the manner in which the interventionist would adapt to the recipient as part of the therapeutic interaction [23]. Cultural adaptations also involve iterative steps such as building a collaboration with the community of focus, assessing intervention interest and needs with community leaders, developing and piloting the adapted intervention, observing adaptations and collecting feedback [24], and incorporating community-based knowledge from interested parties [25].

Despite variation across suggestions for cultural incorporation, adaptation, and tailoring [26], there are commonalities. Firstly, intervention development can be conducted top-down or bottom-up. A top-down design is based on existing mainstream interventions and utilizes a universal approach assuming the original intervention is applicable to all subgroups as is or with cultural adaptations. Conversely, a bottom-up design utilizes a culture-specific approach which considers emic cultural theories of problem and change, utilizes community-generated data or tradition-based practices, and at times informs a novel intervention that may not yet be evidence-based [27]. Secondly, the cultural congruence considered may be at a surface or deep level [28]. Surface-level cultural match increases the acceptability or feasibility by focusing on the "observable social or cultural" behaviors [29], for example, the language or setting in which the intervention is conducted. On the other hand, deep-level cultural match increases engagement by focusing on shared understandings including how social, cultural, and historical environments influence behavior [29], for example, taking into consideration a cultural group’s distinctive practices for maintaining health, strong ties to religion, or historically negative relationship with the government or healthcare system. Thirdly, there are specific elements of the intervention outlined for adaptation such as content (presented as part of the intervention), process (how interventionist interacts with the recipient), and delivery (how intervention is delivered).

Cultural incorporation and adaptation are particularly relevant for understudied and underrepresented populations; however, the majority of previous behavioral health interventions have been developed predominantly through a European American lens or with Western, Educated, Industrialized, Rich, Democratic (WEIRD) populations based in North America [11, 30–32]. A 2020 National Institutes of Health (NIH) portfolio review of cancer survivorship research revealed a dearth of focus on advanced and metastatic cancer populations. Of the twenty-five studies focused on advanced and metastatic cancer survivorship, only nine studies centered on minoritized or medically underserved populations such as African Americans and Hispanic/Latinos, as well as older adults and low-income and socioeconomically disadvantaged groups [33]. Given the limited representation of understudied groups and non-WEIRD populations in behavioral health research, the goal of the present review is to focus on advanced and metastatic cancer populations outside of well-researched WEIRD countries while still prioritizing medically underserved and understudied groups (e.g., ethnoracial minorities, adolescents and younger adults (AYA), rural) in WEIRD countries [32].

This systematic review summarizes psychosocial and behavioral interventions incorporating culture, including those designed for understudied and underserved populations living with advanced and metastatic cancer. The specific aims of this systematic review are to (1) identify psychosocial or behavioral interventions with intentional cultural adaptations/components, (2) describe the approaches to cultural adaptation or developing culturally based interventions, (3) describe the progress for understudied/underserved populations and which groups remain understudied, (4) identify gaps and opportunities for psychosocial or behavioral intervention science for those living with advanced and metastatic cancer, and (5) examine the impact of these interventions on intended psychosocial outcomes if availability of data allowed so. This review is registered with the International Prospective Register of Systematic Reviews (PROSPERO ID: CRD42022375667).

## Methods

### Search Strategy

A psycho-oncology researcher (JK), public health researcher (KL), and medical librarian (HPG) developed a comprehensive search strategy based on the research question to identify relevant literature. Keywords were selected by using the PICO framework, medical subject headings database, and relevant literature during search preparation. A broad search strategy was used to identify all potentially relevant studies to the cancer population of focus. The search was primarily based on the “population” and “intervention” search terms as “comparison” and “outcome” search terms limited the results and exemplary studies would be missed. Final search terms consisted of MESH terms and free-text keywords combined with Boolean operators related to advanced and metastatic cancer, psychosocial and behavioral interventions, and cultural adaptation/tailoring.^1^ The search was conducted November to December 2022 by HPG in PubMed, CINAHL, Scopus, PsycInfo, and Cochrane Library and completed on December 21, 2022. Review articles published in the past two years were manually searched for any missed articles. Results were imported into Covidence and duplicates were removed.

### Inclusion and Exclusion Criteria

Manuscripts were screened using the following inclusion criteria: (1) centered on advanced and metastatic cancer survivors or a majority sample of advanced and metastatic cancer survivors, (2) described a psychosocial intervention design/adaptation intended to improve psychosocial or behavioral components related to QOL in advanced and metastatic cancer, (3) evidence of incorporating non-WEIRD cultural context OR understudied/underserved populations’ contexts in WEIRD societies in the intervention design/adaptation efforts, and (4) was available in English.

### Screening and Selection

Screening was conducted by a group of trained reviewers composed of students, research specialists, and faculty. Titles and abstracts were independently screened for inclusion by at least two members of the reviewer group. Potentially relevant sources identified in the title/abstract stage were moved to the full text review stage. The full texts were assessed in detail against the inclusion criteria by one member of the authorship group and one additional reviewer. Reasons for exclusion at the full text level were recorded. Any disagreements that arose between the reviewers at each stage of the selection process were resolved by a third reviewer or discussed as a team with JK and BC until consensus was reached.

### Data Extraction and Coding

Study design, sample characteristics, quantitative data, intervention details (e.g., format, setting, delivery), and cultural incorporation methods (i.e., use of adaptation model or framework, top-down or bottom-up design, and surface- and deep-level cultural congruence) were extracted by two independent reviewers within Covidence. Conflicts were resolved by a third reviewer or discussed with JK and BC until consensus was reached. Quantitative data points for extraction were identified in consultation with a biostatistician (ME).

### Data Analysis

#### Qualitative Synthesis

Qualitative thematic synthesis was conducted per usual standards in health-related systematic reviews [34, 35]. All text under the “Results/Findings”, “Discussion” and “Conclusion” sections for 23 qualitative studies were imported into Atlas.ti for qualitative coding. Qualitative data from non-patient participants were included if the focus was on patient-oriented interventions. Two trained researchers (AP, JK) independently coded the imported qualitative data and evaluated the quality of each manuscript for sensitivity analysis [36, 37] simultaneously [38, 39]. Coders focused on answering the question “What are the cultural considerations/adaptations suggested for psychosocial or behavioral interventions in advanced and metastatic cancer?” and resolved coding disagreements with discussion. Codes were grouped into themes and subthemes. The stability of themes was re-examined with and without poorer quality studies determined by sensitivity analyses [36, 37] (see Supplementary Appendix B).

#### Meta-analytic Methods

Although limited, the study team identified an adequate number of studies reporting patient-focused quantitative data for exploratory meta-analyses. Two types of meta-analyses were completed: (1) the effect size for how much the main intervention group benefited for a psychosocial outcome vs. the control group with RCT and quasi-experimental reports and (2) the effect size for single-arm trials from pre-intervention to post-intervention. The first data point available post-intervention was utilized, as psychosocial changes are the most significant closest to the end of an intervention [40]. Analyses were conducted using R (v.4.2.1). A random-effects estimation model was used with Hedge’s *g*, which adjusts for small sample bias. Only studies adequately reporting means and standard deviations across groups and time points were included. Corresponding authors were contacted to obtain missing data points, but 12 studies were excluded from analyses due to nonresponse. Heterogeneity among studies was assessed using Cochran’s *Q* and *I*^2^ [41]. Publication bias was evaluated using Egger’s test of funnel plot asymmetry for outcomes with 10 or more studies, as recommended [42]. We conducted exploratory moderator analyses comparing the effect sizes of studies based on types of cultural incorporation, when there was at least four studies per subgroup as recommended [43].

### Risk of Bias for Included RCTs and Single-Arm Trials

Two trained reviewers (KL, AP) independently assessed the methodological quality of the included studies using the JBI Critical Appraisal Checklist for RCTS [44] and the methodological index for non‐randomized studies (MINORS) [45]. Disagreements were resolved through discussion or by a third author (JK). No studies were excluded based on the quality appraisal score as recommended [44]. Ratings are in Supplementary Appendix B.

## Results

**Fig. 1:**
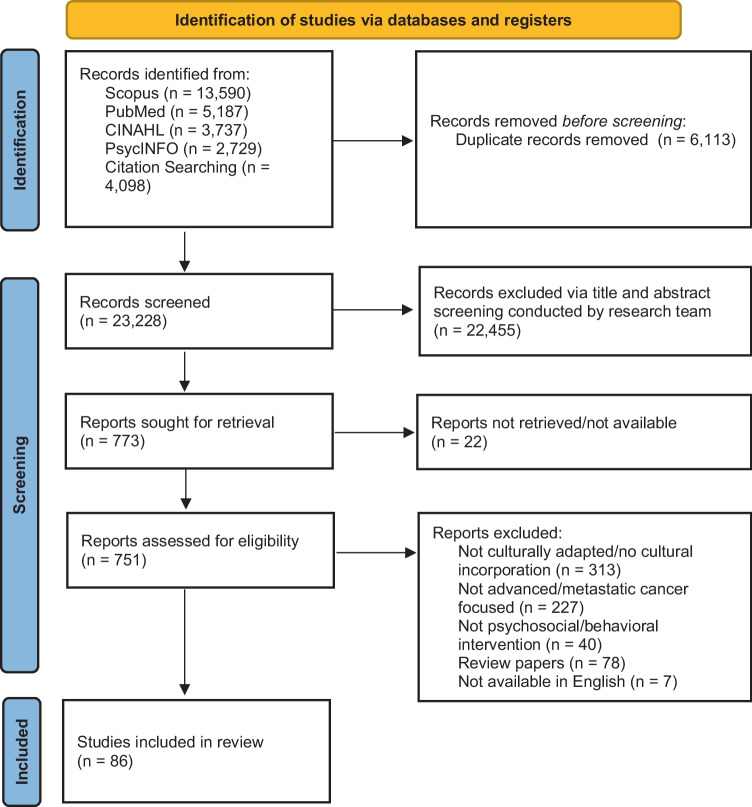
PRISMA flow diagram

A total of 86 reports were identified and included in the review; 35 randomized controlled trials, 14 qualitative studies, 11 single-arm studies, 8 quasi-experimental studies, and 7 protocols, 7 feasibility/acceptability trials, and 4 mixed-methods studies (see Fig. 1). Over half (*n* = 49, including 3 protocols) of the reports reviewed were conducted with populations in non-WEIRD countries, and 38 reports were conducted in WEIRD countries with ethnic minority and medically underserved populations. A total of 73 interventions were described, twelve of which were described in multiple reports. A majority of the studies were conducted in China (*n* = 20), USA (*n* = 17), Japan (*n* = 10), and South Korea (*n* = 4), and the most commonly used languages were Chinese, English, Spanish, and Japanese (see Supplementary Appendix C for full list).

### Participant Characteristics

A total of 5981 participants were examined across the 80 reports,^2^ and 4620 participants were identified as having stage 3, stage 4, terminal/end-stage cancer, and advanced and metastatic cancer. Four hundred fifty-six cancer survivors’ cancer staging was not described, and eight reports with a majority sample of advanced and metastatic cancer also included 152 participants with non-advanced and non-metastatic cancer. The remaining 753 participants included caregivers (*n* = 477), lay health workers (*n* = 195), healthcare professionals (*n* = 67), and community stakeholders (*n* = 14). The intervention and control groups had mean sample sizes of 37 (range 7–112) and 39 (range 10–111), respectively. Participants with a cancer diagnosis were 59.38 years old on average; 53.62% were women.^3^ Caregivers were 53.81 years old on average; 75.98% were women. From available healthcare professional participant data,^4^ 84.00% were women. Twenty-three reports included race and ethnicity of participants with cancer where 815 were White (36.63%), 451 were Latino/a/x or Hispanic (20.27%), 442 were African American/Black (19.87%), 289 were Asian (12.89%) and 151 were reported as an aggregate Asian/Pacific Islander group (6.74%). The most common cancer types were lung, breast, gastrointestinal, and prostate; see Supplementary Appendix C for all cancer types. In line with prior research on advanced and metastatic cancer populations, the majority of reports in this review (*n* = 50) focused on people nearing end of life or in palliative care.

### Intervention Characteristics (Settings, Type, Format)

The majority of reports recruited from clinical (e.g., palliative care centers, outpatient clinics) and non-clinical community settings (*n* = 56, 69.14%), followed by clinical inpatient settings (*n* = 25, 30.86%, e.g., hospitalization), and five studies (6.17%) from both outpatient and inpatient settings. Interventions conducted in non-clinical settings included the participant’s home (*n* = 15), web-based (*n* = 11), community center (*n* = 1), and 12 studies conducted in more than one setting.

The psychosocial and behavioral interventions included in this review were grouped into 12 intervention approaches (see Table 1), with the three most common being (1) dignity therapy, life review, and narrative-based (*n* = 23), (2) a mix of two or more approaches (*n* = 15), and (3) education/psychoeducation (*n* = 14).

**Table 1:** Intervention and patient participant characteristics

**Characteristic**	**M(SD) or** ***n*** **(%)**
Number of intervention sessions^a^	5.89 (6.96)
Intervention type	
Dignity therapy or life review/narrative	23 (26.74%)
Mixed type (e.g., Naikan and Morita therapies, mindfulness and art therapy, cognitive behavioral and acceptance and commitment therapies)	15 (17.44%)
Education/psychoeducation	14 (16.28%)
Cognitive behavioral therapy (CBT)-based	7 (8.14%)
Meaning-centered psychotherapy (MCP)	7 (8.14%)
Complementary and alternative medicine	6 (6.98%)
Semi-structured supportive-expressive psychotherapy (CALM)	3 (3.45%)
Expressive arts (music, writing, other)	3 (3.45%)
Spiritual care	3 (3.45%)
Physical activity	2 (2.30%)
Self-help	2 (2.30%)
Peer support	1 (1.16%)
Cancer stage of participants	
Stage I (from studies that were majority advanced and metastatic cancer)	46 (0.90%)
Stage II (from studies that were majority advanced and metastatic cancer)	69 (1.36%)
Stage III	619 (12.18%)
Stage IV	1284 (25.26%)
Non-metastasis	37 (0.73%)
Metastasis	311 (6.12%)
Terminal/end stage	689 (13.55%)
Advanced and metastatic^b^	2028 (39.90%)

^a^The following studies did not report the total number of intervention sessions and were thus *excluded* from calculating the mean: Catania et al. [68]; Delrieu et al. [69]; Han et al. [70]; Hanson et al. [71]; Houmann et al. [72]; Ichihara et al. [73]; Lin et al. [74]; Maungtoug et al. [75]; Molassiotis et al. [76]; Nakayama et al. [77]; Nunziante et al. [78]; Patel et al. [79–81]; and Schulman-Green et al. [82] (see Supplementary Appendix G)

^b^Includes one instance of overlap in count (*n* = 63) from Anderson et al. (2004) [83]

Interventions were delivered face-to-face (*n* = 66, 76.74%), remotely (*n* = 10, 11.63%), and a hybrid of both (*n* = 6, 6.98%). Four qualitative studies (4.59%) evaluated community needs and preferences prior to adaptations and did not describe any intervention delivery methods. The most common remote methods of delivery were telemedicine (*n* = 6), instant messaging (*n* = 3, e.g., WeChat), and one mobile game.

Interventions were delivered by clinicians (*n* = 22, e.g., physicians, psychiatrists, nurses, social workers, dieticians), trained facilitators (*n* = 52, e.g., music therapists, yoga specialists, traditional Chinese medicine practitioners, mindfulness practitioners, research students, lay navigators, lay health workers), trained peer mentors and community volunteers (*n* = 5), and four studies included a team of clinicians and trained facilitators. For intervention details including refusal/attrition rates and intervention dosage, see Supplementary Appendix D.

### Adaptation Models/Frameworks

Seven studies applied an established model/framework to guide cultural adaptation of existing interventions. The models/frameworks used include the ecological validity model (*n* = 5), cultural adaptation process model (*n* = 2), and sunrise model (*n* = 1). One report applied both the ecological validity model and the cultural adaptation process model.

### Cultural Considerations/Adaptations (Table 2 and Supplementary Appendix E)

**Table 2:** Included psychosocial or behavioral interventions in advanced and metastatic cancer by study type

**Author, year**	**Intervention** **• Cultural incorporation/adaptation beyond language** ^**a**^	**Sample description**	**Intervention group (IG) & control group (CG)**
***Qualitative studies***			
Bekelman et al. (2019)^b^	Puente para cuidar (bridge to caring) • **CI/ABL: Yes.**	Latino/a patients; stage III/IV cancer; USA.	IG: Puente para cuidar (bridge to caring) intervention, combining the CASA (Collaborative Care to Alleviate Symptoms and Adjust to Illness) counseling intervention and Apoyo patient navigator interventions. CG: N/A (all participants received intervention).
Chen et al. (2020)	Mind map-based life review program **• CI/ABL: Yes.**	Hospital oncology chemotherapy patients; China.	IG: Mind Map-Based Life Review Program. CG: Routine care.
Chen et al. (2022)^b^	WeChat-based dyadic life review program **• CI/ABL: Yes.**	Hospital oncology chemotherapy patient-family caregiver dyads; advanced cancer; China.	IG: Routine care plus the WeChat-based Dyadic Life Review Program. CG: Routine care.
Fink et al. (2020)	Apoyo con Cariño (support with caring) • **CI/ABL: Yes.**	Bilingual and bicultural lay patient navigators; advanced cancer; USA.	IG: Culturally tailored information packet plus Apoyo con Cariño (support with caring) intervention. CG: Culturally tailored packet of written information about advance care planning, pain management, hospice use, and a study-specific advance directive.
Houmann et al. (2010)^c^	Dignity therapy • **CI/ABL: Yes.**	Gynecologic oncology or palliative care health professionals from hospital or hospice. Palliative medicine, hospice, and oncology patients; incurable cancer; Denmark.	IG: Dignity therapy and a generativity document. CG: N/A (all participants received intervention).
Kwan et al. (2019)^b^	Short-term life review • **CI/ABL: Yes.**	Hospitalized, day hospice or outreach home palliative care patients; life-limiting diseases; Hong Kong.	IG: Life review intervention and a personalized life review booklet. CG: Placebo questions on symptoms and social issues.
Leng et al. (2018); Leng et al. (2019)	Meaning-centered psychotherapy (MCP-Ch) • **CI/ABL: Yes.**	2018 study: Community leaders and health professionals; USA. 2019 study: Chinese immigrants; stage IV cancer; USA.	N/A. No IG & CG. 2018 study interviewed community leaders and health professionals to adapt intervention. 2019 study interviewed patients for their perspectives on intervention.
Li et al. (2014)	Dignity therapy • **CI/ABL: Yes.**	Palliative care inpatient and home service patients and healthcare professionals; end-stage cancer; Taiwan.	IG: Dignity therapy and a generativity document. CG: N/A (all participants received intervention).
Lin et al. (2020)	Advanced care planning intervention • **CI/ABL: Yes.**	Inpatient, outpatient, hospice, and palliative care patients; metastatic cancer; Taiwan.	IG: Advanced care planning intervention. CG: N/A (all participants received intervention).
Lin et al. (2022)	Dignity therapy • **CI/ABL: No.**	Therapists for terminal cancer patients; China.	N/A. No IG & CG. Study interviewed dignity therapists on their perspectives and culture-specific experiences.
Liossi et al. (2001)^b^	Hypnosis • **CI/ABL: No.**	Palliative care patients; far-advanced cancer; Greece.	IG: Hypnosis in addition to the standard medical and psychological support. CG: Standard medical and psychological support.
Nunziante et al. (2021)	Dignity therapy • **CI/ABL: No.**	Hospital palliative care and oncology patients; incurable cancer; Italy.	IG: Dignity therapy and a generativity document. CG: N/A (all participants received intervention).
Ólafsdóttir et al. (2018)	Advanced care planning intervention • **CI/ABL: No.**	Outpatient oncology patients; advanced lung cancer; Iceland.	IG: Advanced care planning and the usual hospital palliative care consulting team. CG: N/A (all participants received intervention).
Patel et al. (2019)	Lay health workers educate engage and encourage patients to share (LEAPS) cancer care • **CI/ABL: Yes.**	Low-income and minority hour-wage workers cancer patients; end-of-life cancer; Atlantic City, New Jersey and Chicago, Illinois.	N/A. No IG & CG. Study interviewed patients, caregivers, healthcare providers, and community advisory board to adapt intervention.
Pon (2010)	My Wonderful Life Board Game • **CI/ABL: Yes.**	Hospital hospice patients; advanced cancer; China.	IG: My Wonderful Life Board Game intervention. CG: N/A (all participants received intervention).
Schulman-Green et al. (2022)	Managing cancer care • **CI/ABL: No.**	Oncology center patients and their family caregivers; stage III/IV breast cancer; Israel.	N/A. No IG & CG. Study interviewed patients and caregivers to adapt intervention.
Takenouchi et al. (2022)	Lifeline interview method • **CI/ABL: Yes.**	Outpatient chemotherapy patients; incurable lung cancer; Japan.	IG: Lifeline interview method. CG: N/A (all participants received intervention).
Torres-Blasco et al. (2022)	Caregiver–patient support to Latinx coping advanced cancer (CASA) • **CI/ABL: Yes.**	Oncology Latinx patients and their caregivers; advanced cancer; Puerto Rico.	N/A. No IG & CG. Study interviewed patients and caregivers to adapt intervention.
van den Hurk et al. (2015)^b^	Mindfulness-based stress reduction • **CI/ABL: No.**	Tertiary care medical center patients and their partners; 74% stage IIIb, IV; lung cancer; The Netherlands.	IG: Mindfulness-based stress reduction training and practiced at home daily. CG: N/A (all participants received intervention).
Wang et al. (2019)^b^	Family participatory dignity therapy • **CI/ABL: Yes.**	Hematologic neoplasms patients and their family caregivers; China.	IG: Family participatory dignity therapy program. CG: N/A (all participants received intervention).
Xiao et al. (2012)	Life review program • **CI/ABL: Yes.**	Home-based hospice patients; advanced cancer; China.	IG: Routine care plus the life review program. CG: Routine care through home visits and weekly telephone follow-up.
Yang et al. (2021)	Educate, Nurture, Advise, Before Life Ends (ENABLE) • **CI/ABL: Yes.**	Oncology and palliative outpatient patients, family caregivers, and healthcare professionals; stage IV solid tumor cancer; Singapore.	IG: Intervention based on the principles of problem-solving, coping, decision-making, advance care planning, symptom management, self-care, communication, and life outlook and review. CG: N/A (all participants received intervention).
***Randomized control studies***			
Anderson et al. (2004)^c^	Pain education • **CI/ABL: Yes.**	Low-income, outpatient oncology African American and Hispanic/Latino patients; chronic cancer-related pain; 65% metastatic cancer; USA.	IG: Pain education specific for underserved African American men and women and Hispanic men and women. CG: Nutrition education package.
Ando et al. (2010)	Short-term life review • **CI/ABL: No.**	Hospital palliative care patients; terminally ill cancer; Japan.	IG: Short-term life review. CG: General support.
Bakitas et al. (2009)^c^	Educate, Nurture, Advise, Before Life Ends (ENABLE II) • **CI/ABL: Yes.**	Patients from oncology clinics and Veterans Affairs medical center; advanced cancer; USA.	IG: Educational manual. CG: Usual care.
Bouchard et al. (2019)^c^	Cognitive behavioral stress management • **CI/ABL: Yes.**	Men from medical centers and Veterans Affairs medical center; advanced prostate cancer; USA.	IG: Cognitive behavioral stress management intervention. CG: Attention-matched health promotion.
Caruso et al. (2020)^c^	Managing Cancer and Living Meaningfully (CALM) • **CI/ABL: No.**	Patients from psycho-oncology psychiatry in a university palliative care; advanced cancer; Italy.	IG: Managing Cancer and Living Meaningfully intervention. CG: Unstructured psychological support from a different psychotherapist.
Chen et al. (2020); Chen et al. (2022)	Mind map-based life review program • **CI/ABL: Yes.**	Hospital oncology stage III/IV cancer patients; China.	IG: Mind Map-Based Life Review Program. CG: Routine care.
Cheung et al. (2020)^c^	Self-administered acupressure • **CI/ABL: Yes.**	Hospital oncology department patients; advanced stage cancer; Hong Kong.	IG: Training on self-administered acupressure and practiced daily. CG: Usual care and a health talk unrelated to symptom management.
Cheung et al. (2021)	Aerobic exercise & tai chi • **CI/ABL: Yes.**	Stage IIIB/IV non-small cell lung cancer patients; Hong Kong.	IG 1: Aerobic exercise classes and self-practiced. IG 2: Tai chi classes and self-practiced. CG: Written information on the recommended levels of physical activity for self-management.
Dionne-Odom et al. (2021)	ENABLE (Educate, Nurture, Advise, Before Life Ends) Cornerstone • **CI/ABL: Yes.**	African American and rural family caregivers and patients; advanced cancer; USA.	IG: Phone coaching sessions of ENABLE Cornerstone. CG: Usual care.
Du et al. (2022)^c^	Heart to Heart Card Game • **CI/ABL: Yes.**	Home-based palliative care patients; stage III/IV cancer; China.	IG: Heart to Heart Card Game intervention. CG: Routine palliative care.
Fischer et al. (2018)^c^	Apoyo con Cariño (Support With Caring) • **CI/ABL: Yes.**	Latino patients; stage III/IV cancer; USA.	IG: Culturally tailored information packet plus Apoyo con Cariño (support with caring) intervention. CG: Culturally tailored packet of written information about advance care planning, pain management, hospice use, and a study-specific advance directive.
Fraguell-Hernando et al. (2020)	Individual meaning-centered psychotherapy-palliative care • **CI/ABL: No.**	Home palliative care patients; advanced stage cancer; Spain.	IG: Individual MCP-palliative care. CG: Standard psychotherapeutic counseling sessions.
Gil et al. (2018)^c^	Meaning-centered psychotherapy-compassionate palliative care • **CI/ABL: No.**	Hospital psycho-oncology patients; terminally ill; Spain.	IG 1: MCP-compassionate palliative care. IG 2: MCP-palliative care version. CG: Standard counseling focusing on coping by sharing illness and/or treatment-related concerns.
Han et al. (2021)	Naikan and Morita Therapies • **CI/ABL: Yes.**	Hospital patients; stage III/IV advanced cancer; China.	IG: Naikan and Morita therapies. CG: Standard medical care.
Huang et al. (2019); Huang et al. (2021)	Magnanimous therapy • **CI/ABL: Yes.**	Oncology inpatient patients; stage III/IV lung cancer.	IG 1: Group computer magnanimous therapy. IG 2: Individual computer magnanimous therapy. CG: Oncotherapy and usual care.
Julião et al. (2013); Julião et al. (2014); Julião et al. (2017)^c^	Dignity therapy • **CI/ABL: No.**	Inpatient palliative medicine patients; terminally ill; Portugal.	IG: Dignity therapy and a generativity document. CG: Standard palliative care.
Kim et al. (2018)	ILOVEBREAST mobile game • **CI/ABL: No.**	Hospital patients; metastatic breast cancer; South Korea.	IG: ILOVEBREAST self-management mobile game to improve chemotherapy side effects. CG: Routine care and a brochure on the coping strategies for chemotherapy side effects.
Li et al. (2019)^c^	Wellness education • **CI/ABL: No.**	Patients and their caregivers; stage III/IV non-small cell lung cancer; China.	IG: Wellness education. CG: Usual care.
Liao et al. (2013)	Chinese Medicine five-element music • **CI/ABL: Yes.**	Hospital patients; advanced cancer; China.	IG 1: Chinese medicine five-element music composed by professor Shi Feng. IG 2: Western music written by Chris Rea. CG: Usual care.
Maungtoug et al. (2021)^c^	Ritualized chanting in palliative care • **CI/ABL: Yes.**	Thai Buddhist patients; end-of-life cancer; Thailand.	IG: Ritualized chanting in palliative care. CG: Palliative care.
Molassiotis et al. (2021)	Patient- and family-centered psychosocial-based nutrition intervention PIcNIC & PiCNIC2 • **CI/ABL: Yes.**	Ambulatory patients; stage III/IV cancer; Australia and Hong Kong.	IG: Family-centered psychosocial-based nutrition intervention PiCNIC2. CG: Usual care.
Onyechi et al. (2016)^c^	Rational emotive hospice care therapy **• CI/ABL: No.**	Patients and family caregivers; terminal stage breast, cervical, or prostate. cancers; Nigeria	IG: Rational emotive hospice care therapy. CG: Usual care and conventional counseling.
Park et al. (2020)	Lifestyle intervention **• CI/ABL: No.**	Hospital patients; advanced prostate cancer; South Korea.	IG: Lifestyle intervention. CG: Physical activity guidelines.
Patel et al. (2020)^c^	Lay health workers educate engage and encourage patients to share (LEAPS) cancer care **• CI/ABL: Yes.**	Low-income and minority hour-wage workers cancer patients; 23% stage III, 56% stage IV; Atlantic City, New Jersey and Chicago, Illinois.	IG: LEAPS intervention, including advance care planning, symptom assessment and management, and community care delivery. CG: Did not specify control group conditions.
Quílez-Bielsa et al. (2022)^c^	Meaning-centered psychotherapy-essential care **• CI/ABL: No.**	Oncology hospital patients; advanced cancer; Spain.	IG 1: Individual MCP-essential care intervention. IG 2: Group MCP-essential care intervention. CG: Usual care via individual supportive psychological intervention.
Teo et al. (2019)	Cognitive behavioral therapy combined with acceptance and commitment therapy principles **• CI/ABL: Yes.**	Outpatient oncology patients; stage IV breast cancer; USA and Singapore.	IG: Cognitive behavioral therapy symptom management intervention combined with acceptance and commitment therapy mindfulness and values-guided principles. CG: Waitlist control.
Teo et al. (2020)	Cognitive behavioral therapy-based intervention **• CI/ABL: Yes.**	Patients from a national cancer center; stage IV colorectal cancer; Singapore.	IG: Cognitive behavioral therapy-based intervention. CG: Waitlisted control.
Xiao et al. (2013)	Life review program **• CI/ABL: Yes.**	Home-based hospice patients; advanced cancer; advanced cancer; China.	IG: Routine care plus the life review program. CG: Routine care through home visits and weekly telephone follow-up.
Xiao et al. (2022)	Family-oriented dignity therapy **• CI/ABL: Yes.**	Hospital respiratory medicine ward patients; 88% stage III/IV lung cancer; China.	IG: Family-oriented dignity therapy. CG: Attention contacts and usual care.
Yanez et al. (2015)^c^	Cognitive behavioral stress management **• CI/ABL: Yes.**	Men from a comprehensive cancer center and a medical center; stage III/IV prostate cancer; USA.	IG: Cognitive behavioral stress management intervention. CG: Weekly group-based and manualized health information and promotion sessions delivered via a group facilitator that did not include any cognitive behavioral stress management techniques.
Ye et al. (2017)^c^	Be Resilient to Breast Cancer program **• CI/ABL: Yes.**	Women from hospitals; metastatic breast cancer; China.	IG: Be Resilient to Breast Cancer program. CG: CD containing relaxation therapy and monthly telephone follow-up to prevent demoralization.
Zheng et al. (2022)^c^	Very important person (VIP) for future care **• CI/ABL: Yes.**	Hospital oncology patients; stage III/IV cancer; China.	IG: “VIP for future care” in addition to routine disease care and health education. Received symptom management, emotional management, and late-life manuals. CG: Routine disease care and health education. Received symptom management, emotional management, and late-life manuals
***Quasi-experimental studies***			
Catania et al. (2021)	INtervention FOcused on quality of life assessment (INFO-QoL) **• CI/ABL: No.**	Inpatient hospice patients; advanced disease cancer; Italy.	IG: Hospice interdisciplinary team received an educational program for nurse-led INFO-QoL. Patients and their families received INFO-QoL intervention. CG: Usual care. Hospice interdisciplinary team members did not receive the educational program.
Chimluang et al. (2017)^c^	Intervention based on basic Buddhist principles **• CI/ABL: Yes.**	Thai Buddhist patients; terminal cancer; Thailand.	IG: Intervention based on basic Buddhist principles, including precept training, concentration training, and wisdom training. CG: Conventional care. Received advice and Thai herbal medicine and care. Engaged in complementary care activities, merit-making, chanting and practicing the dharma every morning and evening.
Ichihara et al. (2019)	Spiritual care using Spiritual Pain Assessment Sheet (SpiPas) **• CI/ABL: No.**	Hematology and oncology ward and palliative care patients; incurable advanced cancer; Japan.	IG: Spiritual care using SpiPas in addition to usual care. CG: Usual care.
Landa-Ramírez et al. (2020)^c^	Cognitive behavioral therapy **• CI/ABL: Yes.**	Patients at homes; terminal cancer; Mexico.	IG: Culturally adapted cognitive behavioral therapy. CG: N/A (all participants received intervention).
Lee et al. (2017)	Mindfulness-based stress reduction program **• CI/ABL: No.**	Breast cancer clinic patients; metastatic breast cancer; Korea.	IG: Mindfulness-based stress reduction program and practice at home. CG: Weekly phone calls from an oncology nurse specialist to check the adverse effect of anticancer treatment.
Li et al. (2020)^c^	Dignity therapy **• CI/ABL: No.**	End-of-life cancer patients; Taiwan.	IG: Dignity therapy alone or with family members. CG: General visits.
Patel et al. (2021)^c^	Lay health workers educate engage and encourage patients to share (LEAPS) cancer care **• CI/ABL: Yes.**	Low-income and minority hour-wage workers cancer patients; 72% stage III/IV cancer; Atlantic City, New Jersey and Chicago, Illinois.	IG: LEAPS intervention, including advance care planning, symptom assessment and management, and community care delivery. CG: Did not specify control group conditions.
Zhang et al. (2019)	WeChat-based life review program **• CI/ABL: Yes.**	Hospital oncology patients; stage III/IV cancer; China.	IG: WeChat-based life review program and usual care. CG: Usual care.
***Single-arm trials***			
Ando et al. (2008)	Short-term life review **• CI/ABL: No.**	Hospital palliative care patients; terminally ill cancer; Japan.	IG: Short-term life review. CG: N/A (all participants received intervention).
Ando et al. (2016)	Mindfulness art therapy **• CI/ABL: No.**	Hospital female patients; metastatic/stage IV cancer; Japan.	IG: Mindfulness art therapy. CG: N/A (all participants received intervention).
Delrieu et al. (2020)	Physical activity program **• CI/ABL: No.**	Women patients; metastatic breast cancer; France.	IG: Home-based, unsupervised, personalized physical activity program. CG: N/A (all participants received intervention).
Houmann et al. (2014)^**c**^	Dignity therapy **• CI/ABL: Yes.**	Hospitalized (longer than 1 week), outpatients, and homecare patients; incurable cancer; Denmark.	IG: Dignity therapy and a generativity document. CG: N/A (all participants received intervention).
Kang et al. (2015)	Meaning of My Life **• CI/ABL: No.**	Oncology inpatient young adult patients; grade II/III osteosarcoma; South Korea.	IG: “Meaning of My Life” program. CG: N/A (all participants received intervention).
Li et al. (2015)	Caring for couples coping with cancer “(4Cs)” program **• CI/ABL: No.**	Oncology hospital patients and their spouse as primary caregiver spouse; advanced cancer; China.	IG: Caring for couples coping with cancer “(4Cs)” program. CG: N/A (all participants received intervention).
Nakayama et al. (2009)^**c**^	Music therapy **• CI/ABL: Yes.**	Inpatient hospice patients; terminal cancer; Japan.	IG: Group music therapy. CG: N/A (all participants received intervention).
Niki et al. (2019)	Virtual reality **• CI/ABL: No.**	Palliative care wards patients; terminal cancer; Japan.	IG: Virtual reality travel to places participants wanted to go. CG: N/A (all participants received intervention).
Ramos et al. (2018)^c^	Life program **• CI/ABL: Yes.**	Palliative care veteran patients; life-limiting illness; USA.	IG: Life program. CG: N/A (all participants received intervention).
Sakaguchi et al. (2015)^**c**^	Collage activity based on life review **• CI/ABL: No.**	Home-visit nursing care patients, hospital palliative care patients, and home-based palliative care patients visiting a clinic; Japan.	IG: Collage activity based on a life review. CG: N/A (all participants received intervention).
Warth et al. (2018)	Song of Life **• CI/ABL: Yes.**	Palliative care patients; terminally ill cancer; Germany.	IG: Song of Life intervention. CG: N/A (all participants received intervention).
***Feasibility/Acceptability trials***			
Hanson et al. (2013)	Circles of care **• CI/ABL: Yes.**	African American lay health advisors and patients; serious illness; 40% advanced cancer; USA.	IG: Trained lay health advisors to communicate with, advise, and support patients. Trained volunteers with team member or leader training to create and lead a team within their church or community. Patients received Circles of Care intervention. CG: N/A (all participants received intervention).
Molassiotis et al. (2018)	Patient- and family-centered psychosocial-based nutrition intervention PIcNIC & PiCNIC2 **• CI/ABL: Yes.**	Oncology ward patients; advanced cancer; Australia. Patients at home; advanced cancer; Hong Kong.	IG: Family-centered psychosocial-based nutrition intervention PIcNIC. CG: N/A (all participants received intervention).
***Protocols***			
Costas-Muñiz et al., ongoing study	Meaning-centered psychotherapy for Latinos **• CI/ABL: Yes.**	Healthcare providers and Spanish-speaking Latino/a patients; stage III/IV solid tumor cancer; USA.	IG: MCP for Latinos. CG: Waitlisted to receive the intervention approximately 3 months after allocated to the control.
Matthys et al. (2021)	Face-to-face FOCUS + & Web-based iFOCUS **• CI/ABL: No.**	Patients and their primary family caregivers; advanced solid organ cancer (except brain cancer); Belgium, Denmark, Ireland, Italy, the Netherlands, and United Kingdom.	IG 1: Nurse-delivered face-to-face FOCUS + intervention. IG 2: Nurse-delivered web-based iFOCUS intervention. CG: Standard care as usual.
Miyamoto et al. 2022	Managing Cancer and Living Meaningfully (CALM) **• CI/ABL: Yes.**	Hospital respiratory medicine, outpatient psycho-oncology, hospital psycho-oncology, and hospital neuropsychiatry patients; advanced/metastatic solid tumor; Japan.	IG: Managing Cancer and Living Meaningfully intervention. CG: N/A (all participants received intervention).
Scheffold et al. (2015)	Managing Cancer and Living Meaningfully (CALM) **• CI/ABL: No.**	Outpatient patients; stage III/IV malignant solid tumors; Germany.	IG: Brief, individual, manualized CALM intervention. CG: Usual, individual, non-manualized supportive psycho-oncological intervention.
Torres-Blasco et al. (2022)	Caregiver–patients support to Latinx coping advanced cancer (CASA) **• CI/ABL: Yes.**	Oncology Latinx patients and their caregivers; advanced cancer; USA.	N/A. Study is a protocol for intervention.
van der Wel et al. (2022)	Family, outlook, coping, uncertainty, symptom management (FOCUS +) **• CI/ABL: Yes.**	N/A; advanced cancer; Belgium, Denmark, Ireland, Italy, the Netherlands, and UK (Northern Ireland and England).	N/A. No IG & CG. Study described a translation and adaptation process of intervention.
Zhang et al. (2018)	WeChat-based life review program **• CI/ABL: Yes.**	Hospital oncology patients; stage III/IV cancer; China.	IG: WeChat-based life review program and usual care. CG: Usual care.

^a^Coding was completed according to the authors’ written description of cultural consideration efforts

^b^Included in meta-analysis

^c^Excluded from meta-analysis due to insufficient data

There were 48 reports originating from non-WEIRD countries (46 from Asian countries, one from Nigeria, and one from Puerto Rico). Of these studies, 28 utilized interventions developed within WEIRD cultural societies. Over half of those studies (*n* = 16) adapted the intervention beyond language to incorporate local cultural elements, while all others only modified the language in which the intervention was delivered. Across all included studies, 44 interventions described additional cultural modifications compared to 28 interventions focused solely on language match. Majority of interventions were top-down (*n* = 61) with 11 interventions using a bottom-up approach. Bottom-up studies include interventions such as combined Naikan and Morita therapies, which are grounded in Buddhism and Eastern psychology [46].

The most common surface-component adaptations to match the target population were (1) language (e.g., written materials in native language, bilingual speakers; top 10 languages used were Chinese, English, Spanish, Japanese, Italian, Danish, Korean, Dutch, Hindi, and Portuguese); (2) delivery (e.g., face-to-face vs. telephone calls, WeChat); and (3) culturally relevant content (e.g., acupressure and tai chi in traditional Chinese medicine, music). The most prevalent examples of deep structure adaptations of culture were (1) highlighting the importance of family inclusion in caregiving and decision-making (filial piety, familismo); (2) the inclusion of Asian values and cultural norms of Eastern philosophies (e.g., Confucianism, yin and yang) and religions (e.g., Buddhism, Taoism), such as avoiding taboo topics (e.g., death) or concealing a terminal diagnosis; and (3) incorporating Hispanic/Latino^5^ values of establishing trust (confianza), personal relationships (personalismo), and about death and religion (fatalismo/spiritualismo).

### Qualitative Synthesis Results

**Fig. 2:**
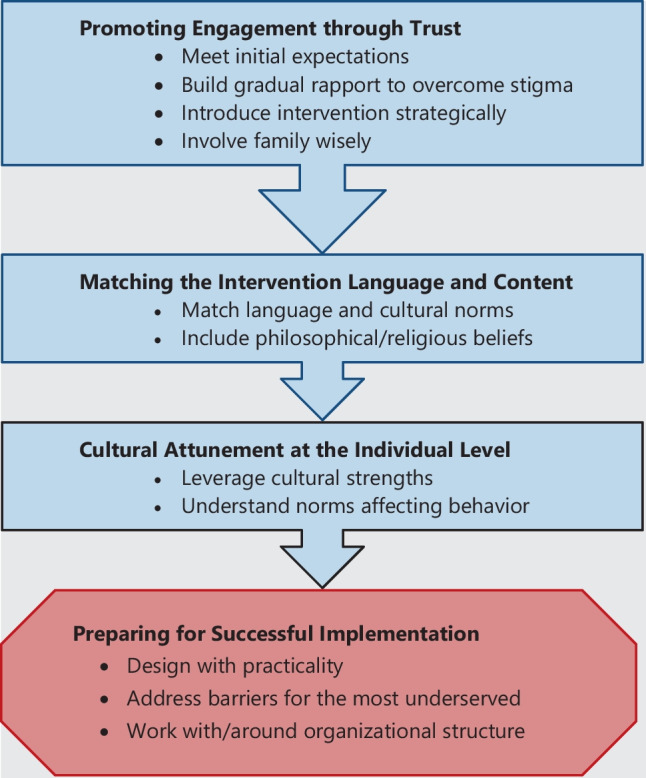
Qualitative synthesis themes

Eleven descriptive subthemes formed four overarching themes about how to incorporate culture in psychosocial interventions for advanced and metastatic cancer starting from introduction of the intervention to long-term implementation: (1) promoting engagement through trust; (2) matching the intervention language and content; (3) cultural attunement at the individual level; and (4) preparing for successful implementation (Fig. 2). Themes remained the same (i.e., low risk of bias) without three lower quality studies from sensitivity analysis (Supplementary Appendix B).

#### Promoting Engagement Through Trust

**Fig. 3:**
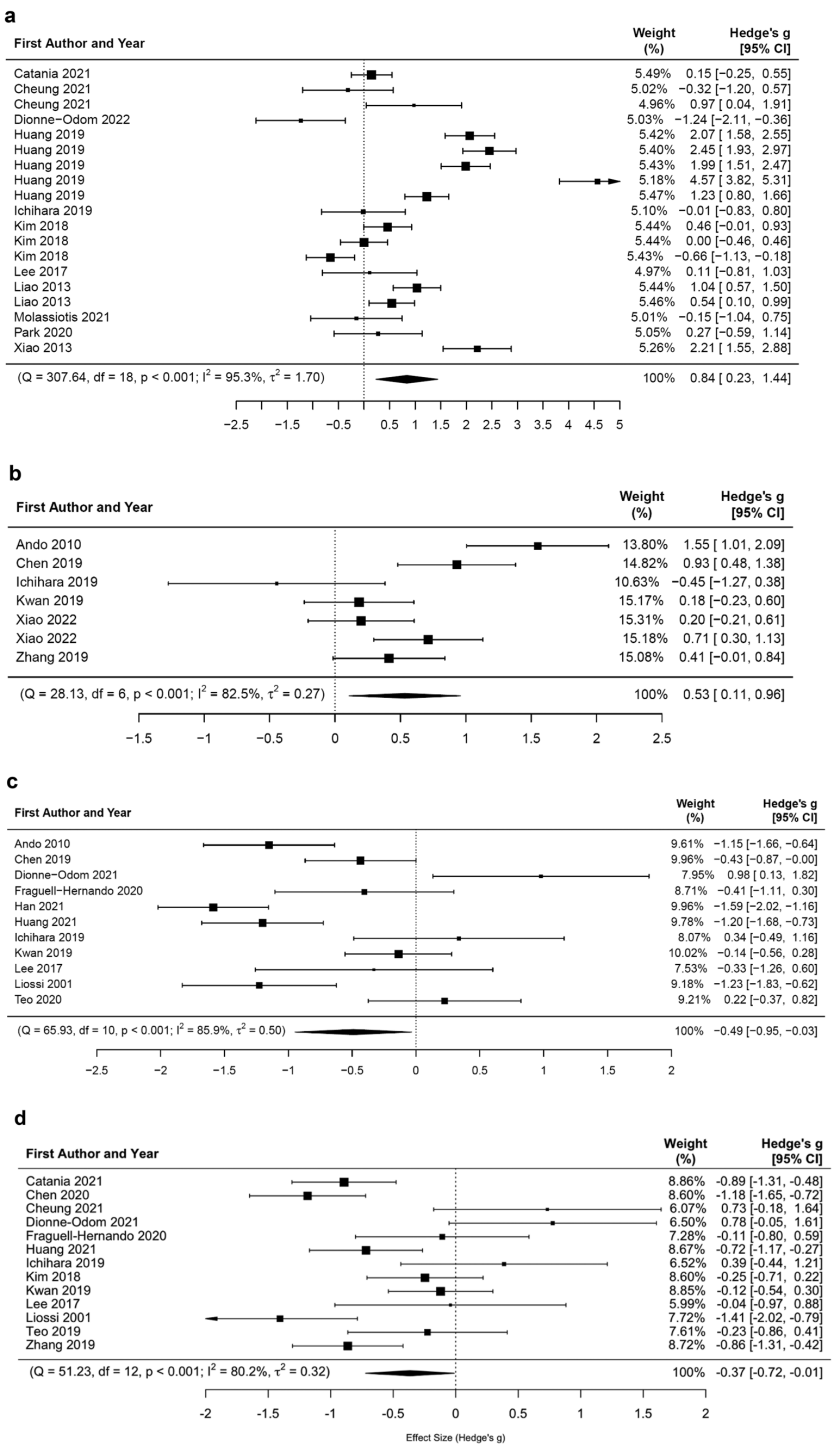
**a** Forest plot of RCT effect sizes (QOL). **b** Forest plot of RCT effect sizes (eudaimonic well-being). **c** Forest plot of RCT effect sizes (distress). **d** Forest plot of RCT effect sizes (anxiety)

To introduce psychosocial interventions for advanced and metastatic cancer populations less aware of psychological distress or need, a basic foundation of trust for both the intervention and the interventionist must first be established. People more easily overcame stigma related to their cancer diagnoses and difficult topics when building rapport with their interventionist face-to-face (for Hispanic/Latino and US Chinese, Taiwanese, Singaporean patients) (Fig. 3).

After trust had been built, the intervention could be introduced and preferably in a structured manner. Focusing on solving physical symptoms earlier in the intervention allowed for further discussions of additional mental health support, especially in Asian populations where the expected outcome may be reducing symptom burden. A trusted figure is recommended for discussing mental health-related topics, such as a nurse in the Singaporean context. Once participants agree to engage in an intervention, studies suggest strategically timing the introduction of taboo topics to maintain rapport:

If you have not build rapport with the patient yet and they are just coming to terms with their diagnosis I think advanced care planning may be just a bit too much to handle cause they don't know where they are headed now… later on maybe after third or fifth follow-up…then it's fine to bring it up. [Healthcare Worker, Singapore] [48]

Family or caregiver involvement was seen as a positive method to increase receptivity across many cultures, but even in family-centric cultures, family involvement could be a barrier for patients to participate or be able to freely express their experience with cancer. In a life review program, a participant described how having a family member present influenced how much they wanted to share:

My daughter’s presence influenced our talks to a degree. I didn’t want to talk about the difficulties of my life in front of her. These experiences would make her feel sorry for me. [Participant, Hong Kong] [49]

#### Matching the Intervention Language and Content

When it came to the core components of the intervention itself, there were suggestions across all studies to adjust the wording and content to match the recipient’s cultural norms and beliefs. For some interventions for Asian populations, this meant couching the intervention content in philosophical traditions and religious beliefs (Confucianism (filial piety and social roles), Taoism, Buddhism, Catholicism). In a meaning-centered psychotherapy intervention for Chinese in the USA, infusing Confucian virtues like the respect for parents and elders was critical:

They have this sense of duty to take care of their parents and to respect them. If you don’t emphasize those values, it will be very hard for [MCP] to be culturally relevant and to get buy-in. [Community Leader, U.S. Chinese] [50]

Revising the wording not only refers to the language of the intervention but presenting less familiar concepts in a manner that matches the participant’s cultural context. In a dignity intervention for Danish patients, participants had difficulty understanding terms for dignity and personal success due to the unacceptable nature of self-praise within their culture [51]. More culturally acceptable phrasing was needed to discuss meaning, end-of-life, or death.

#### Cultural Attunement at the Individual Level

Beyond direct efforts to adjust the language and content of the intervention itself, studies described the importance for interventionists to be broadly aware of cultural group beliefs in order to be attuned with individual-level variations in how much cultural norms affect cognitive and behavioral responses during the intervention. For example, studies focusing on interventions for Asian populations commented on the cultural value of not burdening family members. By understanding the pervasiveness of this cultural value and assessing its importance to each recipient, interventionists may recognize cases where less expressivity does not equal a lack of supportive needs:

Chinese cancer patients…just bear the burden…on their own. They don’t want to create trouble for other people. But in this process, they bury things in their heart, and it’s not good for their health or for their family. [Participant, US Chinese] [52]

Cultural norms also suggest how individuals interact with authority figures, such as a doctor or the interventionist, especially for Asian or Hispanic/Latino cultural groups’ norms of when to engage respectfully or what topics to bring up; the degree to which this affects the intervention should be assessed on an individual basis. Similarly, culturally sensitive topics, such as sexuality in Singapore, could occur earlier for younger patients if it is relevant during interactions. Additionally, interventionists may need to tailor for each family. For instance, in Singaporean culture, concealing the gravity of diagnosis from the patient is normative for preventing worry [48]. Understanding how much the participant actually knows of their diagnosis and their family wishes will help the interventionist tailor the intervention.

Interventionists can also leverage upon the recipient’s cultural contexts to further enhance the effect of the intervention. For interventions in immigrant communities, this may mean being prepared to acknowledge each participant’s unique immigration history and discuss it in a strength-based manner. For example, in a meaning-centered psychotherapy intervention, interventionists were advised to be aware of patients’ immigration histories to reframe attitudes toward end of life:

It’s a survivor culture. Chinese participants have gone through lots of wars, lost lots of things. We try to frame [end-of-life preparation] as hoping for the best, planning for the worst. [Interviewee, US Chinese] [50]

#### Preparing for Successful Implementation

The authors described what they observed or thought was important for interventions to be feasible and better implemented in the future. Practical aspects of working with people with advanced and metastatic cancer were described, such as not always being able to complete the full intervention as intended due to its length, physical fatigue, or the impracticality of a more intensive intervention. Caregivers or family members were often not able to afford the time to provide reliable transportation for the patient to fully participate. In other cases where interventions were digitally adapted, such as using an electronic tablet to deliver a palliative care patient navigator and counseling intervention, some Latino/a participants did not have access to technology or did not know how to use the technology [53].

Overall, the authors described a need to design interventions that are more accessible and flexible to those most underserved and facing more barriers (limited language proficiency, lower education/literacy, lower health awareness, limited finances/insurance) to retain participation. Suggestions included keeping the duration of the intervention more flexible and making adjustments to the method of intervention delivery such as providing video conferencing, telephone intervention, or home-based activities.

The authors also mentioned that some of their interventions lacked the full support needed from their organizational structure (most often in healthcare) and that larger-scale implementation may not be possible. Many healthcare professionals, including nurses, who were already limited in their time with patients found it difficult to also deliver an intervention:

In fact, our routine workload is already very busy and I (now) have to spend more time to do this [advance care planning]. I am actually in the dilemma of how long I should engage in the dialogue while at the same time I have to complete all my other daily assignments. [Hospice Nurse, China] [54]

This suggested that healthcare setting policies and care structures need to change to benefit advanced and metastatic cancer patients; leveraging existing healthcare workers may not be the best solution within current systems.

### Meta-analysis Results

Of the 86 reports included in the review, 19 RCTs, 4 quasi-experimental studies, and 10 single-arm trials reported data sufficient for meta-analysis. Table 3 parts a and b present mean effect sizes for the intervention outcomes. Outcomes showing improvement compared to the control condition were QOL (*g* = 0.84) or the “subjective measurement of an individual’s sense of well-being and ability to enjoy life” [55], eudaimonic well-being (*g* = 0.53) or the positive experience of living one’s life with a sense of meaning and to one’s fullest potential [56], distress (*g* = −0.49), and anxiety (*g* = −0.37). When examining RCT effect sizes between studies with only a language adaptation vs. those evidencing more cultural adaptations, the latter contributed to significantly better outcomes compared to controls only for QOL. Similarly, studies incorporating both surface and deep structure cultural incorporation vs. only surface structure resulted in significantly better outcomes compared to controls only for QOL (Table 3 part c). For the single-arm trials, pre-to-post-intervention, significant reductions occurred for anxiety, physical symptoms, and depression. Only one single-arm study was identified having a medium risk of bias (Supplementary Appendix B); however, all studies with available data were included as recommended [44]. Egger’s test results (Table 3 part a) point to some risk of publication bias; however, publication bias is less likely because small studies generally contributed to lowering meta-analysis effect size compared to larger studies, or there was no significant effect [57, 58]. See Supplementary Appendix F for additional forest plots and list of studies.

**Table 3:** Meta-analysis effect sizes by psychosocial outcome

**(a) RCTs and quasi-experimental studies**											
**Outcome**	***n***	***k***	***g***	**95% CI**	***p***		***Q***			***I***^**2**^	**Egger’s test**
QOL	11	19	0.84	0.23, 1.44	0.007		(df = 18) = 307.64, *p* < .0001			95.35%	*b* = 1.67, *p* = 0.407
Physical symptoms	5	10	0.32	−0.26, 0.91	0.279		(df = 9) = 50.93, *p* < .0001			87.74%	*N/A, less than 10 studies*
Eudaimonic well-being	6	7	0.53	0.11, 0.96	0.014		(df = 6) = 28.13, *p* < .0001			82.46%	*N/A, less than 10 studies*
Distress	11	11	−0.49	−0.95, −0.03	0.035		(df = 10) = 65.93, *p* < .0001			85.95%	*b* = −2.00, *p* = 0.036
Anxiety	13	13	−0.37	−0.72, −0.01	0.043		(df = 12) = 51.23, *p* < .0001			80.20%	*b* = −1.80, *p* < 0.003
Depression	15	15	−0.39	−0.86, 0.07	0.097		(df = 14) = 88.20, *p* < .001			89.91%	*b* = −2.09, *p* = 0.002

**(b) Single-arm trials**											
**Outcome**	***n***	***k***	***g***	**95% CI**	***p***		***Q***			***I***^**2**^	
QOL	4	5	0.56	−0.09, 1.21	0.094		(df = 4) = 11.91, *p* = 0.018			81.37%	
Physical symptoms	5	10	−0.39	−0.56, −0.21	<.0001		(df = 9) = 3.00, *p* = 0.964			0.12%	
Anxiety	6	6	−0.54	−0.87, −0.21	0.001		(df = 5) = 9.68, *p* = 0.085			47.64%	
Depression	6	6	−0.38	−0.74, −0.01	0.042		(df = 5) = 11.87, *p* = 0.037			57.98%	

**(c) RCT moderator analysis for each outcome**											
**Outcome**	**Cultural incorporation**				***n***	***k***	***g***	**95% CI**		***p***	
QOL	Only language				4	6	0.01	−0.98, 0.99		0.045	
	Additional incorporations				7	13	1.23	0.55, 1.91			
QOL	Surface structure only				6	10	−0.03	−0.65, 0.60		<.0001	
	Surface & deep structure				4	9	1.77	1.13, 2.42			
Distress	Only language				6	6	−0.45	−1.11, 0.22		0.882	
	Additional incorporations				5	5	−0.53	−1.23, 0.17			
Distress	Surface structure only				7	7	−0.32	−0.90, 0.28		0.349	
	Surface & deep structure				4	4	−0.77	−1.51, −0.03			
Anxiety	Only language				7	7	−0.55	−1.04, 0.07		0.269	
	Additional incorporations				6	6	−0.15	−0.67, 0.37			
Depression	Only language				7	7	−0.67	−1.35, 0.02		0.281	
	Additional incorporations				8	8	−0.15	−0.80 0.50			

## Discussion

This systematic review synthesized the literature on psychosocial and behavioral interventions in advanced and metastatic cancer that incorporate culture, particularly for understudied and underserved populations. Various approaches for incorporating culture into interventions were identified, in addition to the gaps and opportunities for psychosocial and behavioral intervention science in advanced and metastatic cancer. Similar to prior reviews reporting the benefits of psychosocial or behavioral interventions in advanced and metastatic cancer [59], this systematic review demonstrates the overall benefit of psychosocial interventions that incorporated the culture of their targeted intervention population, particularly for QOL. The present review bolsters what is known by extensively documenting the forms of cultural incorporation in psychosocial and behavioral interventions in advanced and metastatic cancer and provides directions for moving forward.

Past reviews in cancer-related psychosocial or behavioral interventions were limited to specific types of interventions and outcomes and/or were limited to RCTs. Due to the importance of this work and how understudied these populations are, the present review applied inclusive methods to identify studies that might otherwise have been missed. First, the definition of psychosocial or behavioral interventions was broadened to include “nonmedical interventions intended to modify psychological, social, and behavioral processes, including cognition and emotions” [59] (e.g., complementary alternative medicine, rehabilitation medicine, native/ethnic traditions-based). A similar approach was applied in the conceptualization of the population of interest with the goal of including advanced and metastatic cancer populations globally and attending to research on medically underserved and understudied groups (e.g., ethnoracial minorities, younger/AYA, rural in WEIRD countries) [32]. Although comprehensive, a consequence of an inclusive search strategy is the high level of heterogeneity across the included studies. As such, substantial caution is needed when interpreting the present meta-analytic results. For example, heterogeneity across studies potentially shrouds notable effects that may be found in future reviews. This can be demonstrated via sensitivity analysis in our meta-analysis for depression, where exclusion of a tai chi study [60] results in a significant effect size between control and intervention conditions because most other studies were psychosocial or spiritual in nature that likely shift more proximal mechanisms of depression. Further, the available data is likely underpowered to detect moderation effects across psychosocial outcomes. Nonetheless, cultural modifications beyond language match appeared beneficial for QOL. Although heterogeneity of studies and underpowered data limit our quantitative findings, there is still much to learn from examining the body of work conducted thus far on this topic.

To the authors’ knowledge, no other systematic reviews have coded for cultural consideration involving understudied/underserved groups and international populations in cancer survivorship, with theories/models, methods, and components of adaptation/tailoring outlined. This work is a significant step forward in this regard and provides a variety of possibilities for future research. One direction that could greatly impact the field moving forward is clarity in modifications in design for cultural fit. In conceptualization of this review, the author group intended to summarize “systematic modification[s] of a protocol to consider language, culture, and context in such a way that it is compatible with the client’s cultural patterns, meanings, and values” [61]. However, in conducting this review, the author group found the included reports often contained little detail on what cultural incorporations were made systematically.

Another opportunity for further growth is the inclusion of deep-level cultural congruence in intervention development. A preponderance of studies included in this review focused adaptation efforts on language, to translate intervention materials for the target population. However, studies that demonstrated cultural incorporation beyond surface structure (i.e., language) such as addressing historical events when conducting a life review with Chinese chemotherapy patients [62] appeared more beneficial for QOL compared to interventions describing a language consideration only. Moving forward, a greater focus on deep culture structure is needed. Evidence from psychology suggests consideration of deep cultural structure need not be arduous. For example, a study examining the effect of relational savoring in parenting suggests a greater intervention engagement for Latina mothers due to the compatibility of relational savoring with cultural values of familism and simpatia [63]. Although not originally designed specifically for Latina mothers, deep structure cultural fit allowed for positive parental outcomes such as increased parental pride, gratitude, and feelings of closeness with their children.

The utility of applying deep cultural structure in intervention work aligns with most theoretical models of cultural adaptation; however, only 8 of the 86 reports utilized a model/framework for cultural incorporation, limiting the ability to evaluate the usefulness of these tools. Future studies may consider use of such models/frameworks in intervention development. This review initially intended to code mentions of the (1) fidelity of the intervention, (2) rationale for tailoring or adapting an intervention, and (3) future considerations for dissemination/implementation. However, these details were dropped from extraction because they were sparse and unavailable. As such, the results in this review are limited to details available in the final dataset of reports and past published work referenced in those reports. Scarce data also limited opportunities to examine non-self-report biobehavioral outcomes such as performance status in activities of daily living (*n* = 4), actigraphy-measured physical activity (*n* = 3), actigraphy-measured sleep (*n* = 2), heart rate (*n* = 2), BMI (*n* = 1), and tumor progression/survival (*n* = 1). Only 2 studies examined biomarkers related to immune function and inflammation. The potential impact of interventions with cultural incorporation on biobehavioral outcomes is ripe for future investigation.

A limitation of this review is that articles could only be included if they were available in English. Although effort was made to obtain English-translated versions of non-English publications from authors, seven reports were ultimately excluded from the present review. It is also important to note that for non-US research, a greater number of studies were conducted in Asia compared to other regions such as Africa and South America. It is unclear whether this is due to the scarcity of research on this topic in those regions or if research reports are in non-English outlets inaccessible via the databases searched in this review. Nonetheless, future work incorporating culture in psychosocial or behavioral interventions advanced and metastatic cancer can benefit from the more expansive global perspective sought out by this review.

Additionally, important cultural considerations in the reports may have been missed because the coding process relied on explicit written description. Much of the cultural incorporation may occur in unscripted interactions between interventionists and recipients. Future research should report such unscripted cultural incorporation; this will facilitate a stronger understanding of the importance of cultural congruence in intervention work. Additionally, more details such as cultural adaptation rationale as well as whether culture was considered earlier in the design for dissemination/implementation will be beneficial.

Despite the limitations of this review, the qualitative findings also highlight important future directions for incorporating culture in psychosocial and behavioral interventions for advanced and metastatic cancer patients. Notably, the qualitative themes identified in this review suggest cultural consideration should permeate the multiple stages of intervention development. Further, there is a clear opportunity for understudied/underserved populations to be involved in the research. To broaden the availability of culturally appropriate psychosocial interventions for advanced and metastatic cancer survivors, it is necessary to gain an understanding of the varying manifestations of culture, particularly beyond stereotypical values often associated with different ethnic/racial minorities (i.e., collectivism, familism). This can be achieved through community-based participatory research conducted among underserved subgroups of advanced and metastatic cancer survivors, their families/caregivers, and healthcare providers.

Literature on cultural adaptations of interventions demonstrate significant benefits to diverse populations including five times greater odds for symptom remission than unadapted intervention conditions [64], and concerns about fidelity have been debunked as most cultural adaptations maintain core aspects of an intervention [29]. While culturally appropriate interventions expand access, relevance, and engagement of overlooked cancer survivor groups, more investigation is needed on how to best navigate the dichotomy between generalizing and individualizing an intervention. “Dynamic sizing” and flexibility are imperative to prevent overgeneralizations and to increase cultural competence [65, 66]. Further, increasing the personal relevance of interventions may improve outcomes among underserved groups [67].

## Conclusion

Intentional cultural incorporation and adaptation of psychosocial and behavioral interventions are valuable for addressing the survivorship needs of underserved and understudied populations with advanced and metastatic cancer. Thoughtful consideration of deep cultural structures influencing the population of focus and the use of cultural adaptation models and frameworks can improve the impact of future interventions. This work will ensure minoritized groups are not "left behind" as long-term survivorship needs come to be universally addressed.

## Supplementary Information

Below is the link to the electronic supplementary material.

Supplementary file1 (DOCX 568 KB)

## Data Availability

The data extracted from included studies, data used for all analyses, analytic code, and any other materials used in the review are available upon request.

## Footnotes

1 “Culture” was not a MeSH term; thus, culture-related free-text keywords were utilized for review feasibility. Full search strategy can be found in Supplementary Appendix A.

2 Six protocols were included in the review but did not have participant data.

3 Only 64 reports in this review included valid cancer survivor age data for analysis.

4 Out of 7 total reports with healthcare provider, lay health worker, or stakeholder data, only 2 reports provided sex data for healthcare providers only.

5 We use Hispanic/Latino because this is how the majority population identifies and less than 3% of this population use other labels [47].

## References

[1] Mollica MA, Smith AW, Tonorezos E, Castro K, Filipski KK, Guida J, et al. Survivorship for individuals living with advanced and metastatic cancers: National Cancer Institute Meeting Report. J Natl Cancer Inst. 2022;114:489–95. 34878107 10.1093/jnci/djab223 PMC9002286

[2] Gallicchio L, Devasia TP, Tonorezos E, Mollica MA, Mariotto A. Estimation of the number of individuals living with metastatic cancer in the United States. J Natl Cancer Inst. 2022;114:1476–83. 35993614 10.1093/jnci/djac158 PMC9949565

[3] Barton-Burke M, Smith E, Frain J, Loggins C. Advanced cancer in underserved populations. Semin Oncol Nurs. 2010;26:157–67. 20656139 10.1016/j.soncn.2010.05.003

[4] Zavala VA, Bracci PM, Carethers JM, Carvajal-Carmona L, Coggins NB, Cruz-Correa MR, et al. Cancer health disparities in racial/ethnic minorities in the United States. Br J Cancer. 2021;124:315–32. 32901135 10.1038/s41416-020-01038-6 PMC7852513

[5] Moghaddam N, Coxon H, Nabarro S, Hardy B, Cox K. Unmet care needs in people living with advanced cancer: a systematic review. Support Care Cancer. 2016;24:3609–22. 27137214 10.1007/s00520-016-3221-3

[6] Hu X, Ma J, Jemal A, Zhao J, Nogueira L, Ji X, et al. Suicide risk among individuals diagnosed with cancer in the US, 2000–2016. JAMA Netw Open. 2023;6:e2251863. 36662522 10.1001/jamanetworkopen.2022.51863 PMC9860529

[7] Ganz PA, Stanton AL. Living with metastatic breast cancer. In: Ganz PA, editor. Improving outcomes for breast cancer survivors: perspectives on research challenges and opportunities. Cham: Springer International Publishing; 2015. p. 243–54.

[8] Committee on Developing Evidence-Based Standards for Psychosocial Interventions for Mental Disorders, Board on Health Sciences Policy, Institute of Medicine. Psychosocial Interventions for mental and substance use disorders: a framework for establishing evidence-based standards. Washington, DC: National Academies Press; 2015. 26203478

[9] Jarnagin JX, Baiev I, Van Seventer EE, Shah Y, Mojtahed A, Allen JN, et al. Changes in patient-reported outcomes (PROs) and tumor markers (TMs) to predict treatment response and survival in patients with metastatic gastrointestinal (GI) cancer. J Clin Orthod. 2021;39:154–154.

[10] Manson SM. The role of culture in effective intervention design, implementation, and research: its universal importance. Prev Sci. 2020;21:93–7. 31659610 10.1007/s11121-019-01065-7 PMC6980655

[11] Kagawa Singer M, Dressler W, George S. NIH Expert Panel. Culture: the missing link in health research. Soc Sci Med. 2016;170:237–46. 27542574 10.1016/j.socscimed.2016.07.015

[12] Kim HS, Lawrie SI. Culture and motivation. In: Cohen D, Kitayama S, editors. Handbook of cultural psychology. 2nd ed. New York, NY: Guilford Press; 2019. p. 268–91.

[13] Dressler WW. Culture and the risk of disease. Br Med Bull. 2004;69:21–31. 15226194 10.1093/bmb/ldh020

[14] Kitayama S, et al. Culture and basic psychological processes--toward a system view of culture: comment on Oyserman et al. (2002). Psychol Bull. 2002;128:89–96. 11843550 10.1037/0033-2909.128.1.89

[15] Costas-Muñiz R, Garduño-Ortega O, Torres-Blasco N, Castro-Figueroa E, Gany F. “Maintaining hope:” challenges in counseling latino patients with advanced cancer. J Psychosoc Oncol Res Pract. 2020;2: e028. 33154993 10.1097/OR9.0000000000000028 PMC7597581

[16] Kreling B, Selsky C, Perret-Gentil M, Huerta EE, Mandelblatt JS, Latin American Cancer Research Coalition. “The worst thing about hospice is that they talk about death”: contrasting hospice decisions and experience among immigrant Central and South American Latinos with US-born White, non-Latino cancer caregivers. Palliat Med. 2010;24:427–34. 20507867 10.1177/0269216310366605 PMC3570252

[17] Updegraff KA, Umaña-Taylor AJ, Rodríguez De Jesús SA, McHale SM, Feinberg MF, Kuo SI-C. Family-focused prevention with Latinos: what about sisters and brothers? J Fam Psychol. 2016;30:633–40. 27077238 10.1037/fam0000200

[18] Benish SG, Quintana S, Wampold BE. Culturally adapted psychotherapy and the legitimacy of myth: a direct-comparison meta-analysis. J Couns Psychol. 2011;58:279–89. 21604860 10.1037/a0023626

[19] Griner D, Smith TB. Culturally adapted mental health intervention: a meta-analytic review. Psychotherapy. 2006;43:531–48. 22122142 10.1037/0033-3204.43.4.531

[20] Chowdhary N, Jotheeswaran AT, Nadkarni A, Hollon SD, King M, Jordans MJD, et al. The methods and outcomes of cultural adaptations of psychological treatments for depressive disorders: a systematic review. Psychol Med. 2014;44:1131–46. 23866176 10.1017/S0033291713001785 PMC3943384

[21] Heim E, Kohrt BA. Cultural Adaptation of scalable psychological interventions: clinical psychology in Europe. 2019;1:1–22.

[22] Bernal G, Sáez-Santiago E. Culturally centered psychosocial interventions. J Community Psychol. 2006;34:121–32. 10.1176/appi.focus.24022022 PMC11571183 39563877

[23] Hwang W-C. The psychotherapy adaptation and modification framework: application to Asian Americans. Am Psychol. 2006;61:702–15. 17032070 10.1037/0003-066X.61.7.702

[24] Domenech Rodríguez MM, Baumann AA, Schwartz AL. Cultural adaptation of an evidence based intervention: from theory to practice in a Latino/a community context. Am J Community Psychol. 2011;47:170–86. 21116707 10.1007/s10464-010-9371-4

[25] Hwang W-C. The formative method for adapting psychotherapy (FMAP): a community-based developmental approach to culturally adapting therapy. Prof Psychol Res Pr. 2009;40:369–77. 20625458 10.1037/a0016240 PMC2898145

[26] Kreuter MW, Lukwago SN, Bucholtz RDDC, Clark EM, Sanders-Thompson V. Achieving cultural appropriateness in health promotion programs: targeted and tailored approaches. Health Educ Behav. 2003;30:133–46. 12693519 10.1177/1090198102251021

[27] Falicov CJ. Commentary: On the wisdom and challenges of culturally attuned treatments for Latinos. Fam Process. 2009;48:292–309. 19579910 10.1111/j.1545-5300.2009.01282.x

[28] Resnicow K, Soler R, Braithwaite RL, Ahluwalia JS, Butler J. Cultural sensitivity in substance use prevention. J Community Psychol. 2000;28:271–90.

[29] Chu J, Leino A. Advancement in the maturing science of cultural adaptations of evidence-based interventions. J Consult Clin Psychol. 2017;85:45–57. 28045287 10.1037/ccp0000145

[30] Henrich J, Heine SJ, Norenzayan A. Most people are not WEIRD. Nature. 2010;466:29. 20595995 10.1038/466029a

[31] Henrich J, Heine SJ, Norenzayan A. Beyond WEIRD: towards a broad-based behavioral science. Behav Brain Sci. 2010;33:111–35.

[32] Muthukrishna M, Bell AV, Henrich J, Curtin CM, Gedranovich A, McInerney J, et al. Beyond Western, educated, industrial, rich, and democratic (WEIRD) psychology: measuring and mapping scales of cultural and psychological distance. Psychol Sci. 2020;31:678–701. 32437234 10.1177/0956797620916782 PMC7357184

[33] Mollica MA, Tesauro G, Tonorezos ES, Jacobsen PB, Smith AW, Gallicchio L. Current state of funded National Institutes of Health grants focused on individuals living with advanced and metastatic cancers: a portfolio analysis. J Cancer Surviv. 2021;15:370–4. 33651327 10.1007/s11764-021-01008-8

[34] Thomas J, Harden A. Methods for the thematic synthesis of qualitative research in systematic reviews. BMC Med Res Methodol. 2008;8:45. 18616818 10.1186/1471-2288-8-45 PMC2478656

[35] Coulman KD, MacKichan F, Blazeby JM, Owen-Smith A. Patient experiences of outcomes of bariatric surgery: a systematic review and qualitative synthesis. Obes Rev. 2017;18:547–59. 28273694 10.1111/obr.12518 PMC5709707

[36] Carroll C, Booth A, Lloyd-Jones M. Should we exclude inadequately reported studies from qualitative systematic reviews? An evaluation of sensitivity analyses in two case study reviews. Qual Health Res. 2012;22:1425–34. 22865107 10.1177/1049732312452937

[37] Carroll C, Booth A. Quality assessment of qualitative evidence for systematic review and synthesis: is it meaningful, and if so, how should it be performed? Res Synth Methods. 2015;6:149–54. 26099483 10.1002/jrsm.1128

[38] Soilemezi D, Linceviciute S. Synthesizing qualitative research. Int J Qual Methods. 2018;17:160940691876801.

[39] Britten N, Pope C. Medicine taking for asthma: a worked example of meta-ethnography. In: Synthesizing Qualitative Research. Chichester, UK: John Wiley & Sons Ltd.; 2012. p. 41–57.

[40] Mens MG, Helgeson VS, Lembersky BC, Baum A, Scheier MF. Randomized psychosocial interventions for breast cancer: impact on life purpose. Psychooncology. 2016;25:618–25. 26123574 10.1002/pon.3891 PMC4945105

[41] West SL, Gartlehner G, Mansfield AJ, et al. Table 7, Summary of common statistical approaches to test for heterogeneity. In: Comparative effectiveness review methods: clinical heterogeneity. Rockville, MD: Agency for Healthcare Research and Quality; 2010. p. 21. 21433337

[42] Sterne JAC, Sutton AJ, Ioannidis JPA, Terrin N, Jones DR, Lau J, et al. Recommendations for examining and interpreting funnel plot asymmetry in meta-analyses of randomised controlled trials. BMJ. 2011;343: d4002. 21784880 10.1136/bmj.d4002

[43] Fu R, Gartlehner G, Grant M, Shamliyan T, Sedrakyan A, Wilt TJ, et al. Conducting quantitative synthesis when comparing medical interventions: AHRQ and the Effective Health Care Program. J Clin Epidemiol. 2011;64:1187–97. 21477993 10.1016/j.jclinepi.2010.08.010

[44] Barker TH, Stone JC, Sears K, Klugar M, Tufanaru C, Leonardi-Bee J, et al. The revised JBI critical appraisal tool for the assessment of risk of bias for randomized controlled trials. JBI Evid Synth. 2023;21:494–506. 36727247 10.11124/JBIES-22-00430

[45] Slim K, Nini E, Forestier D, Kwiatkowski F, Panis Y, Chipponi J. Methodological index for non-randomized studies (minors): development and validation of a new instrument. ANZ J Surg. 2003;73:712–6. 12956787 10.1046/j.1445-2197.2003.02748.x

[46] Kitanishi K, Mori A. Morita therapy: 1919 to 1995. Psychiatry Clin Neurosci. 1995;49:245–54. 8726108 10.1111/j.1440-1819.1995.tb01896.x

[47] Noe-Bustamante L. About one-in-four U.S. Hispanics have heard of Latinx, but just 3% use it. https://www.pewresearch.org/hispanic/2020/08/11/about-one-in-four-u-s-hispanics-have-heard-of-latinx-but-just-3-use-it/. Accessed 19 Nov 2023.

[48] Yang GM, Dionne-Odom JN, Foo YH, Chung AHM, Kamal NHA, Tan L, et al. Adapting ENABLE for patients with advanced cancer and their family caregivers in Singapore: a qualitative formative evaluation. BMC Palliat Care. 2021;20:86. 34158022 10.1186/s12904-021-00799-y PMC8218975

[49] Xiao H, Kwong E, Pang S, Mok E. Perceptions of a life review programme among Chinese patients with advanced cancer. J Clin Nurs. 2012;21:564–72. 21923673 10.1111/j.1365-2702.2011.03842.x

[50] Leng J, Lui F, Chen A, Huang X, Breitbart W, Gany F. Adapting meaning-centered psychotherapy in advanced cancer for the Chinese immigrant population. J Immigr Minor Health. 2018;20:680–6. 28455760 10.1007/s10903-017-0591-7 PMC5660672

[51] Houmann LJ, Rydahl-Hansen S, Chochinov HM, Kristjanson LJ, Groenvold M. Testing the feasibility of the dignity therapy interview: adaptation for the Danish culture. BMC Palliat Care. 2010;9:21. 20860786 10.1186/1472-684X-9-21 PMC2954968

[52] Leng J, Lui F, Huang X, Breitbart W, Gany F. Patient perspectives on adapting meaning-centered psychotherapy in advanced cancer for the Chinese immigrant population. Support Care Cancer. 2019;27:3431–8. 30661201 10.1007/s00520-019-4638-2 PMC6642030

[53] Bekelman DB, Fink RM, Sannes T, Kline DM, Borrayo EA, Turvey C, et al. Puente para cuidar (bridge to caring): a palliative care patient navigator and counseling intervention to improve distress in Latino/as with advanced cancer. Psychooncology. 2020;29:688–95. 31834646 10.1002/pon.5313

[54] Lin C-P, Evans CJ, Koffman J, Chen P-J, Hou M-F, Harding R. Feasibility and acceptability of a culturally adapted advance care planning intervention for people living with advanced cancer and their families: a mixed methods study. Palliat Med. 2020;34:651–66. 32081076 10.1177/0269216320902666

[55] NCI Thesaurus. https://ncit.nci.nih.gov/ncitbrowser/pages/concept_details.jsf?dictionary=NCI_Thesaurus&version=23.10e&code=C17047&ns=ncit&type=properties&key=null&b=1&n=0&vse=null. Accessed 21 Nov 2023.

[56] Niemiec CP. Eudaimonic well-being. In: Michalos AC, editor. Encyclopedia of Quality of Life and Well-Being Research. Dordrecht: Springer, Netherlands; 2014. p. 2004–5.

[57] Teo I, Krishnan A, Lee GL. Psychosocial interventions for advanced cancer patients: a systematic review. Psychooncology. 2019;28:1394–407. 31077475 10.1002/pon.5103

[58] Warth M, Zöller J, Köhler F, Aguilar-Raab C, Kessler J, Ditzen B. Psychosocial interventions for pain management in advanced cancer patients: a systematic review and meta-analysis. Curr Oncol Rep. 2020;22:3. 31965361 10.1007/s11912-020-0870-7 PMC8035102

[59] Grant S, Mayo-Wilson E, Montgomery P, et al. CONSORT-SPI 2018 Explanation and elaboration: guidance for reporting social and psychological intervention trials. Trials. 2018;19:406. 30060763 10.1186/s13063-018-2735-z PMC6066913

[60] Cheung DST, Takemura N, Lam TC, Ho JCM, Deng W, Smith R, et al. Feasibility of aerobic exercise and tai-chi interventions in advanced lung cancer patients: a randomized controlled trial. Integr Cancer Ther. 2021;20:15347354211033352. 34549648 10.1177/15347354211033352 PMC8461121

[61] Bernal G, Jiménez-Chafey MI, Domenech Rodríguez MM. Cultural adaptation of treatments: a resource for considering culture in evidence-based practice. Prof Psychol Res Pr. 2009;40:361–8.

[62] Chen Y, Xiao H, Zheng J, Zhang X, Lin X. Effects of a mind map-based life review programme on psychospiritual well-being in cancer patients undergoing chemotherapy: a randomised controlled trial. Eur J Cancer Care. 2020;29: e13221. 10.1111/ecc.13221 31908102

[63] Borelli JL, Kerr ML, Smiley PA, Rasmussen HF, Hecht HK, Campos B. Relational savoring intervention: positive impacts for mothers and evidence of cultural compatibility for Latinas. Emotion. 2023;23:303–20. 35549365 10.1037/emo0001102

[64] Hall GCN, Ibaraki AY, Huang ER, Marti CN, Stice E. A Meta-analysis of cultural adaptations of psychological interventions. Behav Ther. 2016;47:993–1014. 27993346 10.1016/j.beth.2016.09.005

[65] Sue S. In search of cultural competence in psychotherapy and counseling. Am Psychol. 1998;53:440–8. 9572007 10.1037//0003-066x.53.4.440

[66] Hunt LM. Beyond cultural competence. In: The social medicine reader, vol. 22. 3rd ed. Duke University Press; 2019. p. 127–31.

[67] Hall GCN, Berkman ET, Zane NW, Leong FTL, Hwang W-C, Nezu AM, et al. Reducing mental health disparities by increasing the personal relevance of interventions. Am Psychol. 2021;76:91–103. 32118456 10.1037/amp0000616 PMC8034200

[68] Catania G, Zanini M, Signori A, Dal Molin A, Pilastri P, Bottino M, et al. Providing a nurse-led complex nursing INtervention FOcused on quality of life assessment on advanced cancer patients: the INFO-QoL pilot trial. Eur J Oncol Nurs. 2021;52:101961. 33984605 10.1016/j.ejon.2021.101961

[69] Delrieu L, Pialoux V, Pérol O, Morelle M, Martin A, Friedenreich C, et al. Feasibility and health benefits of an individualized physical activity intervention in women with metastatic breast cancer: intervention study. JMIR Mhealth Uhealth. 2020;8:e12306. 32012082 10.2196/12306 PMC7013652

[70] Han X-B, Fang Y-Q, Liu S-X, Tan Y, Hou J-J, Zhao L-J, et al. Efficacy of combined naikan and morita therapies on psychological distress and posttraumatic growth in Chinese patients with advanced cancer: a randomized controlled trial. Medicine. 2021;100:e26701. 34397698 10.1097/MD.0000000000026701 PMC8322545

[71] Hanson LC, Armstrong TD, Green MA, Hayes M, Peacock S, Elliot-Bynum S, et al. Circles of care: development and initial evaluation of a peer support model for African Americans with advanced cancer. Health Educ Behav. 2013;40:536–43. 23077156 10.1177/1090198112461252 PMC5464604

[72] Houmann LJ, Chochinov HM, Kristjanson LJ, Petersen MA, Groenvold M. A prospective evaluation of Dignity Therapy in advanced cancer patients admitted to palliative care. Palliat Med. 2014;28:448–58. 24311296 10.1177/0269216313514883

[73] Ichihara K, Ouchi S, Okayama S, Kinoshita F, Miyashita M, Morita T, et al. Effectiveness of spiritual care using spiritual pain assessment sheet for advanced cancer patients: a pilot non-randomized controlled trial. Palliat Support Care. 2019;17:46–53. 30683167 10.1017/S1478951518000901

[74] Lin J, Zhao Y, Guo Q. Dignity therapists’ experience of conducting dignity therapy with terminal cancer patients in mainland China: a descriptive qualitative study. Eur J Cancer Care. 2022;31:e13670. 10.1111/ecc.13670 35948415

[75] Maungtoug N, Othaganont P, Liehr P. Adding ritualized chanting to the palliative care of cancer patients at the end of life: a randomized controlled trial. J Soc Work End Life Palliat Care. 2021;17:35–49. 33491604 10.1080/15524256.2021.1871703

[76] Molassiotis A, Roberts S, Cheng HL, To HKF, Ko PS, Lam W, et al. Partnering with families to promote nutrition in cancer care: feasibility and acceptability of the PIcNIC intervention. BMC Palliat Care. 2018;17:50. 29558917 10.1186/s12904-018-0306-4 PMC5859412

[77] Nakayama H, Kikuta F, Takeda H. A pilot study on effectiveness of music therapy in hospice in Japan. J Music Ther. 2009;46:160–72. 19463033 10.1093/jmt/46.2.160

[78] Nunziante F, Tanzi S, Alquati S, Autelitano C, Bedeschi E, Bertocchi E, et al. Providing dignity therapy to patients with advanced cancer: a feasibility study within the setting of a hospital palliative care unit. BMC Palliat Care. 2021;20:129. 34399737 10.1186/s12904-021-00821-3 PMC8369621

[79] Patel M, Andrea N, Jay B, Coker TR. A community-partnered, evidence-based approach to improving cancer care delivery for low-income and minority patients with cancer. J Community Health. 2019;44:912–20. 30825097 10.1007/s10900-019-00632-x PMC7046315

[80] Patel MI, Khateeb S, Coker T. A randomized trial of a multi-level intervention to improve advance care planning and symptom management among low-income and minority employees diagnosed with cancer in outpatient community settings. Contemp Clin Trials. 2020;91:105971. 32145441 10.1016/j.cct.2020.105971 PMC7263972

[81] Patel MI, Khateeb S, Coker T. Association of a lay health worker-led intervention on goals of care, quality of life, and clinical trial participation among low-income and minority adults with cancer. JCO Oncol Pract. 2021;17:e1753–62. 33999691 10.1200/OP.21.00100 PMC9810146

[82] Schulman-Green D, Feder SL, Collett D, Aaron EM, Haron Y, Eilon Y, et al. Adapting a palliative care-focused cancer self- and family management intervention for use in Israel. Int J Palliat Nurs. 2022;28:378–87. 36006792 10.12968/ijpn.2022.28.8.378

[83] Anderson KO, Mendoza TR, Payne R, Valero V, Palos GR, Nazario A, et al. Pain education for underserved minority cancer patients: a randomized controlled trial. J Clin Oncol. 2004;22:4918–25. 15611506 10.1200/JCO.2004.06.115

