# Supplementary material for: Promising Directions: A Systematic Review of Psychosocial and Behavioral Interventions with Cultural Incorporation for Advanced and Metastatic Cancer
Source: Int J Behav Med. 2024 Mar 12;31(6):848–70. doi: 10.1007/s12529-024-10264-8 (PMC11588793; doi:10.1007/s12529-024-10264-8)
Supplement: Supplementary file 1 — Supplementary file1 (DOCX 568 KB) [file 12529_2024_10264_MOESM1_ESM.docx]

### Appendix A: Full Search Strategy by Database

| **PubMed** | **CINAHL Plus (via EBSCOhost)** | **Scopus** | **PsycInfo** | **Cochrane Library** |
| --- | --- | --- | --- | --- |
| (("Health Disparity, Minority and Vulnerable Populations"[Mesh] OR "Religion”[Mesh] OR “Persons”[Mesh] OR “Underserved community”[tiab] OR “Underprivileged community”[tiab] OR “Vulnerable Population”[tiab] OR “Underserved Population*”[tiab] OR “Underserved Patient”[tiab] OR “Underserved Patients”[tiab] OR “Disadvantaged Populations”[tiab] OR “Disadvantaged Population”[tiab] OR “Sensitive Populations”[tiab] OR “Sensitive Population Groups”[tiab] OR “Sensitive Population Group”[tiab] OR “patients”[tiab] OR “racial minor*”[tiab] OR “ethnic minor*”[tiab] OR “ethnoracial” OR “Hispanic” OR “Latin*” OR "Black" OR "Asian" OR "Native American" OR "Native Hawaiian" OR "Pacific Islander" OR "Alaska Native" OR "low socioeconomic status" OR "lower socioeconomic status" OR "low income" OR "lower income” OR "low SES" OR "lower SES" OR "rural" OR "non-metropolitan" OR "sexual minorit*" OR "gender minorit*" OR "refugees*" OR "immigrant*" OR "migrant*" OR "elderly" OR "limited English*" OR "foreign born" OR “foreign-born” OR "adolescent*” OR “young adult*” OR "incarcerated" OR "cultural group”) AND  (("Neoplasms"[Mesh] OR “Neoplasm*”[tiab] OR “Tumor*”[tiab] OR “Neoplasia*”[tiab] OR “Cancer*”[tiab] OR “Malignant Neoplasm*”[tiab] OR “Malignan*”[tiab] OR “Benign Neoplasm*”[tiab] OR "Hematologic*”[tiab]) AND ("Stage III" OR "Stage 3" OR "Stage IV" OR "Stage 4" OR "End stage" OR "Late stage" OR “Advanced” OR “Metastatic” OR “Incurable” OR “Terminal”))  AND  ("Psychosocial Intervention"[Mesh] OR "Psychosocial Support Systems"[Mesh] OR "Attitude to Death"[Mesh] OR "Psycho-Oncology"[Mesh] OR "Psychiatric Rehabilitation"[Mesh] OR "Cognitive Behavioral Therapy"[Mesh] OR  “Psychotherapy”[Mesh] OR "Adaptation, Psychological"[Mesh] OR "Mindfulness"[Mesh] OR "Music Therapy"[Mesh] OR "Social Support"[Mesh] OR "Adaptation, Psychological"[Mesh] OR "Mindfulness"[Mesh] OR "Music Therapy"[Mesh] OR "Social Support"[Mesh] OR “Terminal Care"[Mesh] OR "Hospice and Palliative Care Nursing"[Mesh] OR "Palliative Care"[Mesh] OR "Hospice Care"[Mesh] OR “Psychosocial Intervention*”[tiab] OR “Psychological Intervention*”[tiab] OR “Psychosocial Support System*”[tiab] OR “Social Support System*”[tiab] OR “Psychosocial Support*”[tiab]  “Psychological Support System*”[tiab] OR “Attitudes to Death”[tiab] OR “Psychosocial Oncology”[tiab] OR “Psychiatric Rehabilitation”[tiab] OR “Mental Health Rehabilitation”[tiab] OR “Psychosocial Rehabilitation”[tiab] OR “Psychosocial Care*”[tiab] OR "Mental Health”[tiab] OR "Counseling"[tiab] OR "Rehabilitation"[tiab] OR "Social Support"[tiab] OR “cultural adaptation”[tiab] OR “Cultural adaptation” OR “Culturally adapted” OR “Cognitive behavior* Therap*”[tiab] OR “Cognitive Therapy”[tiab] OR “Cognitive Behavior Therapies”[tiab] OR “Cognitive Psychotherap*”[tiab] OR “Cognitive Therapies”[tiab] OR “Cognition Therap*”[tiab] OR “Psychological Adaptation”[tiab] OR “Adjustment”[tiab] OR “Coping Behavior*”[tiab] OR “Coping Skill*”[tiab] OR “Coping Strateg*”[tiab] OR “Adaptive Behavior*”[tiab] OR “music therapy”[tiab] OR “complementary alternative medicine” OR “complementary therapies” OR “stress management” OR “psychotherapy” OR “patient education” OR “behavioral intervention” OR “meaning centered” OR “legacy” OR “dignity” OR “non-pharmacological” OR “alternative therapy” OR “supportive” OR “expressive therapy” OR “creative arts” OR “art therapy” OR “expressive writing” OR “acceptance-based” OR “acceptance and commitment therapy” OR “relaxation” OR “guided imagery” OR “hypnosis” OR “physical activity” OR “self-management” OR “storytelling” OR “narrative” OR “nutritional” OR “spiritual” OR “religion” OR “communication” OR “Palliative Care”[tiab] OR “Palliative Treatment*”[tiab] OR “Palliative Therapy”[tiab] OR “Palliative Supportive Care”[tiab] OR “Palliative Surgery”[tiab] OR “Palliative Nursing”[tiab] OR “Palliative Care Nursing”[tiab] OR “Hospice Nursing”[tiab] OR “Hospice Care”[tiab] OR “Hospice Programs”[tiab] OR “Hospice Program”[tiab])  NOT ("Comment”[Publication Type] OR “Practice Guideline”[Publication Type] OR “Guideline”[Publication Type] OR "Case Reports”[Publication Type] OR "Drug therapy”[tiab] OR "Radiotherapy”[tiab] OR "Imaging”[tiab] OR "Pathology”[tiab] OR “Surgical Procedures, Operative”[Mesh] OR "Cells”[Mesh] OR "Retrospective Studies”[Mesh] OR "Synthetic Biology"[Mesh] OR "Immunohistochemistry"[Mesh] OR "Radiography"[Mesh] OR "Bibliometrics"[Mesh] OR "Biocompatible Materials"[Mesh] OR "Tissue Engineering"[Mesh] OR "Drug therapy"[Subheading] OR "Radiotherapy"[Subheading] OR "Diagnostic Imaging"[Subheading] OR "Pathology"[Subheading] OR "Genetics"[Subheading] OR "Pharmacology"[Subheading] OR "Surgery"[Subheading] OR "Diagnosis"[Subheading] OR "Enzymology"[Subheading] OR "Biosynthesis"[Subheading] OR "Etiology"[Subheading] OR "Physiopathology"[Subheading] OR "Chemistry"[Subheading] OR "Metabolism"[Subheading] OR “Instrumentation”[Subheading] OR “Standards”[Subheading])) | (((MH "Health Disparity, Minority and Vulnerable Populations+") OR (MH Religion+) OR (MH Persons+) OR (TI "Underserved community" OR AB "Underserved community") OR (TI "Underprivileged community" OR AB "Underprivileged community") OR (TI "Vulnerable Population" OR AB "Vulnerable Population") OR (TI "Underserved Population*" OR AB "Underserved Population*") OR (TI "Underserved Patient" OR AB "Underserved Patient") OR (TI "Underserved Patients" OR AB "Underserved Patients") OR (TI "Disadvantaged Populations" OR AB "Disadvantaged Populations") OR (TI "Disadvantaged Population" OR AB "Disadvantaged Population") OR (TI "Sensitive Populations" OR AB "Sensitive Populations") OR (TI "Sensitive Population Groups" OR AB "Sensitive Population Groups") OR (TI "Sensitive Population Group" OR AB "Sensitive Population Group") OR (TI patients OR AB patients) OR (TI "racial minor*" OR AB "racial minor*") OR (TI "ethnic minor*" OR AB "ethnic minor*") OR ethnoracial OR Hispanic OR Latin* OR Black OR Asian OR "Native American" OR "Native Hawaiian" OR "Pacific Islander" OR "Alaska Native" OR "low socioeconomic status" OR "lower socioeconomic status" OR "low income" OR "lower income" OR "low SES" OR "lower SES" OR rural OR non-metropolitan OR "sexual minorit*" OR "gender minorit*" OR refugees* OR immigrant* OR migrant* OR elderly OR "limited English*" OR "foreign born" OR foreign-born OR adolescent* OR "young adult*" OR incarcerated OR "cultural group" )  AND  (((MH Neoplasms+) OR (TI Neoplasm* OR AB Neoplasm*) OR (TI Tumor* OR AB Tumor*) OR (TI Neoplasia* OR AB Neoplasia*) OR (TI Cancer* OR AB Cancer*) OR (TI "Malignant Neoplasm*" OR AB "Malignant Neoplasm*") OR (TI Malignan* OR AB Malignan*) OR (TI "Benign Neoplasm*" OR AB "Benign Neoplasm*") OR (TI Hematologic* OR AB Hematologic*)) AND ("Stage III" OR "Stage 3" OR "Stage IV" OR "Stage 4" OR "End stage" OR "Late stage" OR Advanced OR Metastatic OR Incurable OR Terminal ))  AND  ((MH "Psychosocial Intervention+") OR (MH "Psychosocial Support Systems+") OR (MH "Attitude to Death+") OR (MH Psycho-Oncology+) OR (MH "Psychiatric Rehabilitation+") OR (MH "Cognitive Behavioral Therapy+") OR (MH Psychotherapy+) OR (MH "Adaptation, Psychological+") OR (MH Mindfulness+) OR (MH "Music Therapy+") OR (MH "Social Support+") OR (MH "Adaptation, Psychological+") OR (MH Mindfulness+) OR (MH "Music Therapy+") OR (MH "Social Support+") OR (MH "Terminal Care+") OR (MH "Hospice and Palliative Care Nursing+") OR (MH "Palliative Care+") OR (MH "Hospice Care+") OR (TI "Psychosocial Intervention*" OR AB "Psychosocial Intervention*") OR (TI "Psychological Intervention*" OR AB "Psychological Intervention*") OR (TI "Psychosocial Support System*" OR AB "Psychosocial Support System*") OR (TI "Social Support System*" OR AB "Social Support System*") OR (TI "Psychosocial Support*" OR AB "Psychosocial Support*") OR (TI "Psychological Support System*" OR AB "Psychological Support System*") OR (TI "Attitudes to Death" OR AB "Attitudes to Death") OR (TI "Psychosocial Oncology" OR AB "Psychosocial Oncology") OR (TI "Psychiatric Rehabilitation" OR AB "Psychiatric Rehabilitation") OR (TI "Mental Health Rehabilitation" OR AB "Mental Health Rehabilitation") OR (TI "Psychosocial Rehabilitation" OR AB "Psychosocial Rehabilitation") OR (TI "Psychosocial Care*" OR AB "Psychosocial Care*") OR (TI "Mental Health" OR AB "Mental Health") OR (TI Counseling OR AB Counseling) OR (TI Rehabilitation OR AB Rehabilitation) OR (TI "Social Support" OR AB "Social Support") OR (TI "cultural adaptation" OR AB "cultural adaptation") OR "Cultural adaptation" OR "Culturally adapted" OR (TI "Cognitive behavior* Therap*" OR AB "Cognitive behavior* Therap*") OR (TI "Cognitive Therapy" OR AB "Cognitive Therapy") OR (TI "Cognitive Behavior Therapies" OR AB "Cognitive Behavior Therapies") OR (TI "Cognitive Psychotherap*" OR AB "Cognitive Psychotherap*") OR (TI "Cognitive Therapies" OR AB "Cognitive Therapies") OR (TI "Cognition Therap*" OR AB "Cognition Therap*") OR (TI "Psychological Adaptation" OR AB "Psychological Adaptation") OR (TI Adjustment OR AB Adjustment) OR (TI "Coping Behavior*" OR AB "Coping Behavior*") OR (TI "Coping Skill*" OR AB "Coping Skill*") OR (TI "Coping Strateg*" OR AB "Coping Strateg*") OR (TI "Adaptive Behavior*" OR AB "Adaptive Behavior*") OR (TI "music therapy" OR AB "music therapy") OR "complementary alternative medicine" OR "complementary therapies" OR "stress management" OR psychotherapy OR "patient education" OR "behavioral intervention" OR "meaning centered" OR legacy OR dignity OR non-pharmacological OR "alternative therapy" OR supportive OR "expressive therapy" OR "creative arts" OR "art therapy" OR "expressive writing" OR acceptance-based OR "acceptance and commitment therapy" OR relaxation OR "guided imagery" OR hypnosis OR "physical activity" OR self-management OR storytelling OR narrative OR nutritional OR spiritual OR religion OR communication OR (TI "Palliative Care" OR AB "Palliative Care") OR (TI "Palliative Treatment*" OR AB "Palliative Treatment*") OR (TI "Palliative Therapy" OR AB "Palliative Therapy") OR (TI "Palliative Supportive Care" OR AB "Palliative Supportive Care") OR (TI "Palliative Surgery" OR AB "Palliative Surgery") OR (TI "Palliative Nursing" OR AB "Palliative Nursing") OR (TI "Palliative Care Nursing" OR AB "Palliative Care Nursing") OR (TI "Hospice Nursing" OR AB "Hospice Nursing") OR (TI "Hospice Care" OR AB "Hospice Care") OR (TI "Hospice Programs" OR AB "Hospice Programs") OR (TI "Hospice Program" OR AB "Hospice Program"))  NOT  ((PT Comment) OR (PT "Practice Guideline") OR (PT Guideline) OR (PT "Case Reports") OR (TI "Drug therapy" OR AB "Drug therapy") OR (TI Radiotherapy OR AB Radiotherapy) OR (TI Imaging OR AB Imaging) OR (TI Pathology OR AB Pathology) OR (MH "Surgical Procedures, Operative+") OR (MH Cells+) OR (MH "Retrospective Studies+") OR (MH "Synthetic Biology+") OR (MH Immunohistochemistry+) OR (MH Radiography+) OR (MH Bibliometrics+) OR (MH "Biocompatible Materials+") OR (MH "Tissue Engineering+") OR "Drug Therapy" OR "Radiotherapy" OR "Diagnostic Imaging" OR "Pathology" OR "Genetics" OR "Pharmacology" OR "Surgery" OR "Diagnosis" OR "Enzymology" OR "Biosynthesis" OR "Etiology" OR "Physiopathology" OR "Chemistry" OR "Metabolism" OR Instrumentation[Subheading] OR Standards[Subheading] )) | ((INDEXTERMS("Health Disparity, Minority and Vulnerable populations") OR INDEXTERMS(religion) OR INDEXTERMS(persons) OR TITLE-ABS("Underserved populations") OR TITLE-ABS("Underserved community") OR TITLE-ABS("Underprivileged community") OR TITLE-ABS("Vulnerable Population") OR TITLE-ABS("Underserved Population") OR TITLE-ABS("Underserved Patient") OR TITLE-ABS("Underserved Patients") OR TITLE-ABS("Disadvantaged Populations") OR TITLE-ABS("Disadvantaged Population") OR TITLE-ABS("Sensitive Populations") OR TITLE-ABS("Sensitive Population Groups") OR TITLE-ABS("Sensitive Population Group") OR TITLE-ABS(patients) OR TITLE-ABS("racial minor*") OR TITLE-ABS("ethnic minor*") OR ethnoracial OR Hispanic OR Latin* OR Black OR Asian OR {Native American} OR {Native Hawaiian} OR {Pacific Islander} OR {Alaska Native} OR {low socioeconomic status} OR {lower socioeconomic status} OR {low income} OR {lower income} OR {low SES} OR {lower SES} OR rural OR non-metropolitan OR "sexual minorit*" OR "gender minorit*" OR refugees* OR immigrant* OR migrant* OR elderly OR "limited English*" OR {foreign born} OR foreign-born OR adolescent* OR "young adult*" OR incarcerated OR {cultural group})  AND  (INDEXTERMS(Neoplasms) OR TITLE-ABS(Neoplasm*) OR TITLE-ABS(Tumor*) OR TITLE-ABS(Neoplasia*) OR TITLE-ABS(Cancer*) OR TITLE-ABS("Malignant Neoplasm*") OR TITLE-ABS(Malignan*) OR TITLE-ABS("Benign Neoplasm*") OR TITLE-ABS(Hematologic*)) AND ({Stage III} OR {Stage 3} OR {Stage IV} OR {Stage 4} OR {End stage} OR {Late stage} OR Advanced OR Metastatic OR Incurable OR Terminal)  AND  (INDEXTERMS("Psychosocial Intervention") OR INDEXTERMS("Psychosocial Support Systems") OR INDEXTERMS("Attitude to Death") OR INDEXTERMS(Psycho-Oncology) OR INDEXTERMS("Psychiatric Rehabilitation") OR INDEXTERMS("Cognitive Behavioral Therapy") OR INDEXTERMS(Psychotherapy) OR INDEXTERMS("Adaptation, Psychological") OR INDEXTERMS(Mindfulness) OR INDEXTERMS("Music Therapy") OR INDEXTERMS("Social Support") OR INDEXTERMS("Adaptation, Psychological") OR INDEXTERMS(Mindfulness) OR INDEXTERMS("Music Therapy") OR INDEXTERMS("Social Support") OR INDEXTERMS("Terminal Care") OR INDEXTERMS("Hospice and Palliative Care Nursing") OR INDEXTERMS("Palliative Care") OR INDEXTERMS("Hospice Care") OR TITLE-ABS("Psychosocial Intervention*") OR TITLE-ABS("Psychological Intervention*") OR TITLE-ABS("Psychosocial Support System*") OR TITLE-ABS("Social Support System*") OR TITLE-ABS("Psychosocial Support*") OR TITLE-ABS("Psychological Support System*") OR TITLE-ABS("Attitudes to Death") OR TITLE-ABS("Psychosocial Oncology") OR TITLE-ABS("Psychiatric Rehabilitation") OR TITLE-ABS("Mental Health Rehabilitation") OR TITLE-ABS("Psychosocial Rehabilitation") OR TITLE-ABS("Psychosocial Care*") OR TITLE-ABS("Mental Health") OR TITLE-ABS(Counseling) OR TITLE-ABS(Rehabilitation) OR TITLE-ABS("Social Support") OR TITLE-ABS("cultural adaptation") OR "Cultural adaptation" OR "Culturally adapted" OR TITLE-ABS("Cognitive behavior* Therap*") OR TITLE-ABS("Cognitive Therapy") OR TITLE-ABS("Cognitive Behavior Therapies") OR TITLE-ABS("Cognitive Psychotherap*") OR TITLE-ABS("Cognitive Therapies") OR TITLE-ABS("Cognition Therap*") OR TITLE-ABS("Psychological Adaptation") OR TITLE-ABS(Adjustment) OR TITLE-ABS("Coping Behavior*") OR TITLE-ABS("Coping Skill*") OR TITLE-ABS("Coping Strateg*") OR TITLE-ABS("Adaptive Behavior*") OR TITLE-ABS("music therapy") OR {complementary alternative medicine} OR  {complementary therapies} OR {stress management} OR psychotherapy OR {patient education} OR {behavioral intervention} OR {meaning centered} OR legacy OR dignity OR non-pharmacological OR {alternative therapy} OR supportive OR {expressive therapy} OR {creative arts} OR {art therapy} OR {expressive writing} OR acceptance-based OR {acceptance and commitment therapy} OR relaxation OR {guided imagery} OR hypnosis  OR {physical activity} OR self-management OR storytelling OR narrative OR nutritional OR spiritual OR religion OR communication OR TITLE-ABS("Palliative Care") OR TITLE-ABS("Palliative Treatment*") OR TITLE-ABS("Palliative Therapy") OR TITLE-ABS("Palliative Supportive Care") OR TITLE-ABS("Palliative Surgery") OR TITLE-ABS("Palliative Nursing") OR TITLE-ABS("Palliative Care Nursing") OR TITLE-ABS("Hospice Nursing") OR TITLE-ABS("Hospice Care") OR TITLE-ABS("Hospice Programs") OR TITLE-ABS("Hospice Program"))  AND NOT  (DOCTYPE(Comment) OR DOCTYPE("Practice Guideline") OR DOCTYPE(Guideline) OR DOCTYPE("Case Reports") OR TITLE-ABS("Drug therapy") OR TITLE-ABS(Radiotherapy) OR TITLE-ABS(Imaging) OR TITLE-ABS(Pathology) OR INDEXTERMS("Surgical Procedures, Operative") OR INDEXTERMS(Cells) OR INDEXTERMS("Retrospective Studies") OR INDEXTERMS("Synthetic Biology") OR INDEXTERMS(Immunohistochemistry) OR INDEXTERMS(Radiography) OR INDEXTERMS(Bibliometrics) OR INDEXTERMS("Biocompatible Materials") OR INDEXTERMS("Tissue Engineering") OR {Drug Therapy} OR {Radiotherapy} OR {Diagnostic Imaging} OR {Pathology} OR {Genetics} OR {Pharmacology} OR {Surgery} OR {Diagnosis} OR {Enzymology} OR {Biosynthesis} OR {Etiology} OR {Physiopathology} OR {Chemistry} OR {Metabolism})  AND ( LIMIT-TO ( EXACTKEYWORD , "Human" ) OR LIMIT-TO ( EXACTKEYWORD , "Humans" ) ) AND ( LIMIT-TO ( LANGUAGE , "English" ) ) AND ( LIMIT-TO ( SRCTYPE , "j" ) ) AND ( LIMIT-TO ( DOCTYPE , "ar" ) OR LIMIT-TO ( DOCTYPE , "re" ) OR LIMIT-TO ( DOCTYPE , "cp" ) ) AND ( LIMIT-TO ( SUBJAREA , "MEDI" ) OR LIMIT-TO ( SUBJAREA , "NURS" ) OR LIMIT-TO ( SUBJAREA , "MULT" ) OR LIMIT-TO ( SUBJAREA , "NEUR" ) OR LIMIT-TO ( SUBJAREA , "PSYC" ) OR LIMIT-TO ( SUBJAREA , "HEAL" ) OR LIMIT-TO ( SUBJAREA , "SOCI" ) OR LIMIT-TO ( SUBJAREA , "ARTS" ) OR LIMIT-TO ( SUBJAREA , "DECI" ) OR LIMIT-TO ( SUBJAREA , "Undefined" )))  Filters:  Subject Areas:   - Medicine - Nursing - Multidisciplinary - Neuroscience - Psychology - Health professions - Social sciences - arts and humanities - decision sciences - undefined   Language:   - english   Document Type:   - Article - Review - Conference Paper   Keyword:   - limited to Human - Limited to Humans | (exp “Psychosocial Intervention”/ OR exp “Psychosocial Support Systems”/ OR exp “Attitude to Death”/ OR exp “Psycho-Oncology”/ OR exp “Psychiatric Rehabilitation”/ OR TIAB(“Psychosocial Intervention*”) OR TIAB(“Psychological Intervention*”) OR TIAB(“Psychosocial Support System*”) OR TIAB(“Social Support System*”) OR TIAB(“Social Support Systems”) OR TIAB(“Psychosocial Support*”) OR TIAB(“Psychological Support System*”) OR TIAB(“Attitudes to Death”) OR TIAB(“Psycho Oncology”) OR TIAB(“Psychosocial Oncology”) OR TIAB(“Oncology, Psychosocial”) OR TIAB(“Psychooncology”) OR TIAB(“Psychiatric Rehabilitation”) OR TIAB(“Mental Health Rehabilitation”) OR TIAB(“Rehabilitation, Mental Health”) OR TIAB(“Psychosocial Rehabilitation”) OR TIAB(“Rehabilitation, Psychosocial”) OR TIAB(“Psychosocial Care*”) OR TIAB(“Care, Psychosocial”) OR TIAB(“Mental Health”) OR TIAB(“Counseling”) OR TIAB(“Rehabilitation”) OR TIAB(“Social Support”) OR TIAB(“Cultural adaptation”) OR “cultural adaptation” OR “Culturally adapted” OR exp “Cognitive Behavioral Therapy”/ OR TIAB(“Cognitive behavior* therap*”) OR TIAB(“Cognitive behaviour* therap*”) OR TIAB(“Cognitive Therapy”) OR TIAB(“Cognitive Behavior Therapies”) OR TIAB(“Cognitive Psychotherap*”) OR TIAB(“Cognitive Therapies”) OR TIAB(“Cognition Therap*”) OR exp “Psychotherapy”/ OR exp “Adaptation, Psychological”/ OR “Psychologic Adaptation” OR TIAB(“Psychological Adaptation”) OR TIAB(“Adjustment”) OR TIAB(“Coping Behavior*”) OR TIAB(“Coping Skill*”) OR TIAB(“Coping Strateg*”) OR TIAB(“Adaptive Behavior*”) OR exp “Mindfulness”/ OR exp “Music Therapy”/ OR exp “Social Support”/ OR TIAB(“music therapy”) OR exp “Terminal Care”/ OR exp “Hospice and Palliative Care Nursing”/ OR exp “Palliative care”/ OR exp “Hospice care”/ OR “end-of-life care” OR TIAB(“End of Life Care”) OR TIAB(“Palliative Care”) OR TIAB(“Palliative Treatment*”) OR TIAB(“Palliative Therapy”) OR TIAB(“Palliative Supportive Care”) OR TIAB(“Palliative Surgery”) OR TIAB(“Palliative Nursing”) OR TIAB(“Palliative Care Nursing”) OR TIAB(“Hospice Nursing”) OR TIAB(“Hospice Care”) OR TIAB(“Hospice Programs”) OR TIAB(“Hospice Program”) OR “complementary alternative medicine” OR “complementary therapies” OR “stress management” OR “psychotherapy” OR “patient education” OR “behavioral intervention” OR “meaning centered” OR Legacy OR dignity OR non-pharmacological OR “alternative therapy” OR supportive OR “expressive therapy” OR “creative arts” OR “art therapy” OR “expressive writing” OR “acceptance-based” OR “acceptance and commitment therapy” OR relaxation OR “guided imagery” OR hypnosis OR “physical activity” OR self-management OR Storytelling OR narrative OR nutritional OR spiritual OR religion OR “communication”)  AND  (exp “Neoplasms”/ OR exp “Neoplasm*”/ OR TIAB(“Tumor*”) OR TIAB(“Neoplasm”) OR TIAB(“Tumors”) OR TIAB(“Neoplasia*”) OR TIAB(“Cancer*”) OR TIAB(“Cancers”) OR TIAB(“Malignant Neoplasm*”) OR TIAB(“Malignan*”) OR TIAB(“Benign Neoplasm*”) OR TIAB(“Hematologic*”))  AND  (“Stage III” OR “Stage 3” OR “Stage IV” OR “Stage 4” OR “End stage” OR “Late stage” OR Advanced OR Metastatic OR Incurable OR Terminal)  AND  (exp “Health Disparity, Minority and Vulnerable Populations”/ OR TIAB(“underserved community”) OR TIAB(“underprivileged community”) OR TIAB(“Population, Vulnerable”) OR TIAB(“Vulnerable Population”) OR TIAB(“Population, Underserved”) OR TIAB(“Populations, Underserved”) OR TIAB(“Underserved Population”) OR TIAB(“Underserved Populations”) OR TIAB(“Underserved Patient”) OR TIAB(“Underserved Patients”) OR TIAB(“Disadvantaged Population”) OR TIAB(“Disadvantaged Populations”) OR TIAB(“Population, Disadvantaged”) OR TIAB(“Sensitive Population”) OR TIAB(“Sensitive Populations”) OR TIAB(“Population, Sensitive”) OR TIAB(“Sensitive Population Groups”) OR (“Sensitive Population Group”) OR TIAB(“racial minor*”) OR TIAB(“ethnic minor*”) OR ethnoracial OR Hispanic OR Latin* OR Black OR Asian OR “Native American” OR “Native Hawaiian” OR “Pacific Islander” OR “Alaska Native” OR “low socioeconomic status” OR “lower socioeconomic status” OR “low income” OR “lower income” OR “low SES” OR “lower SES” OR rural OR non-metropolitan OR “sexual minorit*” OR “gender minorit*” OR refugees* OR immigrant* OR migrant* OR elderly OR “limited English*” OR “foreign born” OR “foreign-born” OR adolescent* OR “young adult*” OR incarcerated OR “cultural group” OR exp religion/ OR exp persons/ OR TIAB(patients))  NOT  (Comment.pt. OR "Practice Guideline".pt. OR Guideline.pt. OR "Case Reports".pt. OR "TIAB("Drug therapy" ) OR TIAB("Radiotherapy”) OR TIAB("Imaging”) OR TIAB("Pathology”) OR exp "“Surgical Procedures, Operative"/ OR exp "Cells”/ OR exp "Retrospective Studies”/ OR exp "Synthetic Biology"/ OR exp Immunohistochemistry/ OR exp Radiography/ OR exp Bibliometrics/ OR exp "Biocompatible Materials"/ OR exp "Tissue Engineering"/ OR "Drug Therapy" OR "Radiotherapy" OR "Diagnostic Imaging" OR "Pathology" OR "Genetics" OR "Pharmacology" OR "Surgery" OR "Diagnosis" OR "Enzymology" OR "Biosynthesis" OR "Etiology" OR "Physiopathology" OR "Chemistry" OR "Metabolism" OR Instrumentation[Subheading] OR Standards[Subheading])  Filters: English Language, Humans, Scholarly Journals | (("Psychosocial" NEXT Intervention*) OR ("Psychological" NEXT Intervention*) OR ("Psychosocial Support" NEXT System*) OR ("Social Support" NEXT System*) OR ("Psychosocial" NEXT Support*) OR ("Psychological Support" NEXT System*) OR "Attitudes to Death" OR "Psychosocial Oncology" OR "Psychiatric Rehabilitation" OR "Mental Health Rehabilitation" OR "Psychosocial Rehabilitation" OR ("Psychosocial" NEXT Care*) OR "Mental Health" OR Counseling OR Rehabilitation OR "Social Support" OR "cultural adaptation" OR "Cultural adaptation" OR "Culturally adapted" OR ("Cognitive" NEXT behavior* NEXT Therapy) OR ("Cognitive" NEXT behavior* NEXT Therapies) OR "Cognitive Therapy" OR "Cognitive Behavior therapies" OR "Cognitive Psychotherapy” OR "Cognitive Psychotherapies” OR "Cognitive Therapies" OR "Cognition Therapy" OR “Cognition Therapies” OR "Psychological Adaptation" OR Adjustment OR ("Coping" NEXT Behavior*) OR ("Coping" NEXT Skill*) OR "Coping Strategy” OR "Coping Strategies” OR ("Adaptive" NEXT Behavior*) OR "music therapy" OR "complementary alternative medicine" OR "complementary therapies" OR "stress management" OR psychotherapy OR "patient education" OR "behavioral intervention" OR "meaning centered" OR legacy OR dignity OR non-pharmacological OR "alternative therapy" OR supportive OR "expressive therapy" OR "creative arts" OR "art therapy" OR "expressive writing" OR acceptance-based OR "acceptance and commitment therapy" OR relaxation OR "guided imagery" OR hypnosis OR "physical activity" OR self-management OR storytelling OR narrative OR nutritional OR spiritual OR religion OR Communication OR "Palliative Care" OR ("Palliative" NEXT Treatment*) OR "Palliative Therapy" OR "Palliative Supportive Care" OR "Palliative Surgery" OR "Palliative Nursing" OR "Palliative Care Nursing" OR "Hospice Nursing" OR "Hospice Care" OR "Hospice Programs" OR "Hospice Program")  AND  ([mh "Health Disparity, Minority and Vulnerable Populations"] OR [mh religion] OR [mh persons] OR "Underserved populations":ti,ab OR "Underserved community":ti,ab OR "Underprivileged community":ti,ab OR "Vulnerable Population":ti,ab OR "Underserved Population":ti,ab OR "Underserved Patient":ti,ab OR "Underserved Patients":ti,ab OR "Disadvantaged Populations":ti,ab OR "Disadvantaged Population":ti,ab OR "Sensitive Populations":ti,ab OR "Sensitive Population Groups":ti,ab OR "Sensitive Population Group":ti,ab OR patients:ti,ab OR ("racial" NEXT minor*):ti,ab OR ("ethnic" NEXT minor*):ti,ab OR  Ethnoracial OR Hispanic OR Latin* OR Black OR Asian OR "Native American" OR "Native Hawaiian" OR "Pacific Islander" OR "Alaska Native" OR "low socioeconomic status" OR "lower socioeconomic status" OR "low income" OR "lower income" OR "low SES" OR "lower SES" OR rural OR non-metropolitan OR ("sexual" NEXT minorit*) OR ("gender" NEXT minorit*) OR refugees* OR immigrant* OR migrant* OR elderly OR ("limited" NEXT English*) OR "foreign born" OR foreign-born OR adolescent* OR ("young" NEXT adult*) OR incarcerated OR "cultural group")  AND  ([mh Neoplasms] OR Neoplasm*:ti,ab OR Tumor*:ti,ab OR Neoplasia*:ti,ab OR Cancer*:ti,ab OR (Malignant NEXT Neoplasm*):ti,ab OR Malignan*:ti,ab OR (Benign NEXT Neoplasm*):ti,ab OR Hematologic*:ti,ab)  AND  ("Stage III" OR "Stage 3" OR "Stage IV" OR "Stage 4" OR "End stage" OR "Late stage" OR Advanced OR Metastatic OR Incurable OR Terminal) |

### Appendix B: Quality/Bias Assessment of Studies Included in Qualitative Synthesis & Quantitative Meta-Analysis

| **Criteria** | **Q1** | **Q2** | **Q3** | **Q4** | **Total^a^ (/4)** |  |  |  |  |  |  |  |  |  |
| --- | --- | --- | --- | --- | --- | --- | --- | --- | --- | --- | --- | --- | --- | --- |
| **Qualitative & Feasibility/Acceptability Studies (k = 23)** | | | | | | | | | | | | | | |
| [Bekelman 2020](https://onlinelibrary.wiley.com/doi/10.1002/pon.5313) | 1 | 0 | 1 | 0 | 2/4 |  |  |  |  |  |  |  |  |  |
| [Chen 2022](https://journals.sagepub.com/doi/full/10.1177/02692163211066736) | 1 | 0 | 1 | 1 | 3/4 |  |  |  |  |  |  |  |  |  |
| [Fink 2020](https://regroup-production.s3.amazonaws.com/documents/ReviewReference/606470695/Fink%20et%20al.%20-%202020%20-%20A%20Qualitative%20Analysis%20of%20a%20Palliative%20Care-Focuse.pdf?response-content-type=application%2Fpdf&X-Amz-Algorithm=AWS4-HMAC-SHA256&X-Amz-Credential=AKIAYSFKCAWYQ4D5IUHG%2F20230406%2Fus-east-1%2Fs3%2Faws4_request&X-Amz-Date=20230406T192925Z&X-Amz-Expires=604800&X-Amz-SignedHeaders=host&X-Amz-Signature=652cc7a52611eae8fbc33cb94b05b16445926244449a667cfa31ee4871597513) | 1 | 0 | 1 | 0 | 2/4 |  |  |  |  |  |  |  |  |  |
| [Houmann 2010](https://bmcpalliatcare.biomedcentral.com/articles/10.1186/1472-684X-9-21) | 1 | 0 | 1 | 1 | 3/4 |  |  |  |  |  |  |  |  |  |
| [Kwan 2019](https://www.sciencedirect.com/science/article/pii/S0020748918302645?via%3Dihub) | 1 | 0 | 0 | 0 | 1/4 |  |  |  |  |  |  |  |  |  |
| [Leng 2018](https://link.springer.com/article/10.1007/s10903-017-0591-7) | 1 | 0 | 1 | 1 | 3/4 |  |  |  |  |  |  |  |  |  |
| [Leng 2019](https://link.springer.com/article/10.1007/s00520-019-4638-2) | 1 | 1 | 1 | 1 | 4/4 |  |  |  |  |  |  |  |  |  |
| [Li 2014](https://onlinelibrary.wiley.com/doi/10.1111/jan.12455) | 1 | 0 | 1 | 1 | 3/4 |  |  |  |  |  |  |  |  |  |
| [Lin 2020](https://journals.sagepub.com/doi/full/10.1177/0269216320902666?rfr_dat=cr_pub++0pubmed&url_ver=Z39.88-2003&rfr_id=ori%3Arid%3Acrossref.org) | 1 | 0 | 1 | 1 | 3/4 |  |  |  |  |  |  |  |  |  |
| [Lin 2022](https://onlinelibrary.wiley.com/doi/10.1111/ecc.13670) | 1 | 1 | 1 | 1 | 4/4 |  |  |  |  |  |  |  |  |  |
| [Liossi 2001](https://onlinelibrary.wiley.com/doi/10.1002/ch.228) | 1 | 0 | 1 | 0 | 2/4 |  |  |  |  |  |  |  |  |  |
| [Nunziante 2021](https://bmcpalliatcare.biomedcentral.com/articles/10.1186/s12904-021-00821-3) | 1 | 0 | 1 | 1 | 3/4 |  |  |  |  |  |  |  |  |  |
| [Ólafsdóttir 2018](https://regroup-production.s3.amazonaws.com/documents/ReviewReference/603442908/%C3%93lafsd%C3%B3ttir%20KL%20et%20al.%20-%202018%20-%20Integrating%20nurse-facilitated%20advance%20care%20plannin.pdf?response-content-type=application%2Fpdf&X-Amz-Algorithm=AWS4-HMAC-SHA256&X-Amz-Credential=AKIAYSFKCAWYQ4D5IUHG%2F20230405%2Fus-east-1%2Fs3%2Faws4_request&X-Amz-Date=20230405T192321Z&X-Amz-Expires=604800&X-Amz-SignedHeaders=host&X-Amz-Signature=0d3b32e8fa1ca0dc2a89900d73d1ec3bf1c2c1668eb823747d261e9f6733e3fc) | 1 | 1 | 1 | 1 | 4/4 |  |  |  |  |  |  |  |  |  |
| [Patel 2019](https://www.ncbi.nlm.nih.gov/pmc/articles/PMC7046315/) | 1 | 0 | 1 | 1 | 3/4 |  |  |  |  |  |  |  |  |  |
| [Pon 2010](https://journals.sagepub.com/doi/epdf/10.2190/IL.18.2.e) | 0 | 0 | 0 | 0 | 0/4 |  |  |  |  |  |  |  |  |  |
| [Sakaguchi 2015](https://www.cambridge.org/core/journals/palliative-and-supportive-care/article/effectiveness-of-collage-activity-based-on-a-life-review-in-elderly-cancer-patients-a-preliminary-study/8EE31A5312D12E753B2083AC5C1E303C) | 0 | 0 | 1 | 0 | 1/4 |  |  |  |  |  |  |  |  |  |
| [Schulman-Green 2022](https://www.magonlinelibrary.com/doi/abs/10.12968/ijpn.2022.28.8.378?rfr_dat=cr_pub++0pubmed&url_ver=Z39.88-2003&rfr_id=ori%3Arid%3Acrossref.org) | 1 | 0 | 0 | 1 | 2/4 |  |  |  |  |  |  |  |  |  |
| [Takenouchi 2022](https://regroup-production.s3.amazonaws.com/documents/ReviewReference/603444002/Takenouchi%20S%20et%20al.%20-%202022%20-%20Strategies%20to%20Understand%20What%20Matters%20to%20Advanced%20.pdf?response-content-type=application%2Fpdf&X-Amz-Algorithm=AWS4-HMAC-SHA256&X-Amz-Credential=AKIAYSFKCAWYQ4D5IUHG%2F20230406%2Fus-east-1%2Fs3%2Faws4_request&X-Amz-Date=20230406T194221Z&X-Amz-Expires=604800&X-Amz-SignedHeaders=host&X-Amz-Signature=3770e559feaf4c41063ca80e6174c8569cd388cde5a5a488e0c0008b1cc7c22c) | 1 | 1 | 1 | 1 | 4/4 |  |  |  |  |  |  |  |  |  |
| [Torres-Blanco 2022](https://www.mdpi.com/2227-9032/10/7/1243ral%20Adaptation%20for%20Latinx%20Patients%20and%20Caregivers%20Coping%20with%20Advanced%20Cancer%20(mdpi.com)) | 1 | 0 | 1 | 1 | 3/4 |  |  |  |  |  |  |  |  |  |
| [vandenHurk 2015](https://journals.sagepub.com/doi/full/10.1177/0269216315572720?rfr_dat=cr_pub++0pubmed&url_ver=Z39.88-2003&rfr_id=ori%3Arid%3Acrossref.org) | 1 | 0 | 1 | 1 | 3/4 |  |  |  |  |  |  |  |  |  |
| [Wang 2019](https://regroup-production.s3.amazonaws.com/documents/ReviewReference/608337486/Wang%20et%20al.%20-%202020%20-%20The%20development%20of%20a%20family%20participatory%20dignity%20.pdf?response-content-type=application%2Fpdf&X-Amz-Algorithm=AWS4-HMAC-SHA256&X-Amz-Credential=AKIAYSFKCAWYQ4D5IUHG%2F20230417%2Fus-east-1%2Fs3%2Faws4_request&X-Amz-Date=20230417T201203Z&X-Amz-Expires=604800&X-Amz-SignedHeaders=host&X-Amz-Signature=35ebf95437425fca80d51c5d6490720de64d3a6c9c100f51dc3005f1719e2faf) | 1 | 0 | 1 | 0 | 2/4 |  |  |  |  |  |  |  |  |  |
| [Xiao 2012](https://onlinelibrary.wiley.com/doi/10.1111/j.1365-2702.2011.03842.x) | 1 | 0 | 1 | 1 | 3/4 |  |  |  |  |  |  |  |  |  |
| [Yang 2021](https://bmcpalliatcare.biomedcentral.com/articles/10.1186/s12904-021-00799-y) | 1 | 1 | 1 | 1 | 4/4 |  |  |  |  |  |  |  |  |  |
| **^a^** Each domain was scored a 1 if it was rated Yes or a 0 if it was rated Unclear or No. A total score was derived by summing up the scores for all domains. <2 poorer quality studies [[1]](https://paperpile.com/c/heY29X/87bE4).  Q1) The choice of study design was given and explained.  Q2) The selection of participants is described explicitly as, e.g., purposive, convenience, theoretical, and so forth.  Q3) The details of the data collection method are given, e.g., piloting, topic guides for interviews, number of items in a survey, use of open or closed items, validation, and so forth.  Q4) The details of analysis method are given, e.g., transcription and form of analysis (with reference to or full description of method), validation tests, and so forth. | | | | | | | | | | | | | | |
|  |  |  |  |  |  |  |  |  |  |  |  |  |  |  |
| **Criteria**  **Single-arm studies (k=10)** | **Q1** | **Q2** | **Q3** | **Q4** | **Q5** | **Q6** | **Q7** | **Q8** | **Total^b^ (/16)** |  |  |  |  |  |
| [Ando 2007](https://onlinelibrary.wiley.com/doi/epdf/10.1002/pon.1299) | 2 | 2 | 2 | 2 | 0 | 0 | 1 | 0 | 9/16 |  |  |  |  |  |
| [Ando 2016](https://www.tandfonline.com/doi/full/10.1080/07421656.2016.1128764?scroll=top&needAccess=true&role=tab&aria-labelledby=full-article) | 2 | 2 | 2 | 2 | 0 | 2 | 2 | 0 | 12/16 |  |  |  |  |  |
| [Bekelman 2019](https://onlinelibrary.wiley.com/doi/10.1002/pon.5313) | 2 | 2 | 2 | 2 | 2 | 2 | 1 | 1 | 14/16 |  |  |  |  |  |
| [Delrieu 2020](https://www.ncbi.nlm.nih.gov/pmc/articles/PMC7013652/) | 2 | 2 | 2 | 2 | 1 | 2 | 1 | 0 | 12/16 |  |  |  |  |  |
| [Kang 2015](https://journals.lww.com/jhpn/Abstract/2015/06000/Development_and_Preliminary_Testing_of_a.10.aspx) | 2 | 2 | 2 | 2 | 2 | 2 | 1 | 0 | 13/16 |  |  |  |  |  |
| [Li 2015](https://onlinelibrary.wiley.com/doi/10.1002/pon.3809) | 2 | 2 | 2 | 2 | 2 | 2 | 1 | 2 | 15/16 |  |  |  |  |  |
| [Niki 2019](https://www.liebertpub.com/doi/10.1089/jpm.2018.0527?url_ver=Z39.88-2003&rfr_id=ori:rid:crossref.org&rfr_dat=cr_pub%20%200pubmed) | 2 | 2 | 2 | 2 | 1 | 2 | 2 | 2 | 15/16 |  |  |  |  |  |
| [van den Hurk 2015](https://www.ncbi.nlm.nih.gov/pmc/articles/PMC4457793/) | 2 | 2 | 2 | 2 | 2 | 2 | 1 | 1 | 14/16 |  |  |  |  |  |
| [Wang 2020](https://onlinelibrary.wiley.com/doi/10.1111/ecc.13204) | 1 | 2 | 2 | 2 | 2 | 2 | 1 | 0 | 12/16 |  |  |  |  |  |
| [Warth 2018](https://spcare.bmj.com/content/8/2/167) | 2 | 2 | 2 | 2 | 1 | 2 | 1 | 0 | 12/16 |  |  |  |  |  |
| **^b^**Each domain was scored a 2 when reported, 1 when reported but inadequate, and 0 when not reported. A total score was derived by summing up the scores for all domains. <7 high risk of bias, 7–11 medium risk of bias, >11 low risk of bias [35, 62].  Q1) A clearly stated aim  Q2) Inclusion of consecutive patients  Q3) Prospective collection of data  Q4) Endpoints appropriate to the aim of the study  Q5) Unbiased assessment of the study endpoint  Q6) Follow-up period appropriate to the aim of the study  Q7) Loss to follow up less than 5%  Q8) Prospective calculation of the study size | | | | | | | | | | | | | | |
| **Criteria** | **Q1** | **Q2** | **Q3** | **Q4** | **Q5** | **Q6** | **Q7** | **Q8** | **Q9** | **Q10** | **Q11** | **Q12** | **Total^c^ (/24)** |  |
| **Quasi-Experimental Studies (k = 4)** | | | | | | | | | | | | | | |
| [Chimluang 2017](https://pubmed.ncbi.nlm.nih.gov/29173826/#:~:text=Conclusions%3A%20An%20intervention%20based%20on,of%20patients%20with%20terminal%20cancer.) | 2 | 2 | 2 | 2 | 1 | 2 | 2 | 2 | 2 | 2 | 2 | 2 | 23/24 |  |
| [Ichihara 2019](https://www.cambridge.org/core/journals/palliative-and-supportive-care/article/abs/effectiveness-of-spiritual-care-using-spiritual-pain-assessment-sheet-for-advanced-cancer-patients-a-pilot-nonrandomized-controlled-trial/75875AEEB6D30B38C5E3DB29A683492D) | 2 | 2 | 2 | 2 | 0 | 2 | 1 | 0 | 2 | 2 | 2 | 2 | 19/24 |  |
| [Li 2020](https://www.asian-nursingresearch.com/article/S1976-1317(20)30027-X/fulltext) | 2 | 2 | 2 | 2 | 2 | 2 | 1 | 2 | 2 | 2 | 2 | 2 | 23/24 |  |
| [Zhang 2019](https://onlinelibrary.wiley.com/doi/10.1111/jan.14018) | 2 | 2 | 2 | 2 | 2 | 2 | 1 | 2 | 2 | 2 | 2 | 2 | 23/24 |  |
| **^c^**Each domain was scored a 2 when reported, 1 when reported but inadequate, and 0 when not reported. A total score was derived by summing up the scores for all domains. <12 high risk of bias, 12–16 medium risk of bias, >16 low risk of bias [35, 62].  Q1-Q8) same criteria as for Single-arm studies  Q9) An adequate control group  Q10) Contemporary groups  Q11) Baseline equivalence of groups  Q12) Adequate statistical analysis | | | | | | | | | | | | | | |
| **Criteria**  **RCTs (k = 25)** | **Q1** | **Q2** | **Q3** | **Q4** | **Q5** | **Q6** | **Q7** | **Q8** | **Q9** | **Q10** | **Q11** | **Q12** | **Q13** | **Total^d^ (/26)** |
| [Anderson 2004](https://ascopubs.org/doi/10.1200/JCO.2004.06.115?url_ver=Z39.88-2003&rfr_id=ori:rid:crossref.org&rfr_dat=cr_pub%20%200pubmed) | 1 | 0 | 2 | 0 | 0 | 2 | 2 | 2 | 0 | 2 | 2 | 2 | 2 | 17/26 |
| [Ando 2010](https://www.jpsmjournal.com/article/S0885-3924(10)00248-4/fulltext) | 2 | 2 | 2 | 0 | 0 | 2 | 0 | 2 | 1 | 2 | 2 | 2 | 2 | 19/26 |
| [Catania 2021](https://www.ejoncologynursing.com/article/S1462-3889(21)00067-3/fulltext) | 1 | 0 | 2 | 0 | 0 | 2 | 2 | 2 | 0 | 2 | 2 | 2 | 2 | 17/26 |
| [Chen 2022](https://journals.lww.com/cancernursingonline/Abstract/2022/01000/Effects_of_a_Mind_Map_Based_Life_Review_Program_on.23.aspx) | 2 | 2 | 2 | 0 | 0 | 2 | 2 | 2 | 2 | 2 | 2 | 2 | 2 | 22/26 |
| Chen 2020 | 2 | 2 | 2 | 0 | 0 | 2 | 2 | 2 | 1 | 2 | 2 | 2 | 2 | 21/26 |
| [Cheung 2021](https://www.ncbi.nlm.nih.gov/pmc/articles/PMC8461121/) | 2 | 2 | 2 | 0 | 0 | 2 | 2 | 2 | 0 | 2 | 2 | 2 | 2 | 20/26 |
| [Cheung 2020](https://onlinelibrary.wiley.com/doi/10.1111/ecc.13314) | 2 | 2 | 2 | 0 | 0 | 2 | 2 | 2 | 0 | 2 | 2 | 2 | 2 | 20/26 |
| [Chung 2017](https://journals.lww.com/hnpjournal/Abstract/2017/07000/Effects_of_a_Mindfulness_Based_Stress_Reduction.7.aspx) | 0 | 0 | 2 | 0 | 0 | 2 | 0 | 2 | 0 | 2 | 2 | 2 | 2 | 14/26 |
| [Dionne-Odom 2022](https://acsjournals.onlinelibrary.wiley.com/doi/10.1002/cncr.34044) | 2 | 2 | 2 | 0 | 0 | 1 | 0 | 2 | 2 | 2 | 2 | 2 | 2 | 19/26 |
| [Fraguell-Hernando 2020](https://link.springer.com/article/10.1007/s00520-020-05322-2) | 2 | 0 | 2 | 0 | 0 | 2 | 0 | 2 | 2 | 2 | 2 | 2 | 2 | 18/26 |
| [Han 2021](https://www.ncbi.nlm.nih.gov/pmc/articles/PMC8322545/#:~:text=Conclusion%3A,of%20the%20patients%20with%20AC.) | 2 | 1 | 2 | 0 | 2 | 2 | 2 | 2 | 0 | 2 | 2 | 2 | 2 | 21/26 |
| [Huang 2021](https://journals.sagepub.com/doi/10.1177/1359105319901312?url_ver=Z39.88-2003&rfr_id=ori:rid:crossref.org&rfr_dat=cr_pub%20%200pubmed) | 2 | 0 | 2 | 0 | 0 | 2 | 1 | 2 | 1 | 0 | 2 | 2 | 2 | 16/26 |
| [Huang 2019](https://www.ncbi.nlm.nih.gov/pmc/articles/PMC6380637/) | 2 | 0 | 2 | 0 | 0 | 2 | 0 | 2 | 1 | 2 | 2 | 2 | 2 | 17/26 |
| [Julião 2013](https://www.cambridge.org/core/journals/palliative-and-supportive-care/article/abs/efficacy-of-dignity-therapy-for-depression-and-anxiety-in-terminally-ill-patients-early-results-of-a-randomized-controlled-trial/CF41530C1AF083891B23486645424E15) | 2 | 2 | 2 | 0 | 0 | 2 | 1 | 2 | 0 | 2 | 2 | 2 | 2 | 19/26 |
| [Kim 2018](https://www.ncbi.nlm.nih.gov/pmc/articles/PMC6305659/) | 2 | 1 | 2 | 0 | 0 | 2 | 0 | 2 | 1 | 2 | 2 | 2 | 2 | 18/26 |
| [Kwan 2019](https://www.sciencedirect.com/science/article/abs/pii/S0020748918302645?via%3Dihub) | 2 | 2 | 2 | 2 | 0 | 2 | 2 | 2 | 2 | 2 | 2 | 2 | 2 | 24/26 |
| [Li 2015](https://journals.sagepub.com/doi/full/10.1177/1534735419842373?rfr_dat=cr_pub++0pubmed&url_ver=Z39.88-2003&rfr_id=ori%3Arid%3Acrossref.org) | 2 | 2 | 2 | 0 | 0 | 1 | 2 | 2 | 0 | 2 | 2 | 2 | 2 | 19/26 |
| [Liao 2013](https://link.springer.com/article/10.1007/s11655-013-1593-5) | 2 | 2 | 2 | 2 | 0 | 2 | 0 | 2 | 0 | 2 | 2 | 2 | 2 | 20/26 |
| [Liossi 2001](https://onlinelibrary.wiley.com/doi/10.1002/ch.228) | 2 | 1 | 2 | 0 | 2 | 2 | 0 | 2 | 0 | 0 | 2 | 2 | 2 | 17/26 |
| [Molassiotis 2021](https://nutritionj.biomedcentral.com/articles/10.1186/s12937-020-00657-2) | 2 | 2 | 2 | 0 | 0 | 2 | 2 | 2 | 2 | 2 | 2 | 2 | 2 | 22/26 |
| [Park 2020](https://www.ingentaconnect.com/content/png/ajhb/2020/00000044/00000001/art00010;jsessionid=20md1sttr3k4g.x-ic-live-02) | 2 | 0 | 2 | 0 | 0 | 2 | 0 | 2 | 2 | 2 | 2 | 2 | 2 | 18/26 |
| [Teo 2020](https://www.jpsmjournal.com/article/S0885-3924(20)30530-3/fulltext) | 2 | 2 | 0 | 0 | 0 | 2 | 0 | 0 | 0 | 2 | 2 | 2 | 2 | 14/26 |
| [Teo 2019](https://onlinelibrary.wiley.com/doi/10.1002/pon.5275) | 2 | 2 | 2 | 0 | 0 | 2 | 0 | 2 | 0 | 2 | 2 | 2 | 2 | 18/26 |
| [Xiao 2013](https://journals.lww.com/cancernursingonline/Abstract/2013/07000/Effect_of_a_Life_Review_Program_for_Chinese.4.aspx) | 2 | 2 | 2 | 0 | 0 | 2 | 2 | 2 | 2 | 2 | 2 | 2 | 2 | 22/26 |
| [Xiao 2022](https://www.sciencedirect.com/science/article/abs/pii/S0020748922000463?via%3Dihub) | 2 | 2 | 2 | 0 | 0 | 2 | 2 | 2 | 1 | 2 | 2 | 2 | 2 | 21/26 |

**^d^** Each domain was scored a 2 if it was rated Yes, 1 if it was rated Unclear, and 0 if it was rated No, and a total score was derived by summing up the scores for all domains.

Q1) Was true randomization used for assignment of participants to treatment groups?

Q2) Was allocation to groups concealed?

Q3) Were treatment groups similar at the baseline?

Q4) Were participants blind to treatment assignment?

Q5) Were those delivering treatment blind to treatment assignment?

Q6) Were treatment groups treated identically other than the intervention of interest?

Q7) Were outcome assessors blind to treatment assignment?

Q8) Were outcomes measured in the same way for treatment groups?

Q9) Were outcomes measured in a reliable way?

Q10) Was follow-up complete and, if not, were differences between groups in terms of their follow-up adequately described and analyzed?

Q11) Were participants analyzed in the groups to which they were randomized?

Q12) Was appropriate statistical analysis used?

Q13) Was the trial design appropriate and any deviations from the standard RCT design (individual randomization, parallel groups) accounted for in the conduct and analysis of the trial?

###

### Appendix C: Report and Participant Characteristics

| **Report Characteristics** | |
| --- | --- |
| **Languages Used (n=86)^a^** | |
| Chinese | 34 |
| English | 22 |
| Spanish | 14 |
| Japanese | 9 |
| Italian | 5 |
| Danish | 4 |
| Korean | 4 |
| Dutch | 3 |
| Hindi | 3 |
| Portuguese | 3 |
| German | 2 |
| Irish | 2 |
| Thai | 2 |
| Unspecified Belgium language | 2 |
| French | 1 |
| Greek | 1 |
| Hebrew | 1 |
| Icelandic | 1 |
| Igbo | 1 |
| **Countries (n=86)** | |
| China | 20 |
| United States (U.S.) | 17 |
| Japan | 9 |
| South Korea | 4 |
| Hong Kong | 3 |
| Italy | 3 |
| Portugal | 3 |
| Spain | 3 |
| Taiwan | 3 |
| Australia and Hong Kong | 2 |
| Denmark | 2 |
| Germany | 2 |
| Singapore | 2 |
| Thailand | 2 |
| Belgium, Denmark, Ireland, Italy, The Netherlands, and the United Kingdom | 2 |
| France | 1 |
| Greece | 1 |
| Iceland | 1 |
| Israel | 1 |
| Mexico | 1 |
| Nigeria | 1 |
| Puerto Rico | 1 |
| The Netherlands | 1 |
| United States and Singapore | 1 |
| **Participant Characteristics** | |
| **Medical Conditions (n=5,054)^b^** | |
| Lung cancer | 1,123 (22.22%) |
| “End Stage/Advanced/Metastatic Cancer” | 907 (17.95%) |
| Breast cancer | 889 (17.59%) |
| Gastrointestinal cancer | 849 (16.80%) |
| Prostate cancer | 325 (6.43%) |
| Other cancer | 215 (4.25%) |
| Urological cancer | 202 (4.00%) |
| Colon cancer | 177 (3.50%) |
| Gynecological cancer | 103 (2.04%) |
| Hematological cancer | 50 (0.99%) |
| Reproductive Organ cancer | 34 (0.67%) |
| Renal cancer | 23 (0.46%) |
| Head and Neck cancer | 22 (0.44%) |
| Liver cancer | 19 (0.38%) |
| Ovarian cancer | 19 (0.38%) |
| Pancreatic cancer | 16 (0.32%) |
| Cervical cancer | 12 (0.24%) |
| Brain and soft tissue cancer | 9 (0.18%) |
| Lymphoma | 8 (0.16%) |
| Kidney cancer | 6 (0.12%) |
| Organ Failure | 6 (0.12%) |
| Skin cancer | 4 (0.08%) |
| Bladder cancer | 3 (0.06%) |
| Melanoma | 3 (0.06%) |
| Oral cancer | 3 (0.06%) |
| Biliary cancer | 2 (0.04%) |
| Bone and soft tissue cancer | 2 (0.04%) |
| Central nervous system cancer | 2 (0.04%) |
| Uterus cancer | 2 (0.04%) |
| Brain cancer | 1 (0.02%) |
| Esophageal cancer | 1 (0.02%) |
| Neuroendocrine cancer | 1 (0.02%) |
| Soft tissue cancer | 1 (0.02%) |
| Thyroid cancer | 1 (0.02%) |
| Unknown | 1 (0.02%) |
| **Race and Ethnicity of Participants with Advanced and Metastatic Cancer (n=2,242)^c^** | |
| Caucasian/White | 815 (36.63%) |
| Latino/a/x or Hispanic | 451 (20.27%) |
| African American/Black | 442 (19.87%) |
| Asian | 289 (12.89%) |
| Asian/Pacific Islander **^d^** | 151 (6.74%) |
| American Indian/Native American/Alaska Native | 31 (1.39%) |
| Other | 30 (1.35%) |
| Multiracial | 16 (0.72%) |

*Note.* **^a^**Some reports included more than the use of one language and resulted in overlapping counts. **^b^**436 study participants’ cancer type description was missing in research reports. **^c^**Only includes groups as described by 23 studies (14 U.S., 3 Portugal, 2 Singapore, 2 Spain, 1 China, 1 U.S. & Singapore) that provided race/ethnicity information. **^d^**Two reports by Patel et al. (2020) and Patel et al. (2021) reported Asians and Pacific Islanders as an aggregate group without further information.

### Appendix D: Intervention Characteristics

| **Author, Year** | **Intervention** | **Refusal/**  **Attrition Rate** | **Dosage** | **Outcome Measures** |
| --- | --- | --- | --- | --- |
| ***QUALITATIVE STUDIES*** | | | | |
| Bekelman et al., 2019^a^ | Puente para cuidar (bridge to caring) | Participation refusal rate: 41.7%.  Attrition rate: N/A. | 2-7x varied (averaged 47.5 minutes) session.  Length: unspecified. | Feasibility (defined as consensus in weekly team discussions of intervention delivery among the patient navigator, psychologist, and coinvestigators; intervention visit completion rates; and participant ratings of helpfulness), depression (PHQ-8), anxiety (GAD-7). |
| Chen et al., 2020 | Mind map-based life review program | Participation refusal rate: 47.8%.  Attrition rate: 29.8%. | 4x 45-60 minutes session.  Length: 2 weeks. | Psychological distress (DT), meaning in life (Meaning in Life Questionnaire), hope (HHI), self-transcendence (Self-transcendence Scale), satisfaction with the intervention (measured by how satisfied participants were with the MBLRP), adverse events (defined as any adverse events related to the MBLRP intervention). |
| Chen et al., 2022^a^ | WeChat-based dyadic life review program | Participation refusal rate: N/A.  Attrition rate: 2.1%. | 8x 40-60 minutes.  Length: 4 weeks. | Satisfaction with the intervention (measured by 5-point scale ranging from 1 to 5, with higher scores indicating greater satisfaction), patient QoL (QOLC-E), family caregiver QoL (QOLLTI-F), patient family adaptability and cohesion (Family Adaptability and Cohesion Evaluation Scale II), care burden of family caregivers (Zarit Caregiver Burden Interview). |
| Fink et al., 2020 | Apoyo con Cariño (Support With Caring) | Participation refusal rate: N/A.  Attrition rate: N/A. | 5x unspecified duration session.  Length: 3 months. | N/A. |
| Houmann et al., 2010^b^ | Dignity therapy | Participation refusal rate: N/A.  Attrition rate: N/A. | 2x unspecified duration session.  Length: unspecified. | Dignity (SISC, PDI), communication (SISC), social connection (SISC), suffering (SISC), depression (SISC, HADS), hopelessness (SISC), health-related quality of life (EORTC QLQ-C15-PAL), anxiety (HADS), palliative performance (PPSv2), DT patient feedback. |
| Kwan et al., 2019^a^ | Short-term life review | Participation refusal rate: 34.7%.  Attrition rate: 18.4%. | 2x 45 minutes session.  Length: 1 week. | Anxiety (HADS), depression (HADS), spiritual well-being (MQOL-HK). |
| Leng et al., 2018; Leng et al., 2019 | Meaning-centered psychotherapy (MCP-Ch) | **2018 Study** Participation refusal rate: N/A.  Attrition rate: N/A.  **2019 Study** Participation refusal rate: 42.9%.  Attrition rate: N/A. | 7x individual 90 minutes session, or 8x group 90 minutes session.  Length: unspecified. | N/A. |
| Li et al., 2014 | Dignity therapy | Participation refusal rate: 30.7% (patients), N/A (HCPs).  Attrition rate: N/A. | 1x 30 minutes session.  Length: unspecified. | N/A. |
| Lin et al., 2020 | Advanced care planning intervention | Participation refusal rate: N/A.  Attrition rate: 18.2%. | 2x unspecified duration session.  Length: unspecified. | Feasibility and process of delivery (measured by a study fidelity checklist, reviews of medical records and note-taking). |
| Lin et al., 2022 | Dignity therapy | Participation refusal rate: N/A.  Attrition rate: N/A. | Unspecified number of sessions and length. | N/A. |
| Liossi et al., 2001^a^ | Hypnosis | Participation refusal rate: N/A.  Attrition rate: N/A. | 4x 30 minutes session.  Length: 4 weeks. | QoL (RSCL), anxiety (HADS), depression (HADS). |
| Nunziante et al., 2021 | Dignity therapy | Participation refusal rate: 26.0%.  Attrition rate: 24.3%. | Unspecified number of sessions.  Length: 31 days. | Feasibility and acceptability (defined as the enrollment and retention rates, the duration of each DT session, and the timeframes concerning each study step), dignity-related distress (PDI), patient's opinion on DT intervention (DT Patient Feedback Questionnaire). |
| Ólafsdóttir et al., 2018 | Advanced care planning intervention | Participation refusal rate: N/A.  Attrition rate: 41.7%. | 3x 1 hour session.  Length: 12-18 weeks. | N/A. |
| Patel et al., 2019 | Lay health workers educate engage and encourage patients to share (LEAPS) cancer care | Participation refusal rate: N/A.  Attrition rate: N/A. | Unspecified number of sessions.  Length: 12 months. | N/A. |
| Pon, 2010 | My Wonderful Life Board Game | Participation refusal rate: N/A.  Attrition rate: N/A. | 3x unspecified duration session.  Length: unspecified. | N/A. |
| Schulman-Green et al., 2022 | Managing Cancer Care | Participation refusal rate: N/A.  Attrition rate: N/A. | Unspecified number of sessions and length. | N/A. |
| Takenouchi et al., 2022 | Lifeline Interview Method | Participation refusal rate: N/A.  Attrition rate: 9.1%. | 1x unspecified duration (average 47.7 minutes) session.  Length: unspecified. | N/A. |
| Torres-Blasco et al., 2022 | Caregivers-Patients Support to Latinx coping advanced-cancer (CASA) | Participation refusal rate: N/A.  Attrition rate: N/A. | 4x 45-60 minutes session.  Length: 4-8 weeks. | N/A. |
| van den Hurk et al., 2015^a^ | Mindfulness-based stress reduction | Participation refusal rate: N/A.  Attrition rate: 51.4%. | 8x 2.5 hours session.  Length: 8 weeks. | Psychological distress (HADS), QoL (EORTC QLQ-LC13), psychological stress reaction (IES), worry (PSWQ), lapse of attention/awareness (MAAS), caregiver appraisal (SPPIC), care-derived self-esteem (CRA-SE). |
| Wang et al., 2019^a^ | Family participatory dignity therapy | Participation refusal rate: 40.9%.  Attrition rate: 23.1%. | 4x 45-60 minutes session.  Length: unspecified. | Hope (HHI), spiritual well-being (FACIT-Sp), QoL (EORTC QLQ-C30), acceptability (measured by “How satisfied are you with the services you received in this program?” and “How valuable was this program to you?”). |
| Xiao et al., 2012 | Life review programme | Participation refusal rate: N/A.  Attrition rate: 30.0%. | 3x unspecified duration session.  Length: 3 weeks. | N/A. |
| Yang et al., 2021 | Educate, Nurture, Advise, Before Life Ends (ENABLE) | Participation refusal rate: N/A.  Attrition rate: N/A. | 6x 1 hour session.  Length: 6 weeks. | N/A. |
| ***RANDOMIZED CONTROL STUDIES*** | | | | |
| Anderson et al., 2004 ^b^ | Pain education | Participation refusal rate: 66.8%.  Attrition rate: approximately 33.3%. | 1x 110 minutes session.  Length: 1 day. | Pain intensity (BPI), pain-related interference (BPI), functional status (ECOG Performance Status Scale), QoL (SF-12), perceived control of pain (SOPA - Pain Control Scale), adherence to analgesic prescriptions (measured by research nurse recording patient adherence). |
| Ando et al., 2010 | Short-term life review | Participation refusal rate: 4.9%.  Attrition rate: 11.7%. | 2x 30-60 minutes session.  Length: 2 weeks. | Sense of meaning (FACIT-Sp), anxiety (HADS), depression (HADS), patient-perceived good death (Good Death Inventory), intensity of psychological suffering (measured by a numeric scale), intensity of pain and physical symptoms (NRS), performance status (ECOG-PSR). |
| Bakitas et al., 2009^b^ | Educate, Nurture, Advise, Before Life Ends (ENABLE II) | Participation refusal rate: 52.0%.  Attrition rate: N/A. | 4x unspecified duration session.  Length: 4 weeks. | Functional status (KPS), symptom intensity (ESAS), mood status (CES-D), QoL (FACIT-Pal). |
| Bouchard et al., 2019^b^ | Cognitive behavioral stress management | Participation refusal rate: 4.1%.  Attrition rate: 19.8%. | 10x 90 minutes session.  Length: 10 weeks. | Medical comorbidities (patient reported and combined using weighting scheme from Charlson Comorbidity Index), acceptability (participant rate confidence in using each session’s information/skills and to report on group dynamics), efficacy (MAX-PC). |
| Caruso et al., 2020^b^ | Managing Cancer and Living Meaningfully (CALM) | Participation refusal rate: N/A.  Attrition rate: 50.0%. | 12x 45-60 minutes session.  Length: 6 months. | Depression (PHQ-9), anxiety symptoms (GAD-7), death anxiety (DADDS), post-traumatic growth (PTGI), spirituality and meaning (FACIT-Sp), experiences in close relationships (ECR-M-16), quality of life at the end of life (QUAL-EC). |
| Chen et al., 2020; Chen et al., 2022 | Mind map-based life review program | Participation refusal rate: 47.8%.  Attrition rate: 29.8%. | 4x 45-60 minutes session.  Length: 2 weeks. | 2020 Study: Psychological distress (DT), meaning in life (Meaning in Life Questionnaire), hope (HHI), self-transcendence (Self-transcendence Scale), satisfaction with the intervention (measured by how satisfied participants were with the MBLRP), adverse events (defined as any adverse events related to the MBLRP intervention).  2022 Study: Anxiety symptoms (SAS), depressive symptoms (SDS). |
| Cheung et al., 2020^b^ | Self-administered acupressure | Participation refusal rate: 65.5%.  Attrition rate: 20.0%. | 2x 2 hours session and 3x 1 hour visit.  Length: 4 weeks. | Fatigue levels (BFI-C), sleep disturbance levels (PSQI), pain intensity, severity of psychological distress (HADS), objective sleep quality (measured by actigraphy), HQOL (FACT-G), satisfaction with the intervention (measured by a 13 questions satisfaction questionnaire). |
| Cheung et al., 2021 | Aerobic exercise & Tai-chi | Participation refusal rate: 81.6%.  Attrition rate: 46.7%. | 24x 60 minutes session.  Length: 12 weeks. | Feasibility (measured by intervention completion, exercise adherence, and adverse events), sleep quality (PSQI), psychological distress (HADS), fatigue (BFI), quality of life (EORTC QLQ-C30, QLQ-LC13), physical performance (measured by a walk test, timed up-and-go, a sit-to-stand test, and a 1-leg standing test), physical activity levels (measured by actigraphy), objective sleep measures (measured by actigraphy), circadian rhythms (measured by cortisol in the saliva). |
| Dionne-Odom et al., 2021 | ENABLE (Educate, Nurture, Advise, Before Life Ends) Cornerstone | Participation refusal rate: 16.4% (patients) and 51.9% (caregivers).  Attrition rate: 40.0% (patients) and 44.4% (caregivers). | 6x 20-60 minutes session.  Length: 6 weeks. | Feasibility (defined as completion rates of data collection and intervention session completion), acceptability (measured by how likely participants would be to recommend the program overall to someone else in a similar circumstance), distress (HADS), patient QoL (FACIT-Pal), caregiver QoL (Caregiver Quality of Life-Cancer assessment). |
| Du et al., 2022^b^ | Heart to Heart Card Game | Participation refusal rate: 4.3%.  Attrition rate: 30.3%. | 1x 50-90 minutes session.  Length: 1 day. | Activities of daily living (MBI), dignity (PDI), psychological distress (NCCN Distress Thermometer), QoL (EORTC-QLQ-C30). |
| Fischer et al., 2018^b^ | Apoyo con Cariño (Support With Caring) | Participation refusal rate: 21.5%.  Attrition rate: 20.6%. | 5x unspecified duration session.  Length: 3 months. | ACP (defined as electronic health record documentation of Medical Durable Power of Attorney, study-specific advance directive, or other type of comprehensive AD), pain (BPI), QoL (MQOL), hospice use and length of stay, aggressiveness of care at the end of life, process measures of the patient navigator intervention. |
| Fraguell-Hernando et al., 2020 | Individual Meaning-Centered Psychotherapy-Palliative Care | Participation refusal rate: N/A.  Attrition rate: 37.3%. | 3x 45-60 minutes session.  Length: 4 weeks. | Anxiety (HADS), depression (HADS), emotional distress (DED), demoralization (DS-II). |
| Gil et al., 2018^b^ | Meaning-Centered Psychotherapy-compassionate palliative care | Participation refusal rate: N/A.  Attrition rate: 41.2%. | 3x 30 minutes session.  Length: unspecified. | Patient satisfaction (measured by a patient satisfaction questionnaire). |
| Han et al., 2021 | Naikan and Morita Therapies | Participation refusal rate: 9.6%.  Attrition rate: 33.9%. | 20x 2 hours Naikan Therapy session, unspecified number of Morita Therapy sessions.  Length: 7 weeks. | Distress (DT, DT problem list), posttraumatic growth (PTGI). |
| Huang et al., 2019; Huang et al., 2021 | Magnanimous therapy | **2019 Study** Participation refusal rate: N/A.  Attrition rate: 100.0% (GCMT, none alive), 90.0% (ICMT), 94.0% (control).  **2021 Study** Participation refusal rate: N/A.  Attrition rate: N/A. | 8x 40 minutes session.  Length: 2 weeks. | 2019 Study: Psychological coping (CCMQ), psychological adjustment (PASCP), living function (FLIC), and survival rate (measured by telephone interviews).  2021 Study: Psychosomatic state (PSSCP) ^a^, anxious and depressive symptoms (HADS), immune functions (defined as levels of IgA, IgG, IgM, and NK (CD16+CD56+) cells). |
| Julião et al., 2013; Julião et al., 2014; Julião et al., 2017^b^ | Dignity therapy | **2013 Study** Participation refusal rate: 4.8%.  Attrition rate: 50.0%.  **2014 Study** Participation refusal rate: 4.8%.  Attrition rate: 55.0%.  **2017 Study** Participation refusal rate: 4.8%.  Attrition rate: 12.5%. | 1x 30-60 minutes session and final session.  Length: 4-6 days post consent and baseline assessment. | 2013 Study: Depression (HADS), anxiety (HADS).  2014 Study: Depression (HADS), anxiety (HADS).  2017 Study: Demoralization syndrome (defined as a diagnosis if satisfy all 5 criteria), desire for death (DDRS), sense of dignity (PDI). |
| Kim et al., 2018 | ILOVEBREAST mobile game | Participation refusal rate: N/A.  Attrition rate: 5.3%. | 9x 30 minutes session.  Length: 3 weeks. | Time spent for education (measured as either the time spent for game playing or self-education using the brochure with preventive measures), medication compliance (K-MARS), physical side effects (measured by a 5-point Likert scale), psychological side effects (BDI, Spielberger State-Trait Anxiety Scale, WHOQOL-BREF), levels of satisfaction with the game (measured by a 8-questions self-reported survey). |
| Li et al., 2019^b^ | Wellness education | Participation refusal rate: 12.6%.  Attrition rate: 13.2%. | 6x 45 minutes session.  Length: 8 weeks. | Feasibility (defined as feasibility of participant recruitment, intervention provision, and data collection), QoL (FACT-L, CQOLC), changes in way of life/family relationships (FES), anxiety (HADS), depression (HADS). |
| Liao et al., 2013 | Chinese Medicine five-element music | Participation refusal rate: N/A.  Attrition rate: 8.8%. | 15x 30 minutes session.  Length: 3 weeks. | QoL (HQOLI-R), performance status (KPS), symptoms (Symptom diary scores). |
| Maungtoug et al., 2021^b^ | Ritualized chanting in palliative care | Participation refusal rate: 0%.  Attrition rate: 15.0%. | Unspecified number of 10 minutes session.  Length: 6 weeks. | Comfort levels of the participants (EOLPQ). |
| Molassiotis et al., 2021 | Patient- and family-centered psychosocial-based nutrition intervention PIcNIC & PiCNIC2 | Participation refusal rate: 52.2 % (Australia) and 38.2% (Hong Kong).  Attrition rate: 43.8% (Australia) and 47.6% (Hong Kong). | 1x 1-1.5 hours and 2x 30 minutes session.  Length: 4 weeks. | Feasibility (defined as recruitment rate, consent rate (patients), retention rate (patients), proportion of patients with available caregivers, consent rate (caregivers), retention rate (carers), adherence to the protocol, and acceptability of assessment tools), QoL (FAACT), nutritional status (PG-SGA-SF), anxiety and depression (HADS), patient eating-related distress (MQOL, SAS), self-efficacy (CaSES), caregiver distress (Caregiver Distress Checklist), caregiver eating-relate distress (Eating-related Distress Scale). |
| Onyechi et al., 2016^b^ | Rational emotive hospice care therapy | Participation refusal rate: 46.8%.  Attrition rate: 0%. | 10x 45 minutes session.  Length: 10 weeks. | Death anxiety (DAQ), psychological distress (K_10_), patients’ and family caregivers' level of problematic assumptions (CPFCAQ). |
| Park et al., 2020 | Lifestyle intervention | Participation refusal rate: 0%.  Attrition rate: 0%. | 24x unspecified duration session.  Length: 8 weeks. | QoL (EORTC QLQ-C30), anxiety (HADS), depression, life satisfaction (SWLS), physical activity (IPAQ, measured by a waist-worn accelerometer), sedentary behavior (IPAQ, measured by a waist-worn accelerometer). |
| Patel et al., 2020^b^ | Lay health workers educate engage and encourage patients to share (LEAPS) cancer care | Participation refusal rate: N/A.  Attrition rate: N/A. | Unspecified number of sessions.  Length: 12 months. | QoL (FACT-G7), patient activation (PAM), patient satisfaction with decision (SWD), symptom burden (ESAS), healthcare use and total costs of care (defined as all healthcare use and total costs of care obtained via claims data from Unite Here Health), goals of care and advance directives (defined as documentation and dates of goals of care and advance directives from Unite Here Health Electronic Health Record), fidelity (defined as Unite Here Health’s established processes to assess fidelity of interventions). |
| Quílez-Bielsa et al., 2022^b^ | Meaning-centered psychotherapy-essential care | Participation refusal rate: 18.9%.  Attrition rate: 6.7%. | 4x 60 minutes session.  Length: 4 weeks. | QoL (EQ-5D-3L), spiritual well-being (FACIT-Sp), anxiety (HADS), depression (HADS), self-compassion (SCS-SF), hopelessness (HAI), demoralization (DM), distress (DT), acceptability (measured by percentage of patients who complete the four sessions, percentage of patients who answer all the questionnaires under study, Post-Therapy Assessment Questionnaire). |
| Teo et al., 2019^c^ | Cognitive behavioral therapy symptom management combined with acceptance and commitment therapy mindfulness and values-guided principles | Participation refusal rate: 73.5%.  Attrition rate: 17.2%. | 4x 1 hour session.  Length: 8 weeks. | Acceptability (Client Satisfaction Questionnaire, Cultural Sensitivity Assessment Tool), engagement (measured by participants rating on how many days in the last week they had practiced mindfulness), distress (HADS), pain severity (BPI), disability (Pain Disability Index), fatigue (PROMIS F-SF). |
| Teo et al., 2020 | Cognitive behavioral therapy-based intervention | Participation refusal rate: 78.8%.  Attrition rate: 25.0%. | 4x 1 hour session.  Length: 8 weeks. | Feasibility (measured by ability to recruit participants within the study period and the proportion of intervention sessions the trial participants attended), intervention acceptability (Client Satisfaction Questionnaire), psychological distress (HADS), self-efficacy (Cancer Behavior Inventory Version 2). |
| Xiao et al., 2013 | Life review programme | Participation refusal rate: N/A.  Attrition rate: 30.0%. | 3x unspecified duration session.  Length: 3 weeks. | Overall QoL (self-report single-item scale for overall QoL), QoL concerns (Quality-of-Life Concerns in the End-of-Life Questionnaire). |
| Xiao et al., 2022 | Family-oriented dignity therapy | Participation refusal rate: 29.8%.  Attrition rate: 44.2%. | 3x unspecified duration session.  Length: 4 weeks. | Dignity-related distress (PDI), depressive symptoms (PHQ-9), spiritual well-being (FACIT-Sp), satisfaction level with intervention (Satisfaction with Therapy and Therapist Scale). |
| Yanez et al., 2015^b^ | Cognitive behavioral stress management | Participation refusal rate: 69.2%.  Attrition rate: 17.6%. | 10x 90 minutes session.  Length: 10 weeks. | Feasibility (measured by study recruitment, retention, and attendance rates), acceptability (measured by weekly session evaluations asking participants to rate their confidence in using the information presented in the session), cancer-related distress (IES-R), depressive symptoms (PROMIS cancer depression item bank CAT), HRQOL (FACT-G), self-efficacy in stress management skills (Measure of Current Status), satisfaction of the intervention (measured weekly via study website). |
| Ye et al., 2017^b^ | Be Resilient to Breast Cancer program | Participation refusal rate: 25.7%.  Attrition rate: 81.9%. | 52x 120 minutes session.  Length: 12 months. | 3 and 5 year cancer-specific survival, anxiety (HADS), depression (HADS), QoL (EORTC QLQ-C30), resilience (CD-RISC-10), physical allostatic load (measured by body mass index), waist-hip ratio, resting pulse, the standard deviation of R-R intervals, heartbeat to heartbeat), resting systolic and diastolic blood pressure (SBP and DBP, white blood cell count (WBC), red blood cell count (RBC), hemoglobin, serotonin, hormone cortisol (HC), C-reactive protein (CRP), interleukin-6 (IL-6) and Cluster of Differentiation 4/Cluster of Differentiation 8(CD4^+^/CD8^+^)). |
| Zheng et al., 2022^b^ | Very important person (VIP) for future care | Participation refusal rate: N/A.  Attrition rate: 3.1%. | 2x unspecified duration intake session, 1x 4 minutes session, and 2x 40 minutes session.  Length: 5 days. | Intention of terminal treatment (hospice treatment intention questionnaire), QoL (modified QOLC-E), certainty of patients' decision-making (patient decision certainty the “Sure-test” decisional conflict scale). |
| ***QUASI-EXPERIMENTAL STUDIES*** | | | | |
| Catania et al., 2021 | INtervention FOcused on quality of life assessment (INFO-QoL) | Participation refusal rate: 27.8%.  Attrition rate: 47.6%. | 3x unspecified duration educational program session (total lasted 4 hours), unspecified number of 10-15 minutes QoL education session for patients and their families, and unspecified number and duration of assessment session.  Length: unspecified. | Feasibility (measured by 1) timing to educate healthcare professionals, 2) fidelity through a checklist, 3) team members’ competence and confidence level in delivering the intervention across three time points, 4) patient recruitment and dropout rates), acceptability (measured by overall rate of eligible patients who accepted to participate in the study and the relevance, appropriateness, and usefulness of the intervention from team members perspective), QoL (IPOS), patient management (collected using a composite patient management score on management actions including medications, interventions, nutrition and diet, vital signs, diagnostic tests, referrals and consultations, patient and family counseling, and education). |
| Chimluang et al., 2017^b^ | Intervention based on basic Buddhist principles | Participation refusal rate: N/A.  Attrition rate: N/A. | 3x 2-3 hours session.  Length: 3 days. | Spiritual well-being (SWBS). |
| Ichihara et al., 2019 | Spiritual care using Spiritual Pain Assessment Sheet (SpiPas) | Participation refusal rate: 14.8%.  Attrition rate: 50.0%. | Unspecified number of sessions and length. | Spiritual well-being (FACIT-Sp), anxiety (HADS), depression (HADS), QoL (CoQoL). |
| Landa-Ramírez et al., 2020^b^ | Cognitive behavioral therapy | Participation refusal rate: N/A.  Attrition rate: 44.4%. | 4-6x unspecified duration session.  Length: 3 weeks. | Depression (HADS), anxiety (HADS), leisure activities (Activities, Thoughts and Mood Diary), thoughts related to depression, anxiety, and cancer (Activities, Thoughts and Mood Diary), mood (Activities, Thoughts and Mood Diary). |
| Lee et al, 2017^b^ | Mindfulness-based stress reduction program | Participation refusal rate: 50.0%.  Attrition rate: 43.8%. | 8x 2 hours session.  Length: 8 weeks. | Pain (Wisconsin Brief Pain Inventory), heart rate variability (measured by using BFM 5000), anxiety (HADS), depression (HADS), distress (DT), QoL (FACT-B). |
| Li et al., 2020^b^ | Dignity therapy | Participation refusal rate: 47.4%.  Attrition rate: 16.7%. | 1x 30-60 minutes session.  Length: 1 day. | Life distress (PDI-MV), psychological distress (PDI-MV), demoralization (DS-MV), depression (PHQ-9). |
| Patel et al., 2021^b^ | Lay health workers educate engage and encourage patients to share (LEAPS) cancer care | Participation refusal rate: N/A.  Attrition rate: N/A. | Unspecified number of sessions.  Length: 12 months. | Goals of care (defined as documented goals of care in a clinical note in the electronic health record), HRQOL (FACT-G), advance directive documentation, health care use (measured by palliative care, hospice use, and dates of death), clinical trial participation. |
| Zhang et al., 2019 | WeChat-based life review programme | Participation refusal rate: 61.5%.  Attrition rate: 6.5%. | 6x 40-60 minutes session.  Length: 6 weeks. | Anxiety (SAS), depression (SDS), self-transcendence (STS), meaning in life (Meaning in Life Questionnaire), hope (HHI). |
| ***SINGLE ARM TRIALS*** | | | | |
| Ando et al., 2008 | Short-term life review | Participation refusal rate: N/A.  Attrition rate: 0%. | 2x 30-60 minutes session.  Length: 2 weeks. | Spiritual well-being (FACIT-Sp), anxiety (HADS), depression (HADS), suffering (measured by a numeric rating scale), happiness (measured by a numeric rating scale). |
| Ando et al., 2016 | Mindfulness art therapy | Participation refusal rate: N/A.  Attrition rate: 0%. | 2x 1 hour session.  Length: 2 weeks. | Tension-anxiety (POMS), depression-dejection (POMS), anger-hostility (POMS), vigor-activity (POMS), fatigue-inertia (POMS), confusion-bewilderment (POMS), overall mood (POMS), spiritual well-being (FACIT-Sp). |
| Delrieu et al., 2020 | Physical activity program | Participation refusal rate: 5.6%.  Attrition rate: 2.0%. | Unspecified number of sessions.  Length: 6 months. | Feasibility (defined as proportion of participants achieving the international physical activity recommendations), physical activity (IPAQ), sedentary activities (IPAQ), physical fitness (measured by a 6-minute walk test, oxygen uptake consumption and heart rate recordings), anthropometrics (measured by standing height in centimeters, body weight in kilograms, waist and hip circumferences, and body mass index), QoL (EORTC QLQ-C30), fatigue (EORTC QLQ-C30, Piper Scale), social deprivation (EPICES), tumor progression rate (estimated by Kaplan-Meier analysis), overall survival (estimated by Kaplan-Meier analysis). |
| Houmann et al., 2014 ^b^ | Dignity therapy | Participation refusal rate: 70.4%.  Attrition rate: 69.3%. | Unspecified number of sessions and length. | Dignity (SISC, PDI), communication (SISC), social connection (SISC), suffering (SISC), depression (SISC, HADS), hopelessness (SISC), health-related quality of life (EORTC QLQ-C15-PAL), anxiety (HADS), palliative performance (PPSv2), DT patient feedback. |
| Kang et al., 2015 | Meaning of My Life | Participation refusal rate: N/A.  Attrition rate: 52.0%. | 5x 30-40 minutes session.  Length: 5 days. | Meaning of life (Meaning in Life for Adolescents Questionnaire), QoL (SBQOL). |
| Li et al., 2015 | Caring for couples coping with cancer '(4Cs)' program | Participation refusal rate: 13.3%.  Attrition rate: 21.4%. | 6x unspecified duration session.  Length: 6 weeks. | Self-efficacy (CBI-B), coping strategies (DCI), communication (CRCP), physical and mental health (SF-12), negative emotions (HADS), positive emotions (BFS), marital satisfaction (RDAS). |
| Nakayama et al., 2009 ^b^ | Music therapy | Participation refusal rate: N/A.  Attrition rate: N/A. | Unspecified number of 40 minutes session.  Length: unspecified. | Stress (measured by salivary cortisol), mood (Mood Inventory). |
| Niki et al., 2019 | Virtual reality | Participation refusal rate: N/A.  Attrition rate: N/A. | 1x 30 minutes session.  Length: 1 day. | Symptoms (ESAS), side effects (NRS), level of fun and happiness (NRS), pre-VR travel expectation and post-VR travel satisfaction (NRS). |
| Ramos et al., 2018^b^ | Life program | Participation refusal rate: 25.0%.  Attrition rate: 56.4%. | 6-8x 60-90 minutes session.  Length: 6-8 weeks. | Depression (DASS-21), anxiety (DASS-21), stress (DASS-21), psychological flexibility (AAQ-II), mindfulness (TMS), self-compassion (SCS-SF), satisfaction with intervention (measured by feedback and satisfaction questionnaire). |
| Sakaguchi et al., 2015 ^b^ | Collage Activity Based on Life Review | Participation refusal rate: 13.3%.  Attrition rate: 8.3%. | 2x 1 hour session.  Length: 2 weeks. | Activities of daily living (Barthel Index), QoL (FACIT–Sp), anxiety (HADS), depression (HADS), self-efficacy (SESTC). |
| Warth et al., 2018 | Song of Life | Participation refusal rate: N/A.  Attrition rate: 13.3%. | 2x unspecified duration session.  Length: unspecified. | Life closure (LCS), well-being (VAS), acute pain (VAS), relaxation (VAS), worry (VAS). |
| ***FEASIBILITY/ACCEPTABILITY TRIALS*** | | | | |
| Hanson et al., 2013 | Circles of Care | Participation refusal rate: N/A.  Attrition rate: N/A (patients) and 46.1% (volunteers). | Unspecified number of sessions and length. | Reach (defined as the number of lay health advisors completed training and participated in at least one follow-up activity; numbers and characteristics of support team members trained and engaged in support and the numbers of individual supported). Adoption (defined as the numbers and types of recruitment contacts with organizations and frequency with which each type of organization yielded study participants), implementation (measured by a survey asking a) how many people they supported, b) where they found these individuals, and c) what type of information or support they provided to them; and parallel written surveys for support team members offering support and persons with serious illness seeking support). |
| Molassiotis et al., 2018^c^ | Patient- and family-centered psychosocial-based nutrition intervention PIcNIC & PiCNIC2 | Participation refusal rate: 76.8% (Australia) and 43.4% (Hong Kong).  Attrition rate: 34.8% (Australia) and 30.0% (Hong Kong). | Unspecified number of sessions.  Length: 5-7 days (Australia) and 4 weeks (Hong Kong). | Feasibility (defined as eligibility, recruitment, retention rates, and fidelity of intervention delivery), acceptability (measured by surveys of patients’, families’ and HCPs’ perceptions of and satisfaction with the intervention), energy/protein intake (calculation based on Liu’s equation of basal metabolic rate x activity factor specific for Chinese patients). |
| ***PROTOCOLS*** | | | | |
| Costas-Muñiz et al., ongoing study | Meaning-centered psychotherapy for Latinos | Participation refusal rate: N/A.  Attrition rate: N/A. | 7x 60 minutes session.  Length: 7-14 weeks. | Spiritual well-being (FACIT-Sp), depression (HADS), anxiety (HADS), hopelessness (BHS), QoL (FACIT-Sp). |
| Matthys et al., 2021 | Face-to-face FOCUS+ & Web-based iFOCUS | Participation refusal rate: 45.0% (expected).  Attrition rate: 35.0% (expected). | FOCUS+  2x 90 minutes home visit and 1x 30 minutes session.  Length: 12 weeks.    iFOCUS  4x unspecified duration session.  Length: 12 weeks. | Emotional functioning (EF-10), self-efficacy (Lewis Cancer self-efficacy scale), benefits of illness (Benefits of Illness Scale), coping (Brief Cope), dyad communication (Ways of giving support questionnaire - Active engagement scale), patient QoL (EORTC QLQ-C15-PAL, EORTC QLQ-C30), caregiver QoL (CQOLC), health economic measures (EQ-5D-5L, CSRI). |
| Miyamoto et al., 2022 | Managing Cancer and Living Meaningfully (CALM) | Participation refusal rate: N/A.  Attrition rate: N/A. | 3-6x 45-60 minutes session.  Length: 3-6 months. | Depressive symptoms (PHQ-9), QoL (QUAL-EC), attachment insecurities (ECR-M16), death anxiety (DADDS), extent to which patients feel supported by their CALM therapist (CEQ). |
| Scheffold et al., 2015 | Managing Cancer and Living Meaningfully (CALM) | Participation refusal rate: N/A.  Attrition rate: N/A. | 8x 50 minutes session.  Length: 6 months. | Orientation, memory and concentration (SOMC), major depressive disorder (SCID-I), psychological distress (DT), depression (PHQ-9, BDI-II), generalized anxiety disorder (GAD-7), demoralization (DS), severity of fatigue (BFI), physical problems (MSAS-SF), spiritual well-being (FACIT-Sp), death and dying distress (DADDS), posttraumatic growth (PTGI), experiences in close relationships (ECR-M16), QoL (QUAL-EC), communication (CCS), conflict solution (CCS), relationship satisfaction in romantic relationships (CCS), perceived helpfulness of a therapeutic session (Clinical Evaluation Questionnaire). |
| Torres-Blasco et al., 2022 | Caregivers-Patients Support to Latinx coping advanced-cancer (CASA) | Participation refusal rate: N/A.  Attrition rate: N/A. | 4x 45-60 minutes session.  Length: 4-8 weeks. | N/A. |
| van der Wel et al., 2022 | Family, outlook, coping, uncertainty, symptom management (FOCUS+) | Participation refusal rate: N/A.  Attrition rate: N/A. | 3x unspecified duration session.  Length: unspecified. | N/A. |
| Zhang et al., 2018 | WeChat-based life review programme | Participation refusal rate: N/A.  Attrition rate: N/A. | 6x 40-60 minutes session.  Length: 6 weeks. | Anxiety (SAS), depression (SDS), self-transcendence (STS), meaning in life (Meaning in Life Questionnaire), hope (HHI). |

***Note.*** ^a^Included in meta-analysis. ^b^Excluded from meta-analysis due to insufficient data. ^c^Only included Asian sample data in meta-analysis.

*AAQ-II* Acceptance and Action Questionnaire-II, *BDI-II* Beck Depression-Inventory II, *BFI* Brief Fatigue Inventory, *BFI-C* Brief Fatigue Inventory Chinese version, *BFS* Benefit-Finding Scale, *BHS* Beck Hopelessness Scale, *BPI* Brief Pain Inventory, *CaSES* Caregiver Self-efficacy Scale, *CBI-B* Cancer Behavior Inventory, *CCMQ* Cancer Coping Modes Questionnaire, *CCS* Couple Communication Scale, *CD-RISC-10* Conner-Davison Resilience Scale, CEQ Clinical Evaluation Questionnaire, *CES-D* Center for Epidemiological Study – Depression Scale, *CoQoL* Comprehensive Quality of Life Outcome, *CPFCAQ* Cancer Patients’ and Family Caregivers’ Assumptions Questionnaire, *CQOLC* Caregiver QOL Index– Cancer Scale, CRA-SE Caregiver Reaction Assessment, *CRCP* Cancer-Related Communication Problems, *CSRI* Client Socio-Demographic and Service Recipient Inventory, *DADDS* Death and Dying Distress Scale, *DAQ* Death Anxiety Questionnaire, *DASS-21* Depression, Anxiety, and Stress Scales, *DCI* Dyadic Coping Inventory, *DDRS* Desire for Death Rating Scale, *DED* Detection of Emotional Distress, *DM* Demoralization Scale, *DS-MV* Demoralization Scale Mandarin Version, *DS-II* Short Demoralization Scale, *DT* Distress Thermometer, *DT Patient Feedback Questionnaire* Dignity Therapy Patient Feedback Questionnaire, *DWI* Dealing with Illness Inventory, *ECOG-PSR* Eastern Cooperative Oncology Group Performance Status Rating, *ECOG Performance Status Scale* Eastern Cooperative Oncology Group Performance Status Scale, *ECR-M-16* Experiences in Close Relationships Inventory – Modified Short Form Version, *EF-10* European Organisation for Research and Treatment of Cancer Emotional Functioning Short Form, EOLPQ End of Life Planning Questionnaire, *EORTC QLQ-C15-PAL* European Organisation for Research and Treatment of Cancer Quality of Life Questionnaire - PALliative Cancer Care, *EORTC QLQ-C30* European Organisation for Research and Treatment of Cancer Core Quality of Life Questionnaire, *EPICES* Evaluation of Precarity and Inequalities in Health Examination Centers Questionnaire, *EQ-5D-3L* 3-level version of EQ-5D, *ESAS* Edmonton Symptom Assessment Scale, *FAACT* Functional Assessment of Anorexia/Cachexia Therapy, *FACIT-Sp* Functional Assessment of Chronic Illness Therapy – Spiritual Well-Being Scale, *FACT-B* Functional Assessment of Cancer Therapy Scales for Breast, *FACT-G* Functional Assessment of Cancer Therapy–General, *FACT-L* Functional Assessment of Cancer Therapy–Lung, *FACT-G7* Functional Assessment of Cancer Therapy – General 7, *FACIT-Pal* Functional Assessment of Chronic Illness Therapy – Palliative Care*, FES* Family Environment Scale, *FLIC* Functional Living Index-Cancer, *GAD-7* Generalized Anxiety Disorder-7, *HADS* Hospital Anxiety and Depression Scale, *HAI* Hopelessness Assessment in Illness Scale, *HHI* Herth Hope Index, *HQOLI-R* Hospice Quality of Life Index-Revised, *IES-R* Impact of Event Scale–Revised, *IPAQ* International Physical Activity Questionnaire, *IPOS* Integrated Palliative care Outcome Scale, *K-MARS* Medication Adherence Rating Scale Korean version, *KPS* Karnofsky Performance Scale, *K_10_* Kessler Psychological Distress Scale, *LCS* Life Closure Scale, *MAAS* Mindful Attention and Awareness Scale, *MAX-PC* Memorial Anxiety Scale for Prostate Cancer, *MBI* Modified Barthel index, *MQOL* McGill Quality of Life Questionnaire, *MQOL-HK* McGill Quality of Life Questionnaire Hong Kong Chinese, *MSAS-SF* Memorial Symptom Assessment Scale Short Form, *NCCN Distress Thermometer and Problem List* National Comprehensive Cancer Network Distress Thermometer and Problem List, *NRS* 11-point Numerical Rating Scale, *PAM* Patient Activation Measure, *PASCP* Psychological Adjustment Scale for Cancer Patients, *PDI* Patient Dignity Inventory, *PDI-MV* Patient Dignity Inventory Mandarin Version, *PG-SGA-SF* Patient-Generated Subjective Global Assessment Short Form, *PHQ-8* Patient Health Questionnaire-8, *PHQ-9* Patient Health Questionnaire-9, *POMS* Profile of Mood States, *PROMIS F-SF* PROMIS Fatigue‐Short Form, *PSQI* Pittsburgh Sleep Quality Index, *PSWQ* Penn State Worry Questionnaire, *PTGI* Posttraumatic Growth Inventory, *QLQ-LC13* European Organization for Research and Treatment of Cancer Quality of Life Questionnaire Lung Cancer Module, *QOLC-E* Quality-of-Life Concerns in the End of Life Questionnaire, *QOLLTI-F* Quality of life in Life-threatening Illness-Family Carer Questionnaire, *QUAL-EC* Quality of Life at the End of Life – Cancer Scale, *RDAS* Revised Dyadic Adjustment Scale, *RSCL* Rotterdam Symptom Checklist, *SAS* Symptom Assessment Scale, *SAS* Zung Self-rating Anxiety Scale, *SBQOL* SmithKline Beecham Quality of Life Scale, *SCID-I* Structured Clinical Interview for DSM-IV, *SCS-SF* Self-Compassion Scale-Short Form, *SDS* Zung Self-rating Depression Scale, *SESTC* Self-Efficacy Scale for Terminal Cancer, *SF-12* Physical and Mental Health Summary Scales of the Short Form (SF) -12 Health Survey, *SISC* Structured Interview for Symptoms and Concerns, *SOMC* Short Orientation-Memory-Concentration, *SOPA - Pain Control Scale* Survey of Pain Attitudes – Pain Control Scale, *SPPIC* Self-Perceived Pressure from Informal Care, *STS* Self-Transcendence Scale, *SWBS* Spiritual Well-being Scale, *SWD* Satisfaction with Decision Scale, *SWLS* Satisfaction with Life Scale, *TMS* Toronto Mindfulness Scale, *VAS* Visual Analogue Scales, *WHOQOL-BREF* World Health Organization Quality of Life-BREF Scale, *ZBI* Zarit Burden Interview.

*No full measure name for PSSCP.

###

### Appendix E: Cultural Considerations/Adaptations of Studies

| **Author, Year** | **Intervention** | **Cultural Considerations/Adaptations^a^** |
| --- | --- | --- |
| ***QUALITATIVE STUDIES*** | | |
| Bekelman et al., 2019^b^ | Puente para cuidar (bridge to caring) | **Surface components**  Content: language. Intervention materials were translated into Spanish and “used culturally sensitive and appropriate language.”  Content: “ensured the people being portrayed in materials look like the people being targeted. Video vignettes used Latino/a actors, presence of family, inclusion of spirituality and faith, and settings and activities that resonate with Latino/a community.”  Content: “changed the names of case studies to reflect Latino/a heritage.”  Delivery: used “a bilingual patient navigator as a cultural broker.”  **Deep structure**  Content: “developed scripts and case studies based on core Latino/a values and activities (personlismo - personal relationship, confianze - trust, familia - family, spiritualismo - spirituality, and fatalismo - fatalism).” |
| Chen et al., 2020 | Mind map-based life review program | **Surface components**  Content: language. Intervention was likely conducted in Chinese.  **Deep structure**  Content: albums filled with old photographs of major Chinese historical events at each life stage were designed to evoke participants' memories. |
| Chen et al., 2022^b^ | WeChat-based dyadic life review program | **Surface components**  Content: language. Intervention was likely conducted in Chinese.  Delivery: WeChat is used for the intervention for asynchronous communication and synchronous communication. WeChat allows for “easily accessible life reviews” since WeChat is one of China's most common social communication tools. |
| Fink et al., 2020 | Apoyo con Cariño (Support With Caring) | **Surface components**  Content: education packets are available in English and Spanish to accommodate for the language barrier. Delivery: patient navigators are bicultural and live in local communities to improve cultural sensitivity.  **Deep structure**  Content & Process: patient navigators, “based on core Hispanic values, established a trusting (confianza), personal (personalismo) relationship with patients and family caregivers (familia), focused on goals and values discussion, and worked with them to overcome educational, cultural, and provider/system-level barriers to palliative care.” |
| Houmann et al., 2010^c^ | Dignity therapy | **Surface components**  Content: language. Dignity therapy question protocol was translated into Danish. Intervention was conducted in Danish.  Content: word choice. “Dignity therapy question protocol was modified to reflect Danish cultural sensibilities. Because the meaning of the Danish translation of the word 'alive' in question 2 of DTQP was ambiguous and overly confronting, the tense of the verb was adjusted to mean 'vigorous' instead of 'alive as opposed to dead'. 'Still’ was removed in question 7 to reduce the implication of impending death. 'Permanent' was removed from question 12. The term 'feel', which in Danish may imply a deeply felt need for disclosure, was changed to 'think' (question 7).”  Content: “Careful attention must be paid to how dignity therapy is introduced, ensuring that the language used and the rationale provided not be overly existentially confrontative. In practice, the title would have to be deemphasized when presenting the intervention, and more emphasis be placed on the content of the intervention.”  **Deep structure**  Process: “Danish patients are reticent to talk about things that they feel may be perceived as boastful or simply self-praise. Many patients refused using terms such as accomplishments, importance and pride about themselves or their roles in life. This appears to be a clear cross-cultural difference from the Canadian/Australian setting where dignity therapy was developed. These Danish experiences may be influenced by the 10 commandments referred to as the "Jante Law" -- 'a pattern of group behavior towards individuals within Scandinavian communities, which negatively portrays and criticizes success and achievement as unworthy and inappropriate'...Therapists should ensure that the patient is made comfortable speaking about himself or herself. This must be done in ways that are culturally acceptable and in accord with the patient's outlook.” |
| Kwan et al., 2019^b^ | Short-term life review | **Surface components**  Content: language. Materials were offered all in Chinese. Life review questions were translated from English to Chinese.  Content: 8 life review questions “were modified and culturally adapted from Ando et al. (2010).” |
| Leng et al., 2018; Leng et al., 2019 | Meaning-centered psychotherapy (MCP-Ch) | **Surface components**  Content: language. Patients were interviewed in Mandarin. Intervention will need to be translated into Chinese.  Delivery: adapted intervention is preferred by Chinese patients to be in-person (vs. over the phone).  Content: suggested incorporating “Chinese proverbs and terms”; no "cancer" in materials because of the stigma and taboo around the word.  **Deep structure**  Content: family participation should be considered in meaning-centered psychotherapy intervention, “given the cultural norms around family involvement in medical care.” Patients preferred to have the option of including family members in the MCP-Ch. Intervention should consider the centrality of family in Chinese culture and invite family to participate in group and/or "family" sessions.  Content: “suggested incorporating cancer-related education in the adapted intervention to combat culturally influenced superstitions related to cancer.”  Content: adaptation of intervention should not be one-size fit all given the heterogeneity of Chinese in factors like immigration history, religious belief, and demographics.  Content: address religion in meaning-centered psychotherapy intervention given “the overlap between philosophies of Eastern religions” and Chinese culture.  Content: reframe patients’ immigration experiences in discussions about meaning at the end of life. Intervention can adopt a strengths-based approach building upon patients’ immigration histories, given the historical experience of many Chinese patients with war and losses.  Content: intervention can address the concept of a good death and its implications on familial and financial responsibilities and conflicts, given its emphasis in Chinese culture.  Content: need to address symptom control issues given “Chinese patients’ tendency to underreport pain.”  Content: meaning-centered psychotherapy session that regards humor as a source of meaning could be modified since culturally, humor is not practical in the cancer setting. |
| Li et al., 2014 | Dignity therapy | **Surface components**  Content: language. Intervention was conducted in Mandarin.  **Deep structure**  Content & Process: “in Taiwan, most dying patients and their families do not like to directly discuss death or dying (Mak 2007). Therefore, the word death or dying was avoided during the interview unless patients talked about it themselves.” |
| Lin et al., 2020 | Advanced care planning intervention | **Surface components**  Content: language. Intervention was conducted in Chinese. Participants were required to be able to speak Taiwanese or Mandarin.  **Deep structure**  Process: considered the importance of family involvement in patients' advanced care planning preparation and consultation to support their decision-making in line with existing expectations that families take a role. |
| Lin et al., 2022 | Dignity therapy | **Surface components**  Content: language. Intervention was likely conducted in Chinese for patients in mainland China.  Note: study did not provide suggestions on how to adapt dignity therapy but collected DT therapists' experiences on how Chinese culture influences their delivery of intervention to patients. |
| Liossi et al., 2001^b^ | Hypnosis | **Surface components**  Content: language. Intervention was conducted in Greek. Participants were required to know Greek, and they were excluded “if Greek was not their first language.” |
| Nunziante et al., 2021 | Dignity therapy | **Surface components**  Content: language. Intervention was conducted in Italian. “The Italian version of the Dignity Therapy Question Protocol was employed.” |
| Ólafsdóttir et al., 2018 | Advanced care planning intervention | **Surface components**  Content: language. Intervention was conducted in Icelandic. Participants were required to be able to speak and understand Icelandic. “The booklet "Thinking ahead一what's important to me" was translated and adapted to facilitate the discussion among the patient, his/her family, and the palliative care nurse.” |
| Patel et al., 2019 | Lay health workers educate engage and encourage patients to share (LEAPS) cancer care | **Surface components**  Content: language. Intervention included a translated advance directive (in patients' preferred languages).  Delivery: intervention utilized multilingual lay health workers from the community to provide culturally-sensitive education and disease management. In Atlantic City, lay health workers are fluent in English and Hindi, Spanish, or Mandarin. In Chicago, lay health workers speak English and Hindi or Spanish. |
| Pon, 2010 | My Wonderful Life Board Game | **Surface components**  Content: language. Intervention was conducted in Chinese. Board game materials are in Chinese.  **Deep structure**  Process: “the use of therapeutic play could facilitate interaction and expression for Chinese people who often have difficulties sharing intimate and intense emotions and topics.”  Overall selection of intervention: therapeutic game play. “The use of conventional verbal intervention models may be overwhelming for Chinese people; instead, they may respond to alternative means of engaging them that facilitate interaction and expression.” |
| Schulman-Green et al., 2022 | Managing Cancer Care | **Surface components**  Content: language. Intervention was conducted in English or Hebrew. Participants were required to be English or Hebrew speaking. “The Managing Cancer Care tool was professionally translated from English to Hebrew.” |
| Takenouchi et al., 2022 | Lifeline Interview Method | **Surface components**  Content: language. Intervention was conducted in Japanese. Participants were required to have the ability to have an advanced care planning conversation in Japanese.  **Deep structure**  Process: “the nature of the Lifeline Interview Method requires active self-involvement and description of the reasons for emotions...Self-focused life story event recounting through the Lifeline Interview Method is likely to encourage people from Asian backgrounds who value family-centered decision-making to talk about what matters to them” (to help bridge advanced care planning discussions). |
| Torres-Blasco et al., 2022 | Caregivers-Patients Support to Latinx coping advanced-cancer (CASA) | **Surface components**  Content: 7 dimensions were addressed for cultural and linguistic adaptation - language, context, persons, metaphors, concepts, goals, and methods. “For example, language: translate the couple communication skills training into Spanish and eliminate spousal terms, metaphors: investigators will include culturally consonant stories by adapting the communication skills training and meaning-centered components, and concepts: integration of culturally consonant meaning-centered psychotherapy concepts and important end-of-life care topics.”  **Deep structure**  Content: 7 dimensions addressed for cultural and linguistic adaptation. For example, context: Integration of Latino family (caregivers-patients) values, traditions, and uniqueness in communication and meaning. Content: “integrate Latinx families and cultural values (i.e., spirituality, familism and fatalism) to the Caregivers-Patients Support to Latinx coping advanced-cancer (CASA) manual. Research team will interview patients and caregivers to gather information from them about integrating values into the intervention.”  Overall selection of intervention: “a culturally centered intervention for Latinx patients should include a family-centered (partners and other family members) approach to determine the content and goals of care preferred in Latinx families coping with cancer.”  Content: researchers integrated family related content into the intervention. “Family is a core value in the Latinx community and may facilitate the caring process for this group's advanced cancer patients.” |
| van den Hurk et al., 2015^b^ | Mindfulness-based stress reduction | **Surface components**  Content: language. Intervention was conducted in Dutch. Participants were required to be able to understand Dutch. Exclusion criteria included not being able to understand or use the Dutch language. |
| Wang et al., 2019^b^ | Family participatory dignity therapy | **Surface components**  Content: language. Intervention was likely conducted in Chinese.  Content: intervention was designed by reviewing the dignity therapy questions and restating each question in a fashion that would be applicable to the Chinese population.  Delivery: “the final vivid e-product can be shared with anyone they wish by the patient-family dyad via social networking software (WeChat & QQ are popular social software among Chinese). Also, photos were collected from participants by means of WeChat, QQ or other social networking software.”  **Deep structure**  Content: included family caregiver in the intervention. “The family, rather than the individual, is seen as the basic unit of life in China. In China, cancer is viewed not only as a personal traumatic experience, but also as a family event. Research indicated that patients’ sense of dignity can be enhanced through strengthening family integrity, the perception of being loved and mutual compassion, which involves the cultivation of filial support and understanding, gained through open communication with their family members.”  Process: “the question prompt was revised to better suit Chinese expression habits, which should be mild, implicit and inoffensive.”  Content & Process: “Chinese culture regards cancer, and death in general, as taboo subjects and discourages their discussion. It is ominous to talk about death when the person is still alive. Hence, words such as death, dying or end of life should be avoided throughout the course of the intervention to protect participants from feeling depressed or offended. However, such a topic should be raised as part of family participatory dignity therapy if the patient wishes to discuss it.” |
| Xiao et al., 2012 | Life review programme | **Surface components**  Content: language. Intervention was likely conducted in Chinese.  **Deep structure**  Content: integrated Chinese culture into Haight and Bahr's structured life review to produce a specific life review program for Chinese patients.  Content: development of intervention was based on the Confucian thoughts on human development. Intervention included “common Chinese life themes from Confucian thoughts to increase the cultural relevance of the program. Confucian thoughts on human development are mainly Confucius' own account of his development: "At 15, I set my heart upon learning. At 30, I was firmly established. At 40, I had to more doubts. At 50, I knew the Decree of Heaven. At 60, I was attuned to Heaven. At 70, I could follow my heart's desire without violating the regulations." This is an appropriate model of behavior for others to emulate.”  Content: included common experiences specific to Chinese people to increase the cultural relevance of the program. “The Chinese Cultural Revolution from 1966 to 1975 had a great impact on many Chinese people, who are now older than 30 years. They commonly experienced inadequate food, dropped out of school, worked as children, or left home to work in the countryside.”  Overall selection of intervention: significance of life review document. “Regarding the life review booklet, in China, a document has a specific cultural value. Chinese people believe that a person's value in life is reflected in 3 aspects ("3 Eternities in Life"), namely, "Li Gong" (meritorious service), "Li De" (high moral values), and "Li Yan" (great writings). When the person meets these requirements, his/her spirit achieves immortality. A life review booklet is a type of Li Yan and thus may help patients affirm value in their life.” |
| Yang et al., 2021 | Educate, Nurture, Advise, Before Life Ends (ENABLE) | **Deep structure**  Content: interventionists “assess preferences for decision making and tailor content to cater for individual family dynamics.” “In a family-centric cultural context, patients may not be fully aware of their cancer diagnosis and prognosis...Families may feel it is their responsibility to receive bad news and decide how much should be disclosed to the patient, so as to protect the sick patient from unnecessary worry and to preserve hope; there is also a cultural obligation for the family member to bear the burden of making decisions on behalf of the patient. In the context of ENABLE which seeks to help patients and family caregivers cope with problems arising from advanced cancer and/or cancer treatment, participants should at least be aware of their cancer diagnosis. However, in order to ensure that healthcare interventions are aligned with family-centric cultural values, the content of ENABLE-SG should be appropriate for patients who do not know the full extent of their cancer and where decisions are delegated to a family member. Sessions 1 to 4 of the original ENABLE model focus on coping skills that are applicable at all stages of cancer, and will only require minor modifications so that they can be conducted for patients who are aware of their cancer diagnosis but not their prognosis. For example, in the section on healthy eating and nutrition, content on how diminished appetite can be due to advancing illness or inability to control the cancer could be removed. Sessions 5 and 6 on life review and creating a legacy may be challenging to address if the patient is not aware of their advanced stage of cancer. However, the nurse coach would have developed sufficient rapport with the patient by that time to judge whether it would be appropriate to proceed with these sessions sensitively, or defer them to a subsequent monthly follow-up call when the opportunity arises.” |
| ***RANDOMIZED CONTROL STUDIES*** | | |
| Anderson et al., 2004^c^ | Pain education | **Surface components**  Content: language. Intervention was conducted in English and Spanish.  Delivery: intervention utilized bilingual research nurses.  **Deep structure**  Content: “content of each video and booklet targeted specific sex and ethnic groups.” “Videos and booklets were specific for underserved African American men, African American women, Hispanic men, and Hispanic women.”  Content: addressed pain management barriers, such as ethnic-specific “stoicism, concerns about possible addiction to opioid medications” and “reluctance to complain about pain” and question medication efficacy to healthcare providers. |
| Ando et al., 2010 | Short-term life review | **Surface components**  Content: language. Intervention was likely conducted in Japanese. |
| Bakitas et al., 2009^c^ | Educate, Nurture, Advise, Before Life Ends (ENABLE II) | **Surface components**  Delivery: intervention was primarily conducted by telephone in order to be accessible to a rural population that was anticipated to become more dependent as illness progressed. |
| Bouchard et al., 2019^c^ | Cognitive behavioral stress management | **Surface components**  Content: adapted cognitive behavioral stress management for men with advanced prostate cancer by “providing skills relevant for men with advanced prostate cancer.”  Delivery: intervention was administered via tablets to “reduce barriers to recruiting and retaining minority men (i.e., Black men).”  Delivery: “to accommodate minority participants and patients with advanced disease, the researchers provided transportation for in-person assessments, flexible schedules for group sessions, and training for using the study’s technology.”  Process: “to accommodate minority participants and patients with advanced disease, the researchers provided extensive education about the study’s purpose and goals.” |
| Caruso et al., 2020^c^ | Managing Cancer and Living Meaningfully (CALM) | **Surface components**  Content: language. Intervention was likely conducted in Italian. |
| Chen et al., 2020; Chen et al., 2022 | Mind map-based life review program | **Surface components**  Content: language. Intervention was likely conducted in Chinese.  **Deep structure**  Content: “albums filled with old photographs of major Chinese historical events at each life stage were designed to evoke participants' memories.” |
| Cheung et al., 2020^c^ | Self-administered acupressure | **Surface components**  Content: language. Intervention was conducted in Chinese. Participants were required to have the “ability to communicate in Cantonese or Putonghua.”  Overall selection of intervention: “acupressure is a non-invasive technique based on the meridian theory of traditional Chinese medicine.” |
| Cheung et al., 2021 | Aerobic exercise & Tai-chi | **Surface components**  Content: language. Intervention was conducted in Chinese or English. Participants were required to be “able to communicate in Cantonese, Mandarin, or English.”  Overall selection of intervention: “Tai-chi is a form of qigong, which is a mind-body intervention rooted in a Traditional Chinese medicine concept and is a preferable complementary and alternative medicine for insomnia in the Chinese population.” |
| Dionne-Odom et al., 2021 | ENABLE (Educate, Nurture, Advise, Before Life Ends) Cornerstone | **Surface components**  Delivery: mixed in-person and telephonic delivery to accommodate African Americans and rural-dwelling individuals. |
| Du et al., 2022^c^ | Heart to Heart Card Game | **Surface components**  Content: language. Intervention was conducted in Chinese. Participants were required to be Chinese speaking.  **Deep structure**  Process: “Due to the Chinese 'taboo death' culture, communicating with individuals with advanced cancer and knowing their preferences poses more barriers and concerns for Chinese medical workers. Thus, the Chinese American Coalition for Compassionate Care developed the Heart to Heart Card Game, based on Go Wish cards, to help Chinese healthcare providers to initiate end-of-life conversations.”  Content: “The patients' cultural backgrounds (e.g., religion, economics, cultural values) and preferences were ascertained by asking the participants to choose the important cards and having a semi-structured end-of-life conversation, and then the patient's palliative care and family care were adjusted or maintained according to the patient's sharing results.” |
| Fischer et al., 2018^c^ | Apoyo con Cariño (Support With Caring) | **Surface components**  Content: education packets are available in English and Spanish to accommodate for the language barrier. Delivery: patient navigators are bicultural and live in local communities to improve cultural sensitivity.  **Deep structure**  Content & Process: patient navigators, “based on core Hispanic values, established a trusting (confianza), personal (personalismo) relationship with patients and family caregivers (familia), focused on goals and values discussion, and worked with them to overcome educational, cultural, and provider/system-level barriers to palliative care.” |
| Fraguell-Hernando et al., 2020 | Individual Meaning-Centered Psychotherapy-Palliative Care | **Surface components**  Content: language. Intervention was conducted in Spanish. |
| Gil et al., 2018^c^ | Meaning-Centered Psychotherapy-compassionate palliative care | **Surface components**  Content: language. Intervention was conducted in Spanish. All participants were Spanish-speaking. |
| Han et al., 2021 | Naikan and Morita Therapies | **Surface components**  Content: language. Intervention was conducted in Chinese. Participants were required to be able to understand and read Chinese.  **Deep structure**  Overall selection of intervention: Morita therapy is a systematic psychological therapy based on Eastern philosophy. |
| Huang et al., 2019; Huang et al., 2021 | Magnanimous therapy | **Surface components**  Content: language. Intervention was likely conducted in Chinese.  **Deep structure**  Content: “Magnanimous Therapy was developed from inspirations from religions, such as Buddhism and Dao. They make people raise their insights into, and understanding of, life through stories, poems, and Buddhist Bang and Bawl, which can help them express and process difficult thoughts and emotions through either a direct or indirect approach. References for MT are from Buddhism and Zen (Chan) and Taoism (Daoism). Information inputted include little stories, cases, selected parts of films or TV programmes, poems, a Bang and a Bawl to Waken One from Error, etc. (Huang X (2018) Introduction of magnanimous psychotherapy. Universal Journal of Psychology 6(3): 80–86.)” |
| Julião et al., 2013; Julião et al., 2014; Julião et al., 2017^c^ | Dignity therapy | **Surface components**  Content: language. Intervention was conducted in Portuguese. Participants were required to have the ability to read and speak Portuguese. |
| Kim et al., 2018 | ILOVEBREAST mobile game | **Surface components**  Content: language. Intervention was conducted in Korean. Screenshots of the ILOVEBREAST mobile game provided in the study show game content in Korean language. |
| Li et al., 2019^c^ | Wellness education | **Surface components**  Content: language. Intervention was likely conducted in Chinese. |
| Liao et al., 2013 | Chinese Medicine five-element music | **Surface components**  Content: “Chinese Medicine five-element music is a folk music under the guidance of Chinese medicine.” “The history of Chinese medicine five-element music dates back thousands of years and it is part of traditional Chinese folk music.”  **Deep structure**  Overall selection of intervention: “Chinese medicine five-element music is designed and produced based on the relationship between the five elements (wood, fire, earth, metal, and water), and the five tunes. It aims to balance the yin and yang, regulate qi and blood, and maintain the human body in a state of dynamic homeostasis, and keeping the individual in good health.” |
| Maungtoug et al., 2021^c^ | Ritualized chanting in palliative care | **Surface components**  Content: language. Intervention was likely conducted in Thai.  **Deep structure**  Overall selection of intervention: “In Thailand, Buddhism is a way of life of the majority of Thais, and Buddhist principles are solidly infused into Thai culture and practiced in everyday living.” Study authors aim to investigate Buddhist practice of chanting as an intervention to improve comfort. |
| Molassiotis et al., 2021 | Patient- and family-centered psychosocial-based nutrition intervention PIcNIC & PiCNIC2 | **Surface components**  Content: language. Culturally-adapted booklet provided to patients and caregivers was in English and Chinese.  **Deep structure**  Content: “food and eating are culturally bound concepts, and as such the content of the intervention also needs to be reflective of cultural realities and preferences.” |
| Onyechi et al., 2016^c^ | Rational emotive hospice care therapy | **Surface components**  Content: language. Intervention was conducted in Igbo. |
| Park et al., 2020 | Lifestyle intervention | **Surface components**  Content: language. Intervention was likely conducted in Korean. |
| Patel et al., 2020^c^ | Lay health workers educate engage and encourage patients to share (LEAPS) cancer care | **Surface components**  Content: language. Intervention included a translated advance directive (in patients' preferred languages).  Delivery: “intervention utilized multilingual lay health workers from the community to provide culturally-sensitive education and disease management. In Atlantic City, lay health workers are fluent in English and Hindi, Spanish, or Mandarin. In Chicago, lay health workers speak English and Hindi or Spanish.” |
| Quílez-Bielsa et al., 2022^c^ | Meaning-centered psychotherapy-essential care | **Surface components**  Content: language. Intervention was conducted in Spanish. All participants were Spanish speaking. |
| Teo et al., 2019 | Cognitive behavioral therapy symptom management combined with acceptance and commitment therapy mindfulness and values-guided principles | **Deep structure**  Overall selection of intervention: “traditional cognitive behavioral symptom management protocols have largely been developed in Western countries. Mindfulness principles in acceptance commitment therapy have originated from Eastern cultures. Combination of CBT and ACT strategies may be particularly applicable in diverse cultural settings.” |
| Teo et al., 2020 | Cognitive behavioral therapy-based intervention | **Surface components**  Content: language. Intervention was conducted in English and Chinese. Participants were required to be able to speak and read English or Mandarin.  **Deep structure**  Content: intervention material was adapted for local use, such as providing examples and discussions surrounding social roles and impact on family. |
| Xiao et al., 2013 | Life review programme | **Surface components**  Content: language. Intervention was likely conducted in Chinese.  **Deep structure**  Content: integrated Chinese culture into Haight and Bahr's structured life review to produce a specific life review program for Chinese patients.  Content: development of intervention was based on Confucian thoughts on human development. Intervention included “common Chinese life themes from Confucian thoughts to increase the cultural relevance of the program. Confucian thoughts on human development are mainly Confucius' own account of his development: At 15, I set my heart upon learning. At 30, I was firmly established. At 40, I had to more doubts. At 50, I knew the Decree of Heaven. At 60, I was attuned to Heaven. At 70, I could follow my heart's desire without violating the regulations.”  Content: included common experiences specific to Chinese people to increase the cultural relevance of the program. “The Chinese Cultural Revolution from 1966 to 1975 had a great impact on many Chinese people, who are now older than 30 years. They commonly experienced inadequate food, dropped out of school, worked as children, or left home to work in the countryside.”  Overall selection of intervention: significance of life review document. “Regarding the life review booklet, in China, a document has a specific cultural value. Chinese people believe that a person's value in life is reflected in 3 aspects ("3 Eternities in Life"), namely, "Li Gong" (meritorious service), "Li De" (high moral values), and "Li Yan" (great writings). When the person meets these requirements, his/her spirit achieves immortality. A life review booklet is a type of Li Yan and thus may help patients affirm value in their life.” |
| Xiao et al., 2022 | Family-oriented dignity therapy | **Surface components**  Content: language. Intervention was conducted in Chinese. Participants were required to be able to read and communicate in Chinese (Mandarin).  **Deep structure**  Content: intervention development was based on the author's previous study on Chinese perception of dignity. “The modeling process included a qualitative study of dignity perception and its associated factors in patients with cancer undergoing chemotherapy in mainland China; similarities and discrepancies were identified with respect to cultural differences and the qualitative study results were compared with the assumptions of the dignity model (Xiao et al., 2020). Family support and communication between patients and families were highlighted; specifically, a family-oriented approach involving family caregivers to improve communication was recommended to enhance patients’ sense of dignity.” |
| Yanez et al., 2015^c^ | Cognitive behavioral stress management | **Surface components**  Content: cognitive behavioral stress management intervention was adapted “to provide didactics and examples relevant to men with advanced prostate cancer (eg, impotence, incontinence, and intimacy concerns) and provide additional skills that are more appropriate for men with advanced prostate cancer (eg, acceptance, existential concerns, and life narratives).” |
| Ye et al., 2017^c^ | Be Resilient to Breast Cancer program | **Surface components**  Content: language. Intervention was conducted in Chinese. Participants were required to be fluent in oral Mandarin or Cantonese.  **Deep structure**  Content: Be Resilient to Breast Cancer program was adapted from supportive-expressive group therapy (SEGT) and culturally tailored for Chinese females with metastatic breast cancer.  Content & Process: “To better adapt to Chinese culture, the investigators added education hosted by professional staff (e.g., clinical psychologists, dietician, Chinese medicine practitioner and so on) in an effort to foster self-efficacy to combat symptoms (such as pain, fatigue, intrusive thoughts and so on) through knowledge and techniques (such as breath control, meditation and so on), and to help patients gain a sense of control in their life.”  Delivery & Process: “Trained mentors, who were breast cancer survivors themselves, were added to the group discussion to create non-hierarchical, reciprocal relationships through the sharing of experiences with those facing similar challenges.” |
| Zheng et al., 2022^c^ | Very important person (VIP) for future care | **Surface components**  Content: language. Intervention was likely conducted in Chinese.  **Deep structure**  Overall selection of intervention: utilized a Chinese advanced care planning intervention developed by Chinese researchers. This study utilized a “localized advanced care planning intervention program - very important person (VIP) for future care - to assist patients in expressing their end-of-life wishes, and the feasibility study of this intervention model has been completed in the eastern Chinese city of Zhuhai. At present, only the application of this model in eastern China has been reported, but due to the differences in culture, economy, and belief, the applicability “VIP Future Nursing” intervention model in advanced cancer patients in the West still needs to be explored.” |
| ***QUASI-EXPERIMENTAL STUDIES*** | | |
| Catania et al., 2021 | INtervention FOcused on quality of life assessment (INFO-QoL) | **Surface components**  Content: language. Intervention was likely conducted in Italian. |
| Chimluang et al., 2017^c^ | Intervention based on basic Buddhist principles | **Surface components**  Content: language. Intervention was conducted in Thai. Participants were required to be “literate in the Thai language.”  **Deep structure**  Content: Intervention is “based on basic Buddhist principles: 1) precept training (i.e. training of morality of conduct for good moral behavior); 2) concentration training (i.e. mental study for a peaceful mind and great quality of the mind); and 3) wisdom training (i.e. intellectual study to live happily with recognition of the world and real life).” This is from “one of the basic crucial principles of dharma, the law of nature, which is called 'three-fold training' in the Buddhist religion.” |
| Ichihara et al., 2019 | Spiritual care using Spiritual Pain Assessment Sheet (SpiPas) | **Surface components**  Content: language. Intervention was conducted in Japanese. Participants were required to be able to communicate in Japanese. |
| Landa-Ramírez et al., 2020^c^ | Cognitive behavioral therapy | **Surface components**  Content: language. Intervention was conducted in Spanish.  Content: language and idiosyncrasies. Treatment was provided using the cultural meaning of Mexican-Spanish words. Cultural likes were taken to create better relationships and understanding of concepts.  Delivery: home-based intervention. Intervention considered the Mexican cultural element of poverty. Home-based therapy reduces the burden of travel on patients and families who may be unable to visit the hospital due to lack of financial resources.  **Deep structure**  Content: Treatment protocol was adapted to socioeconomic and psychosocial contextual issues, such as the Mexican cultural element of poverty.  Delivery: Flexibility for integrating extended family into cognitive behavioral therapy. Many sessions in the study were provided on weekends before the meal time when all the relatives were together. Intervention incorporated the Mexican cultural element of extended family structure. |
| Lee et al, 2017 | Mindfulness-based stress reduction program | **Surface components**  Content: language. Intervention was conducted in Korean. Participants were required to be able to read and write in Korean. |
| Li et al., 2020^c^ | Dignity therapy | **Surface components**  Content: language. Intervention was conducted in Chinese. Participants were required to be able to communicate verbally in Mandarin or Taiwanese Hokkien. |
| Patel et al., 2021^c^ | Lay health workers educate engage and encourage patients to share (LEAPS) cancer care | **Surface components**  Content: language. Intervention included a translated advance directive (in patients' preferred languages).  Delivery: intervention utilized multilingual lay health workers from the community to provide culturally-sensitive education and disease management. In Atlantic City, lay health workers are fluent in English and Hindi, Spanish, or Mandarin. In Chicago, lay health workers speak English and Hindi or Spanish. |
| Zhang et al., 2019 | WeChat-based life review programme | **Surface components**  Content: language. Intervention was likely conducted in Chinese.  Delivery: intervention is WeChat-based. “WeChat is a multi-functional social networking application covering 90% of mobile phones in China.” |
| ***SINGLE ARM TRIALS*** | | |
| Ando et al., 2008 | Short-term life review | **Surface components**  Content: language. Intervention was likely conducted in Japanese. |
| Ando et al., 2016 | Mindfulness art therapy | **Surface components**  Content: language. Intervention was likely conducted in Japanese. |
| Delrieu et al., 2020 | Physical activity program | **Surface components**  Content: language. Intervention was likely conducted in French. |
| Houmann et al., 2014**^c^** | Dignity therapy | **Surface components**  Content: language. Dignity therapy question protocol was translated into Danish. Intervention was conducted in Danish.  Content: word choice. “Dignity therapy question protocol was modified to reflect Danish cultural sensibilities. Because the meaning of the Danish translation of the word 'alive' in question 2 of DTQP was ambiguous and overly confronting, the tense of the verb was adjusted to mean 'vigorous' instead of 'alive as opposed to dead'. 'Still’ was removed in question 7 to reduce the implication of impending death. 'Permanent' was removed from question 12. The term 'feel', which in Danish may imply a deeply felt need for disclosure, was changed to 'think' (question 7).”  Content: “Careful attention must be paid to how dignity therapy is introduced, ensuring that the language used and the rationale provided not be overly existentially confrontative. In practice, the title would have to be deemphasized when presenting the intervention, and more emphasis be placed on the content of the intervention.”  **Deep structure**  Process: “Danish patients are reticent to talk about things that they feel may be perceived as boastful or simply self-praise. Many patients refused using terms such as accomplishments, importance and pride about themselves or their roles in life. This appears to be a clear cross-cultural difference from the Canadian/Australian setting where dignity therapy was developed. These Danish experiences may be influenced by the 10 commandments referred to as the "Jante Law" -- 'a pattern of group behavior towards individuals within Scandinavian communities, which negatively portrays and criticizes success and achievement as unworthy and inappropriate'. Therapists should ensure that the patient is made comfortable speaking about himself or herself. This must be done in ways that are culturally acceptable and in accord with the patient's outlook.” |
| Kang et al., 2015 | Meaning of My Life | **Surface components**  Content: language. Intervention was likely conducted in Korean. |
| Li et al., 2015 | Caring for couples coping with cancer '(4Cs)' program | **Surface components**  Content: language. Intervention was conducted in Chinese. Participants were required to communicate with the interviewers in Mandarin. |
| Nakayama et al., 2009**^c^** | Music therapy | **Surface components**  Content: traditional and popular Japanese songs were used.  **Deep structure**  Process: music therapy was provided through a group format. “One-on-one music therapy sessions in a private room in hospices are unpopular in Japan due to nervousness and personal reservation.” |
| Niki et al., 2019 | Virtual reality | **Surface components**  Content: language. Intervention was likely conducted in Japanese. |
| Ramos et al., 2018^c^ | Life program | **Deep structure**  Content: Life Program includes “discussion of psychological stressors and existential concerns unique to Veteran populations living with advanced disease, such as reflections of combat post-traumatic stress symptoms being triggered with a life-threatening diagnosis, military culture of seeking help, and societal and self-stigma of mental illness.” |
| Sakaguchi et al., 2015**^c^** | Collage Activity Based on Life Review | **Surface components**  Content: language. Intervention was conducted in Japanese. |
| Warth et al., 2018 | Song of Life | **Surface components**  Content: language. Intervention was conducted in German and English. Participants were required to be German or English speaking.  Content: selection of music pieces. “Patients were asked to choose a song which they perceived as a resource and not as a burden in that moment. In case the patient was unable to identify a personal Song of Life, the therapist suggested a popular song depending on the patient’s age. Majority of songs/pieces were German (classical music, sacred music, pop music, German folk music, lullaby). This is a surface structure tailored by the researchers going in, not necessarily deep structure designed by them**.”** |
| ***FEASIBILITY/ACCEPTABILITY TRIALS*** | | |
| Hanson et al., 2013 | Circles of Care | **Surface components**  Delivery: utilized lay health advisors from the African American community, including church leaders and health ministries. |
| Molassiotis et al., 2018 | Patient- and family-centered psychosocial-based nutrition intervention PIcNIC & PiCNIC2 | **Surface components**  Content: language. The Chinese language was used at the Hong Kong site.  **Deep structure**  Delivery: In contrast to the group in Australia, the intervention in Hong Kong was delivered to the patient and family together as a family-centered intervention. |
| ***PROTOCOLS*** | | |
| Costas-Muñiz et al., ongoing study | Meaning-centered psychotherapy for Latinos | **Surface components**  Content: language. Intervention was conducted in Spanish. Participants are required to be fluent in oral Spanish.  Content: “researchers will adapt and transcreate the manual (the source text is rewritten to convey the concepts and achieve the aims of the source text, while accounting for both language and cultural considerations).”  **Deep structure**  Content: “In phase 5 of the study, researchers will collect data on acceptability of MCP-L content. They will collect data for attitudinal familism, acculturation, fatalism, religiosity, and literacy, which will inform the design of a future large-scale randomized clinical trial of the culturally adapted intervention.” |
| Matthys et al., 2021 | Face-to-face FOCUS+ & Web-based iFOCUS | **Surface components**  Content: language. Intervention was conducted in different languages specifically for the countries where the experiment took place (Belgium, Denmark, Ireland, Italy, the Netherlands, and the United Kingdom). |
| Miyamoto et al., 2022 | Managing Cancer and Living Meaningfully (CALM) | **Surface components**  Content: language. Intervention was conducted in Japanese. Participants were required to be fluent in Japanese.  **Deep structure**  Overall selection of intervention: “Japanese cancer patients prefer psychotherapy to drug therapy as treatment for depression. Many Japanese patients hesitate in taking psychiatric drugs because of prejudice or stigma.” |
| Scheffold et al., 2015 | Managing Cancer and Living Meaningfully (CALM) | **Surface components**  Content: language. Intervention was conducted in German. Participants were required to speak German fluently. “The CALM treatment manual was translated and adapted into the German language.” |
| Torres-Blasco et al., 2022 | Caregivers-Patients Support to Latinx coping advanced-cancer (CASA) | **Surface components**  Content: 7 dimensions were addressed for cultural and linguistic adaptation - language, context, persons, metaphors, concepts, goals, and methods. “For example, language: translate the couple communication skills training into Spanish and eliminate spousal terms, metaphors: investigators will include culturally consonant stories by adapting the communication skills training and meaning-centered components, and concepts: integration of culturally consonant meaning-centered psychotherapy concepts and important end-of-life care topics.”  **Deep structure**  Content: 7 dimensions addressed for cultural and linguistic adaptation. For example, context: Integration of Latino family (caregivers-patients) values, traditions, and uniqueness in communication and meaning.  Content: “integrate Latinx families and cultural values (i.e., spirituality, familism and fatalism) to the Caregivers-Patients Support to Latinx coping advanced-cancer (CASA) manual. Research team will interview patients and caregivers to gather information from them about integrating values into the intervention.”  Overall selection of intervention: “a culturally centered intervention for Latinx patients should include a family-centered (partners and other family members) approach to determine the content and goals of care preferred in Latinx families coping with cancer.”  Content: researchers integrated family related content into the intervention. “Family is a core value in the Latinx community and may facilitate the caring process for this group's advanced cancer patients.” |
| van der Wel et al., 2022 | Family, outlook, coping, uncertainty, symptom management (FOCUS+) | **Surface components**  Content: country-specific languages were used in the intervention (Belgium, Denmark, Ireland, Italy, the Netherlands, and the UK).  Content: additional information materials and resources are country-specific and adapted to national healthcare systems and regulations.  Content: “each country selected national equivalents of the original USA additional resources because each country was expected to have many equivalents of the materials, and the original American materials often did not comply with the diversity of healthcare services and support in Europe.”  Content: “psychological interventions were considered not to be a formal responsibility of nurses in the European countries involved, which may be different from the role of (oncology) nurses in the USA. The goal of the FOCUS+ program should be framed as improving the dyad’s self-management and self-efficacy by providing information and psychoeducation to emphasize the supportive and educational nature of the nursing intervention.” |
| Zhang et al., 2018 | WeChat-based life review programme | **Surface components**  Content: language. Intervention was likely conducted in Chinese.  Delivery: intervention is WeChat-based. “WeChat is a multi-functional social networking application covering 90% of mobile phones in China.” |

***Note.*** ^a^Coding was completed according to authors’ written description of cultural consideration efforts. ^b^Included in meta-analysis. ^c^Excluded from meta-analysis due to insufficient data.

### Appendix F: Additional Forest Plots of Effect Sizes

#### **Table 3a. RCTs and Quasi-Experimental Studies** **Physical Symptoms (n=5, k=10) – RCT & Quasi-Experimental**


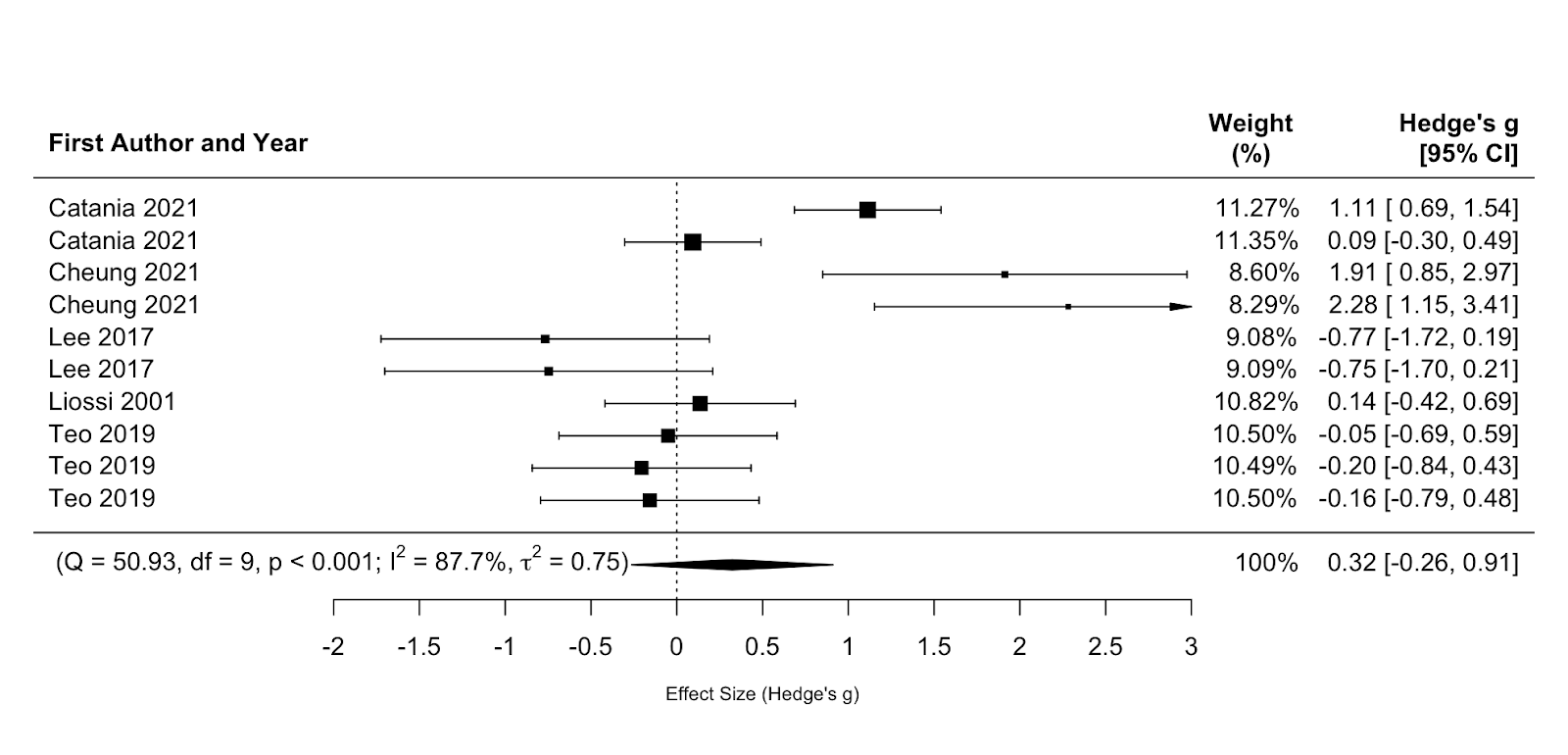


##### **Depression (n=15, k=15) – RCT & Quasi-Experimental**


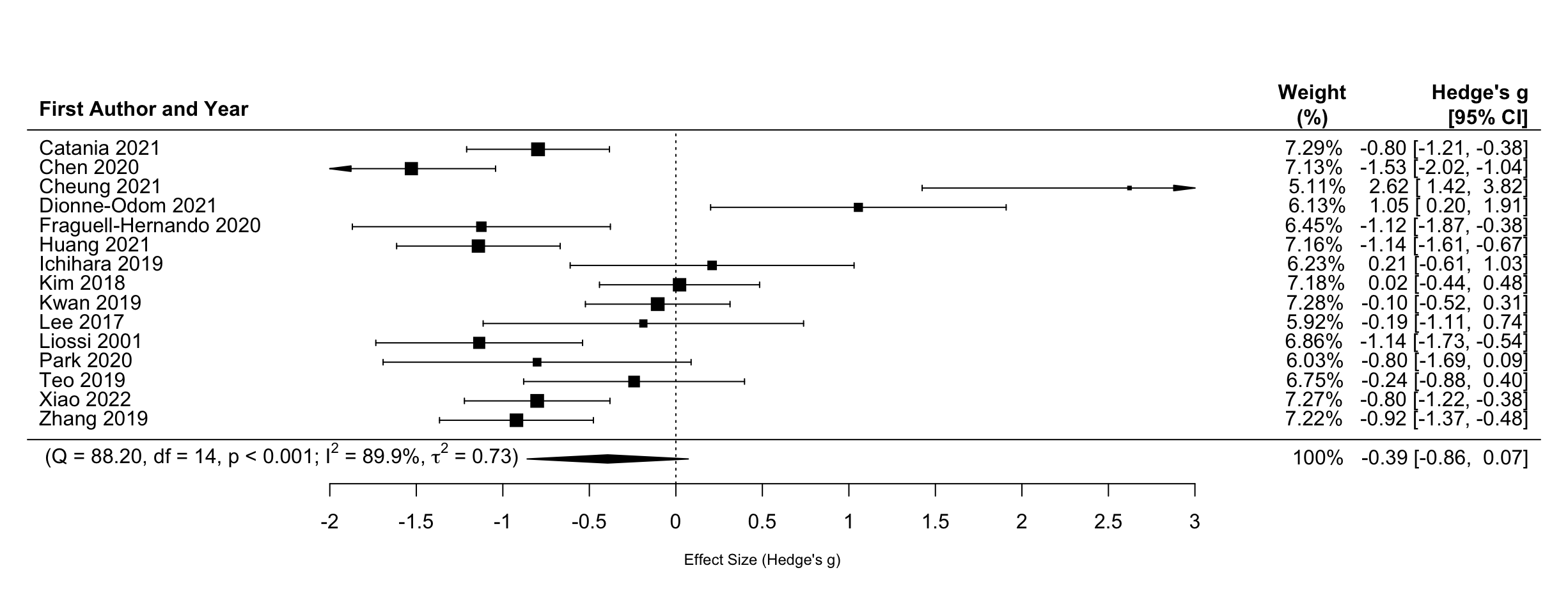


#### **Table 3b. Single Arm Trials**

##### **Quality of Life (n=4, k=5) – Single Arm**
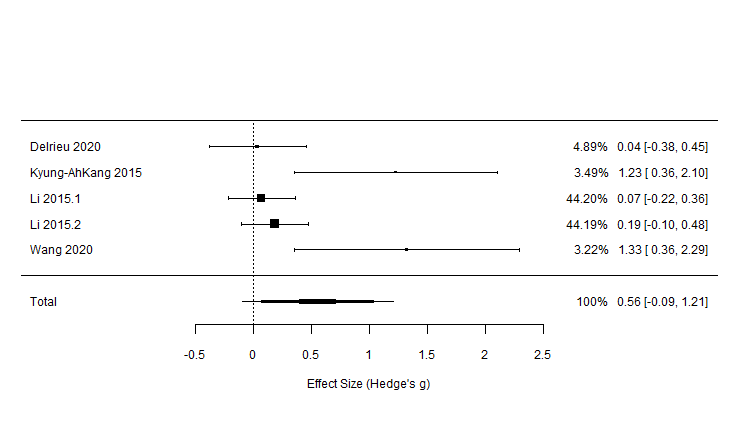


**Study Weight *g* [95% CI]]**

##### **Physical Symptoms (n=5, k=10) – Single Arm**


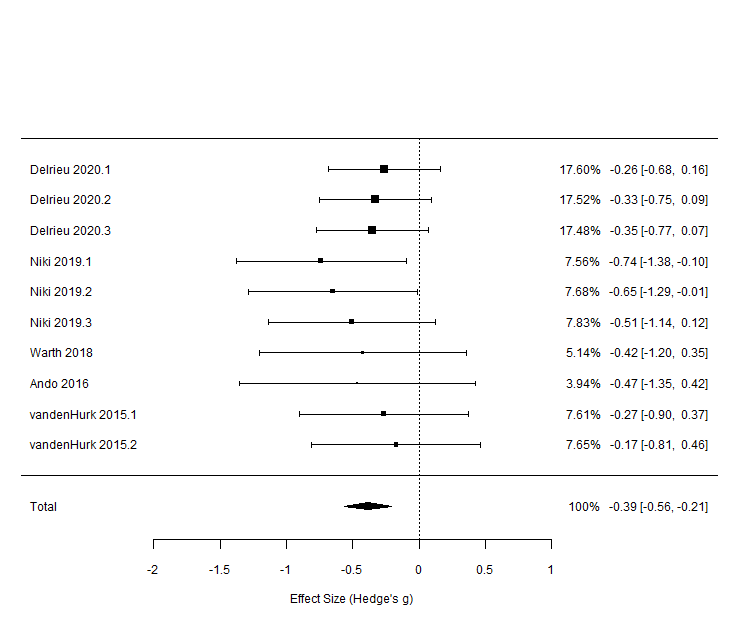


**Study Weight *g* [95% CI]]**

##### **Anxiety (n=6, k=6) – Single Arm**


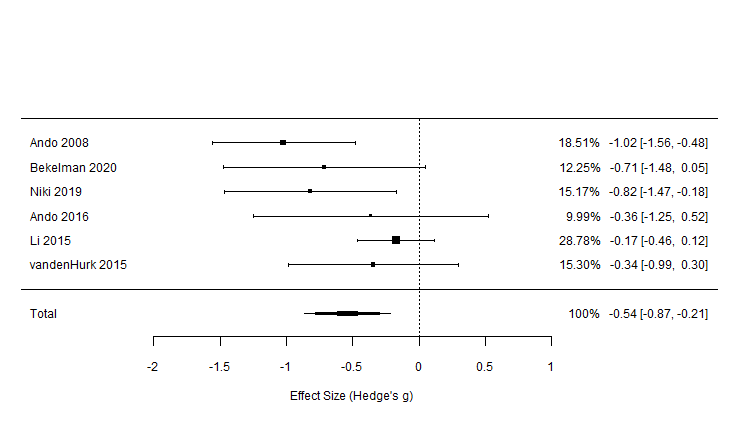


**Study Weight *g* [95% CI]]**

##### **Depression (n=6, k=6) – Single Arm**


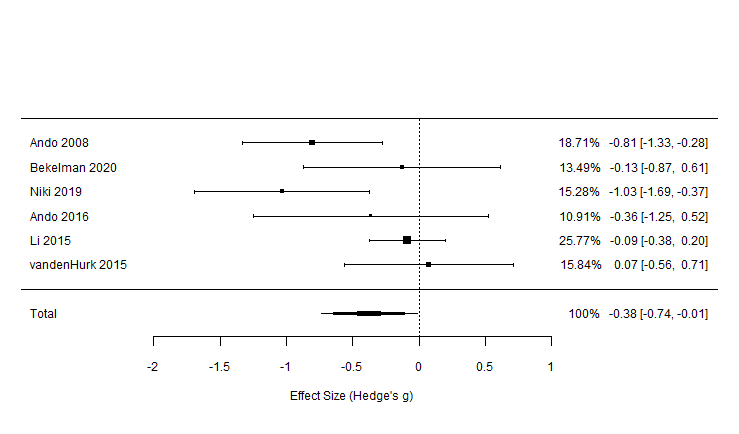


**Study Weight *g* [95% CI]]**

### Appendix G: List of Included Studies

1. [Anderson KO, Mendoza TR, Payne R, Valero V, Palos GR, Nazario A, et al. Pain education for underserved minority cancer patients: a randomized controlled trial. J Clin Oncol. 2004;22:4918–25.](http://paperpile.com/b/heY29X/tqs0)
2. [Ando M, Morita T, Okamoto T, Ninosaka Y. One-week Short-Term Life Review interview can improve spiritual well-being of terminally ill cancer patients. Psychooncology. 2008;17:885–90.](http://paperpile.com/b/heY29X/91zz)
3. [Ando M, Morita T, Akechi T, Okamoto T, Japanese Task Force for Spiritual Care. Efficacy of short-term life-review interviews on the spiritual well-being of terminally ill cancer patients. J Pain Symptom Manage. 2010;39:993–1002.](http://paperpile.com/b/heY29X/Fl3u)
4. [Ando M, Kira H, Hayashida S, Ito S. Effectiveness of the Mindfulness Art Therapy Short Version for Japanese Patients With Advanced Cancer. Art Therapy. Routledge; 2016;33:35–40.](http://paperpile.com/b/heY29X/Myx0)
5. [Bakitas M, Lyons KD, Hegel MT, Balan S, Barnett KN, Brokaw FC, et al. The project ENABLE II randomized controlled trial to improve palliative care for rural patients with advanced cancer: baseline findings, methodological challenges, and solutions. Palliat Support Care. 2009;7:75–86.](http://paperpile.com/b/heY29X/V0Ee)
6. [Bekelman DB, Fink RM, Sannes T, Kline DM, Borrayo EA, Turvey C, et al. Puente para cuidar (bridge to caring): A palliative care patient navigator and counseling intervention to improve distress in Latino/as with advanced cancer. Psychooncology. 2020;29:688–95.](http://paperpile.com/b/heY29X/1FRF)
7. [Bouchard LC, Yanez B, Dahn JR, Flury SC, Perry KT, Mohr DC, et al. Brief report of a tablet-delivered psychosocial intervention for men with advanced prostate cancer: Acceptability and efficacy by race. Transl Behav Med. 2019;9:629–37.](http://paperpile.com/b/heY29X/lSyT)
8. [Caruso R, Sabato S, Nanni MG, Hales S, Rodin G, Malfitano C, et al. Application of managing cancer and living meaningfully (CALM) in advanced cancer patients: An Italian pilot study. Psychother Psychosom. S. Karger AG; 2020;89:402–4.](http://paperpile.com/b/heY29X/mRWZ)
9. [Catania G, Zanini M, Signori A, Dal Molin A, Pilastri P, Bottino M, et al. Providing a nurse-led complex nursing INtervention FOcused on quality of life assessment on advanced cancer patients: The INFO-QoL pilot trial. Eur J Oncol Nurs. 2021;52:101961.](http://paperpile.com/b/heY29X/6aQN)
10. [Chen Y, Xiao H, Zheng J, Zhang X, Lin X. Effects of a mind map-based life review programme on psychospiritual well-being in cancer patients undergoing chemotherapy: A randomised controlled trial. Eur J Cancer Care . Hindawi Limited; 2020;29:e13221.](http://paperpile.com/b/heY29X/P3lK)
11. [Chen Y, Sun L, Xiao H, Zheng J, Lin X. Evaluation of a WeChat-based Dyadic Life Review Program for people with advanced cancer and family caregivers: A mixed-method feasibility study. Palliat Med. 2022;36:498–509.](http://paperpile.com/b/heY29X/NsfE)
12. [Chen Y, Zheng J, Xiao H, Lin X, Zhang X. Effects of a Mind Map-Based Life Review Program on Anxiety and Depressive Symptoms on Cancer Patients Undergoing Chemotherapy: A Randomized Controlled Trial. Cancer Nurs. 2022;45:E116–23.](http://paperpile.com/b/heY29X/wAI9)
13. [Cheung DST, Takemura N, Lam TC, Ho JCM, Deng W, Smith R, et al. Feasibility of Aerobic Exercise and Tai-Chi Interventions in Advanced Lung Cancer Patients: A Randomized Controlled Trial. Integr Cancer Ther. 2021;20:15347354211033352.](http://paperpile.com/b/heY29X/b8Yk)
14. [Cheung DST, Yeung WF, Chau PH, Lam TC, Yang M, Lai K, et al. Patient-centred, self-administered acupressure for Chinese advanced cancer patients experiencing fatigue and co-occurring symptoms: A pilot randomised controlled trial. Eur J Cancer Care . 2022;31:e13314.](http://paperpile.com/b/heY29X/ocEE)
15. [Chimluang J, Thanasilp S, Akkayagorn L, Upasen R, Pudtong N, Tantitrakul W. Effect of an intervention based on basic Buddhist principles on the spiritual well-being of patients with terminal cancer. Eur J Oncol Nurs. 2017;31:46–51.](http://paperpile.com/b/heY29X/6SQj)
16. Costas-Muniz, R, Breitbart, W. [Psychotherapy Intervention for Latinos With Adv Cancer [Internet]. [cited 2023 Jul 15]. Available from:](http://paperpile.com/b/heY29X/xs1l) <https://classic.clinicaltrials.gov/ct2/show/NCT04537936>
17. [Delrieu L, Pialoux V, Pérol O, Morelle M, Martin A, Friedenreich C, et al. Feasibility and Health Benefits of an Individualized Physical Activity Intervention in Women With Metastatic Breast Cancer: Intervention Study. JMIR Mhealth Uhealth. 2020;8:e12306.](http://paperpile.com/b/heY29X/yXlj)
18. [Dionne-Odom JN, Azuero A, Taylor RA, Dosse C, Bechthold AC, Currie E, et al. A lay navigator-led, early palliative care intervention for African American and rural family caregivers of individuals with advanced cancer (Project Cornerstone): Results of a pilot randomized trial. Cancer. 2022;128:1321–30.](http://paperpile.com/b/heY29X/xdZR)
19. [Du J, Fu L, Cui J, An Z, Fang P, Tan L, et al. Effects of the Heart to Heart Card Game for Patients with Advanced Cancer Receiving Home-Based Palliative Care: A Clinical Randomized Controlled Trial. Int J Environ Res Public Health. Multidisciplinary Digital Publishing Institute; 2022;19:6115.](http://paperpile.com/b/heY29X/v0vw)
20. [Fink RM, Kline DM, Siler S, Fischer SM. Apoyo con Cariño: A Qualitative Analysis of a Palliative Care-Focused Lay Patient Navigation Intervention for Hispanics With Advanced Cancer. J Hosp Palliat Nurs. 2020;22:335–46.](http://paperpile.com/b/heY29X/Lq6t)
21. F[ischer SM, Kline DM, Min S-J, Okuyama-Sasaki S, Fink RM. Effect of Apoyo con Cariño (Support With Caring) Trial of a Patient Navigator Intervention to Improve Palliative Care Outcomes for Latino Adults With Advanced Cancer: A Randomized Clinical Trial. JAMA Oncol. 2018;4:1736–41.](http://paperpile.com/b/heY29X/KlPc)
22. [Fraguell-Hernando C, Limonero JT, Gil F. Psychological intervention in patients with advanced cancer at home through Individual Meaning-Centered Psychotherapy-Palliative Care: a pilot study. Support Care Cancer. 2020;28:4803–11.](http://paperpile.com/b/heY29X/rqZZ)
23. [Gil F, Fraguell C, Benito L, Casellas-Grau A, Limonero JT. Meaning-centered psychotherapy integrated with elements of compassion: A pilot study to assess feasibility and utility. Palliat Support Care. 2018;16:643–7.](http://paperpile.com/b/heY29X/ug51)
24. [Han X-B, Fang Y-Q, Liu S-X, Tan Y, Hou J-J, Zhao L-J, et al. Efficacy of combined naikan and morita therapies on psychological distress and posttraumatic growth in Chinese patients with advanced cancer: A randomized controlled trial. Medicine . 2021;100:e26701.](http://paperpile.com/b/heY29X/4cxv)
25. [Hanson LC, Armstrong TD, Green MA, Hayes M, Peacock S, Elliot-Bynum S, et al. Circles of care: development and initial evaluation of a peer support model for African Americans with advanced cancer. Health Educ Behav. 2013;40:536–43.](http://paperpile.com/b/heY29X/S7IX)
26. [Houmann LJ, Rydahl-Hansen S, Chochinov HM, Kristjanson LJ, Groenvold M. Testing the feasibility of the Dignity Therapy interview: adaptation for the Danish culture. BMC Palliat Care. 2010;9:21.](http://paperpile.com/b/heY29X/PS4z)
27. [Houmann LJ, Chochinov HM, Kristjanson LJ, Petersen MA, Groenvold M. A prospective evaluation of Dignity Therapy in advanced cancer patients admitted to palliative care. Palliat Med. 2014;28:448–58.](http://paperpile.com/b/heY29X/ZhnY)
28. [Huang X, Yan A, Liu Q, Wu L. Effects of magnanimous therapy on coping, adjustment, and living function in advanced lung cancer. Curr Oncol. MDPI AG; 2019;26:e48–56.](http://paperpile.com/b/heY29X/UScZ)
29. [Huang X, Liu Q, Li WW, Wu L, Yan A. Effects of magnanimous therapy on emotional, psychosomatic and immune functions of lung cancer patients. J Health Psychol. SAGE Publications; 2021;26:1096–108.](http://paperpile.com/b/heY29X/Xx37)
30. [Ichihara K, Ouchi S, Okayama S, Kinoshita F, Miyashita M, Morita T, et al. Effectiveness of spiritual care using spiritual pain assessment sheet for advanced cancer patients: A pilot non-randomized controlled trial. Palliat Support Care. 2019;17:46–53.](http://paperpile.com/b/heY29X/O7lg)
31. [Julião M, Barbosa A, Oliveira F, Nunes B, Vaz Carneiro A. Efficacy of dignity therapy for depression and anxiety in terminally ill patients: early results of a randomized controlled trial. Palliat Support Care. 2013;11:481–9.](http://paperpile.com/b/heY29X/Q4fc)
32. [Julião M, Oliveira F, Nunes B, Vaz Carneiro A, Barbosa A. Efficacy of dignity therapy on depression and anxiety in Portuguese terminally ill patients: a phase II randomized controlled trial. J Palliat Med. 2014;17:688–95.](http://paperpile.com/b/heY29X/8ula)
33. [Julião M, Oliveira F, Nunes B, Carneiro AV, Barbosa A. Effect of dignity therapy on end-of-life psychological distress in terminally ill Portuguese patients: A randomized controlled trial. Palliat Support Care. 2017;15:628–37.](http://paperpile.com/b/heY29X/MF8k)
34. [Kang K-A, Kim S-J. Development and Preliminary Testing of a Meaning-Centered Program for Young Adults With Advanced-Stage Cancer. J Hosp Palliat Nurs. 2015;17:213.](http://paperpile.com/b/heY29X/0dZb)
35. [Kim HJ, Kim SM, Shin H, Jang J-S, Kim YI, Han DH. A Mobile Game for Patients With Breast Cancer for Chemotherapy Self-Management and Quality-of-Life Improvement: Randomized Controlled Trial. J Med Internet Res. 2018;20:e273.](http://paperpile.com/b/heY29X/3cXF)
36. [Kwan CWM, Chan CWH, Choi KC. The effectiveness of a nurse-led short term life review intervention in enhancing the spiritual and psychological well-being of people receiving palliative care: A mixed method study. Int J Nurs Stud. 2019;91:134–43.](http://paperpile.com/b/heY29X/pPgV)
37. [Landa-Ramírez E, Greer JA, Sánchez-Román S, Manolov R, Salado-Avila MM, Templos-Esteban LA, et al. Tailoring Cognitive Behavioral Therapy for Depression and Anxiety Symptoms in Mexican Terminal Cancer Patients: A Multiple Baseline Study. J Clin Psychol Med Settings. 2020;27:54–67.](http://paperpile.com/b/heY29X/IH19)
38. [Lee CE, Kim S, Kim S, Joo HM, Lee S. Effects of a Mindfulness-Based Stress Reduction Program on the Physical and Psychological Status and Quality of Life in Patients With Metastatic Breast Cancer. Holist Nurs Pract. 2017;31:260–9.](http://paperpile.com/b/heY29X/YHvI)
39. Leng J, Lui F, Chen A, Huang X, Breitbart W, Gany F. Adapting meaning-centered psychotherapy in advanced cancer for the Chinese immigrant population. Journal of immigrant and minority health. 2018 Jun;20:680-6.
40. [Leng J, Lui F, Huang X, Breitbart W, Gany F. Patient perspectives on adapting meaning-centered psychotherapy in advanced cancer for the Chinese immigrant population. Support Care Cancer. 2019;27:3431–8.](http://paperpile.com/b/heY29X/jAjj)
41. [Li H-C, Richardson A, Speck P, Armes J. Conceptualizations of dignity at the end of life: exploring theoretical and cultural congruence with dignity therapy. J Adv Nurs. 2014;70:2920–31.](http://paperpile.com/b/heY29X/qwrS)
42. [Li Q, Xu Y, Zhou H, Loke AY. A couple-based complex intervention for Chinese spousal caregivers and their partners with advanced cancer: an intervention study. Psychooncology. Wiley; 2015;24:1423–31.](http://paperpile.com/b/heY29X/MalL)
43. [Li Y, Ling L, Zhanyu P. Effect of Wellness Education on Quality of Life of Patients With Non-Small Cell Lung Cancer Treated With First-Line Icotinib and on Their Family Caregivers. Integr Cancer Ther. 2019;18:1534735419842373.](http://paperpile.com/b/heY29X/J1SY)
44. [Li Y-C, Feng Y-H, Chiang H-Y, Ma S-C, Wang H-H. The Effectiveness of Dignity Therapy as Applied to End-of-Life Patients with Cancer in Taiwan: A Quasi-Experimental Study. Asian Nurs Res . 2020;14:189–95.](http://paperpile.com/b/heY29X/Radf)
45. [Liao J, Yang Y-F, Cohen L, Zhao Y-C, Xu Y. Effects of Chinese medicine five-element music on the quality of life for advanced cancer patients: a randomized controlled trial. Chin J Integr Med. 2013;19:736–40.](http://paperpile.com/b/heY29X/UqKx)
46. [Lin C-P, Evans CJ, Koffman J, Chen P-J, Hou M-F, Harding R. Feasibility and acceptability of a culturally adapted advance care planning intervention for people living with advanced cancer and their families: A mixed methods study. Palliat Med. 2020;34:651–66.](http://paperpile.com/b/heY29X/wii5)
47. [Lin J, Zhao Y, Guo Q. Dignity therapists’ experience of conducting dignity therapy with terminal cancer patients in mainland China: A descriptive qualitative study. Eur J Cancer Care . 2022;31:e13670.](http://paperpile.com/b/heY29X/Q93A)
48. [Liossi C, White P. Efficacy of clinical hypnosis in the enhancement of quality of life of terminally ill cancer patients. Contemp Hypn. Wiley; 2001;18:145–60.](http://paperpile.com/b/heY29X/FLU9)
49. [Matthys O, De Vleminck A, Dierickx S, Deliens L, Van Goethem V, Lapeire L, et al. Effectiveness of a nurse-delivered (FOCUS+) and a web-based (iFOCUS) psychoeducational intervention for people with advanced cancer and their family caregivers (DIAdIC): study protocol for an international randomized controlled trial. BMC Palliat Care. 2021;20:193.](http://paperpile.com/b/heY29X/zV4C)
50. [Maungtoug N, Othaganont P, Liehr P. Adding Ritualized Chanting to the Palliative Care of Cancer Patients at the End of Life: A Randomized Controlled Trial. J Soc Work End Life Palliat Care. 2021;17:35–49.](http://paperpile.com/b/heY29X/B4qS)
51. [Miyamoto S, Yamazaki T, Shimizu K, Matsubara T, Kage H, Watanabe K, et al. Brief, manualised and semistructured individual psychotherapy programme for patients with advanced cancer in Japan: study protocol for Managing Cancer and Living Meaningfully (CALM) phase 2 trial. BMJ Open. 2022;12:e056136.](http://paperpile.com/b/heY29X/ZboP)
52. [Molassiotis A, Roberts S, Cheng HL, To HKF, Ko PS, Lam W, et al. Partnering with families to promote nutrition in cancer care: feasibility and acceptability of the PIcNIC intervention. BMC Palliat Care. 2018;17:50.](http://paperpile.com/b/heY29X/OtJo)
53. [Molassiotis A, Brown T, Cheng HL, Byrnes A, Chan RJ, Wyld D, et al. The effects of a family-centered psychosocial-based nutrition intervention in patients with advanced cancer: the PiCNIC2 pilot randomised controlled trial. Nutr J. 2021;20:2.](http://paperpile.com/b/heY29X/W8Wn)
54. [Nakayama H, Kikuta F, Takeda H. A pilot study on effectiveness of music therapy in hospice in Japan. J Music Ther. 2009;46:160–72.](http://paperpile.com/b/heY29X/QsSe)
55. [Niki K, Okamoto Y, Maeda I, Mori I, Ishii R, Matsuda Y, et al. A Novel Palliative Care Approach Using Virtual Reality for Improving Various Symptoms of Terminal Cancer Patients: A Preliminary Prospective, Multicenter Study. J Palliat Med. 2019;22:702–7.](http://paperpile.com/b/heY29X/uzQu)
56. [Nunziante F, Tanzi S, Alquati S, Autelitano C, Bedeschi E, Bertocchi E, et al. Providing dignity therapy to patients with advanced cancer: a feasibility study within the setting of a hospital palliative care unit. BMC Palliat Care. 2021;20:129.](http://paperpile.com/b/heY29X/VxAl)
57. [Ólafsdóttir KL, Jónsdóttir H, Fridriksdóttir N, Sigurdardóttir V, Haraldsdóttir E. Integrating nurse-facilitated advance care planning for patients newly diagnosed with advanced lung cancer. Int J Palliat Nurs. 2018;24:170–7.](http://paperpile.com/b/heY29X/pz3u)
58. [Onyechi KCN, Onuigbo LN, Eseadi C, Ikechukwu-Ilomuanya AB, Nwaubani OO, Umoke PCI, et al. Effects of Rational-Emotive Hospice Care Therapy on Problematic Assumptions, Death Anxiety, and Psychological Distress in a Sample of Cancer Patients and Their Family Caregivers in Nigeria. Int J Environ Res Public Health [Internet]. 2016;13. Available from:](http://paperpile.com/b/heY29X/NlCv) <http://dx.doi.org/10.3390/ijerph13090929>
59. [Park S, Kim K, Ahn HK, Kim JW, Min G, Chung BH, et al. Impact of Lifestyle Intervention for Patients with Prostate Cancer. Am J Health Behav. 2020;44:90–9.](http://paperpile.com/b/heY29X/H9Fw)
60. [Patel M, Andrea N, Jay B, Coker TR. A Community-Partnered, Evidence-Based Approach to Improving Cancer Care Delivery for Low-Income and Minority Patients with Cancer. J Community Health. 2019;44:912–20.](http://paperpile.com/b/heY29X/wPI9)
61. [Patel MI, Khateeb S, Coker T. A randomized trial of a multi-level intervention to improve advance care planning and symptom management among low-income and minority employees diagnosed with cancer in outpatient community settings. Contemp Clin Trials. 2020;91:105971.](http://paperpile.com/b/heY29X/QqRn)
62. [Patel MI, Khateeb S, Coker T. Association of a Lay Health Worker-Led Intervention on Goals of Care, Quality of Life, and Clinical Trial Participation Among Low-Income and Minority Adults With Cancer. JCO Oncol Pract. 2021;17:e1753–62.](http://paperpile.com/b/heY29X/MVKH)
63. [Pon AKL. My Wonderful Life: A Board Game for Patients with Advanced Cancer. Illness, Crisis & Loss. SAGE Publications Inc; 2010;18:147–61.](http://paperpile.com/b/heY29X/N5ae)
64. [Quílez-Bielsa E, Barrado-Moreno V, Lastra Del Prado R, Arbonés-Mainar JM, Sebastian-Sanchez M, Ventura-Faci T. An adaptation of meaning-centered psychotherapy integrating “essential care”: A pilot study. Palliat Support Care. 2022;20:496–504.](http://paperpile.com/b/heY29X/EDMx)
65. [Ramos K, Hastings SN, Bosworth HB, Fulton JJ. Life Program: Pilot Testing a Palliative Psychology Group Intervention. J Palliat Med. 2018;21:1641–5.](http://paperpile.com/b/heY29X/EHCz)
66. [Sakaguchi S, Okamura H. Effectiveness of collage activity based on a life review in elderly cancer patients: A preliminary study. Palliat Support Care. 2015;13:285–93.](http://paperpile.com/b/heY29X/ylF1)
67. [Scheffold K, Philipp R, Engelmann D, Schulz-Kindermann F, Rosenberger C, Oechsle K, et al. Efficacy of a brief manualized intervention Managing Cancer and Living Meaningfully (CALM) adapted to German cancer care settings: study protocol for a randomized controlled trial. BMC Cancer. 2015;15:592.](http://paperpile.com/b/heY29X/f0ow)
68. [Schulman-Green D, Feder SL, Collett D, Aaron EM, Haron Y, Eilon Y, et al. Adapting a palliative care-focused cancer self- and family management intervention for use in Israel. Int J Palliat Nurs. 2022;28:378–87.](http://paperpile.com/b/heY29X/0j74)
69. [Takenouchi S, Chikada A, Mori M, Tamura K, Nin K. Strategies to Understand What Matters to Advanced Cancer Patients in Advance Care Planning: A Qualitative Study Using the Lifeline Interview Method. J Hosp Palliat Nurs. 2022;24:E135–43.](http://paperpile.com/b/heY29X/7jRd)
70. [Teo I, Vilardaga JP, Tan YP, Winger J, Cheung YB, Yang GM, et al. A feasible and acceptable multicultural psychosocial intervention targeting symptom management in the context of advanced breast cancer. Psychooncology. 2020;29:389–97.](http://paperpile.com/b/heY29X/Keqr)
71. [Teo I, Tan YP, Finkelstein EA, Yang GM, Pan FT, Lew HYF, et al. The Feasibility and Acceptability of a Cognitive Behavioral Therapy-Based Intervention for Patients With Advanced Colorectal Cancer. J Pain Symptom Manage. 2020;60:1200–7.](http://paperpile.com/b/heY29X/WtjL)
72. Torres-[Blasco N, Muñiz RC, Zamore C, Porter L, Claros M, Bernal G, et al. Cultural adaptation of meaning-centered psychotherapy for latino families: a protocol. BMJ Open. British Medical Journal Publishing Group; 2022;12:e045487.](http://paperpile.com/b/heY29X/RvIF)
73. [Torres-Blasco N, Costas-Muñiz R, Rosario L, Porter L, Suárez K, Peña-Vargas C, et al. Psychosocial Intervention Cultural Adaptation for Latinx Patients and Caregivers Coping with Advanced Cancer. Healthcare (Basel) [Internet]. 2022;10. Available from:](http://paperpile.com/b/heY29X/sSG9) <http://dx.doi.org/10.3390/healthcare10071243>
74. [van den Hurk DGM, Schellekens MPJ, Molema J, Speckens AEM, van der Drift MA. Mindfulness-Based Stress Reduction for lung cancer patients and their partners: Results of a mixed methods pilot study. Palliat Med. 2015;29:652–60.](http://paperpile.com/b/heY29X/SNlJ)
75. [van der Wel M, van der Smissen D, Dierickx S, Cohen J, Hudson P, De Vleminck A, et al. Systematic translation and adaptation of the FOCUS program, a USA-based supportive intervention for persons with cancer and their family caregivers, for use in six European countries. Support Care Cancer. 2022;30:9763–70.](http://paperpile.com/b/heY29X/PzgV)
76. [Wang C, Chen J, Wang Y, Hu R, Wu Y. The development of a family participatory dignity therapy programme for patients with haematologic neoplasms and their family caregivers in China: A feasibility study. Eur J Cancer Care . 2020;29:e13204.](http://paperpile.com/b/heY29X/iizL)
77. [Warth M, Kessler J, van Kampen J, Ditzen B, Bardenheuer HJ. “Song of Life”: music therapy in terminally ill patients with cancer. BMJ Support Palliat Care. 2018;8:167–70.](http://paperpile.com/b/heY29X/U7zr)
78. [Xiao H, Kwong E, Pang S, Mok E. Perceptions of a life review programme among Chinese patients with advanced cancer. J Clin Nurs. 2012;21:564–72.](http://paperpile.com/b/heY29X/Muew)
79. [Xiao H, Kwong E, Pang S, Mok E. Effect of a life review program for Chinese patients with advanced cancer: a randomized controlled trial. Cancer Nurs. 2013;36:274–83.](http://paperpile.com/b/heY29X/3h2W)
80. [Xiao J, Chow KM, Choi KC, Ng SNM, Huang C, Ding J, et al. Effects of family-oriented dignity therapy on dignity, depression and spiritual well-being of patients with lung cancer undergoing chemotherapy: A randomised controlled trial. Int J Nurs Stud. 2022;129:104217.](http://paperpile.com/b/heY29X/jF6A)
81. [Yanez B, McGinty HL, Mohr DC, Begale MJ, Dahn JR, Flury SC, et al. Feasibility, acceptability, and preliminary efficacy of a technology-assisted psychosocial intervention for racially diverse men with advanced prostate cancer. Cancer. Wiley; 2015;121:4407–15.](http://paperpile.com/b/heY29X/ZnhP)
82. [Yang GM, Dionne-Odom JN, Foo YH, Chung AHM, Kamal NHA, Tan L, et al. Adapting ENABLE for patients with advanced cancer and their family caregivers in Singapore: a qualitative formative evaluation. BMC Palliat Care. 2021;20:86.](http://paperpile.com/b/heY29X/s54C)
83. [Ye ZJ, Qiu HZ, Liang MZ, Liu ML, Li PF, Chen P, et al. Effect of a mentor-based, supportive-expressive program, Be Resilient to Breast Cancer, on survival in metastatic breast cancer: a randomised, controlled intervention trial. Br J Cancer. Nature Publishing Group; 2017;117:1486–94.](http://paperpile.com/b/heY29X/7w0p)
84. [Zhang X, Xiao H. Development and evaluation of a WeChat-based life review programme for patients with cancer: protocol for a randomised controlled trial. BMJ Open. 2018;8:e020239.](http://paperpile.com/b/heY29X/AQoH)
85. [Zhang X, Xiao H, Chen Y. Evaluation of a WeChat-based life review programme for cancer patients: A quasi-experimental study. J Adv Nurs. Wiley; 2019;75:1563–74.](http://paperpile.com/b/heY29X/AVPq)
86. [Zheng X, Peng Y, Liu C, Li F, Zhang H, Liao J, et al. Application of VIP Care in Patients with Advanced Tumors in the Western Region of China. Biomed Res Int. 2022;2022:7834620.](http://paperpile.com/b/heY29X/Lg6Q)
